# *Bacillus subtilis*-derived peptides disrupt quorum sensing and biofilm assembly in multidrug-resistant *Staphylococcus aureus*

**DOI:** 10.1128/msystems.00712-24

**Published:** 2024-07-11

**Authors:** Kyle R. Leistikow, Daniel S. May, Won Se Suh, Gabriel Vargas Asensio, Adam J. Schaenzer, Cameron R. Currie, Krassimira R. Hristova

**Affiliations:** 1 Department of Biological Sciences, Marquette University, Milwaukee, Wisconsin, USA; 2 Department of Bacteriology, University of Wisconsin-Madison, Madison, Wisconsin, USA; 3 Department of Chemistry, Washington College, Chestertown, Maryland, USA; 4 Department of Biochemistry & Biomedical Sciences, McMaster University, Hamilton, Ontario, Canada; University of Illinois at Chicago, Chicago, Illinois, USA

**Keywords:** *Bacillus subtilis*, *Staphylococcus aureus*, biofilm, antibiotic resistance, quorum sensing interference, peptide, probiotic, postbiotic

## Abstract

Multidrug-resistant *Staphylococcus aureus* is one of the most clinically important pathogens in the world, with infections leading to high rates of morbidity and mortality in both humans and animals. The ability of *S. aureus* to form biofilms protects cells from antibiotics and promotes the transfer of antibiotic resistance genes; therefore, new strategies aimed at inhibiting biofilm growth are urgently needed. Probiotic species, including *Bacillus subtilis,* are gaining interest as potential therapies against *S. aureus* for their ability to reduce *S. aureus* colonization and virulence. Here, we search for strains and microbially derived compounds with strong antibiofilm activity against multidrug-resistant *S. aureus* by isolating and screening *Bacillus* strains from a variety of agricultural environments. From a total of 1,123 environmental isolates, we identify a single strain *B. subtilis* 6D1, with a potent ability to inhibit biofilm growth, disassemble mature biofilm, and improve antibiotic sensitivity of *S. aureus* biofilms through an Agr quorum sensing interference mechanism. Biochemical and molecular networking analysis of an active organic fraction revealed multiple surfactin isoforms, and an uncharacterized peptide was driving this antibiofilm activity. Compared with commercial high-performance liquid chromatography grade surfactin obtained from *B. subtilis*, we show these *B. subtilis* 6D1 peptides are significantly better at inhibiting biofilm formation in all four *S*. *aureus* Agr backgrounds and preventing *S. aureus*-induced cytotoxicity when applied to HT29 human intestinal cells. Our study illustrates the potential of exploring microbial strain diversity to discover novel antibiofilm agents that may help combat multidrug-resistant *S. aureus* infections and enhance antibiotic efficacy in clinical and veterinary settings.

**IMPORTANCE:**

The formation of biofilms by multidrug-resistant bacterial pathogens, such as *Staphylococcus aureus*, increases these microorganisms’ virulence and decreases the efficacy of common antibiotic regimens. Probiotics possess a variety of strain-specific strategies to reduce biofilm formation in competing organisms; however, the mechanisms and compounds responsible for these phenomena often go uncharacterized. In this study, we identified a mixture of small probiotic-derived peptides capable of Agr quorum sensing interference as one of the mechanisms driving antibiofilm activity against *S. aureus*. This collection of peptides also improved antibiotic killing and protected human gut epithelial cells from *S. aureus*-induced toxicity by stimulating an adaptive cytokine response. We conclude that purposeful strain screening and selection efforts can be used to identify unique probiotic strains that possess specially desired mechanisms of action. This information can be used to further improve our understanding of the ways in which probiotic and probiotic-derived compounds can be applied to prevent bacterial infections or improve bacterial sensitivity to antibiotics in clinical and agricultural settings.

## INTRODUCTION

According to the World Health Organization, antimicrobial-resistant (AMR) bacterial infections accounted for an estimated 700,000 deaths worldwide in 2019, a number that is expected to surpass 10 million by 2050 if immediate measures are not taken (1). In the United States alone, 2.5 million AMR infections resulted in an estimated annual economic cost of more than $55 billion (2). The increasing global expansion of AMR infections in both agriculture and hospital settings warrants the use of alternative therapies to help reduce the use of antibiotics and decrease the dissemination of antibiotic resistance genes in these environments. Antibiotic-resistant *Staphylococcus aureus* is a leading cause of pneumonia, sepsis, endocarditis, and soft tissue infections. In addition to its constantly evolving resistance to front-line antibiotics, *S. aureus* infections are made more difficult to treat due to its biofilm formation abilities (3). Biofilms are an aggregated heterogeneous community of cells surrounded by an extracellular polymeric matrix that can increase antibiotic resistance 1,000-fold (4). Thus, innovative approaches to combat biofilm-related *S. aureus* infections are urgently needed. A variety of synthetic compounds have been investigated for their ability to reduce *S. aureus* biofilm growth (5–7); however, limited research has explored the effects of probiotics and probiotic-derived small molecules on pathogenic biofilms. Probiotics possess a variety of strain-specific strategies to reduce pathogen growth, virulence, and/or biofilm formation and have shown promise in preventing and treating a variety of diseases (8, 9), including *S. aureus* infections (10). However, the benefit of probiotic use to reduce antimicrobial resistance in the environment is largely understudied (11). The majority of probiotic research has sought to identify strains with broad bactericidal activity (12); however, recent evidence suggests that some strains can reduce disease outcomes by inhibiting pathogen quorum sensing, a population-dependent bacterial communication system that modulates gene expression and lifestyle selection. Quorum sensing inhibitors do not kill bacteria; instead, they interfere with communication systems needed to form biofilms and produce virulence factors. Quorum sensing interference (QSI) has also been proposed as a method to improve antibiotic-killing effects (13, 14), making it an intriguing mechanism with immediate therapeutic potential.

In *S. aureus,* the Agr quorum sensing (QS) system orchestrates virulence gene expression through the secretion and self-recognition of post-translationally modified autoinducing peptides (AIPs). Production and recognition of these AIPs by the Agr system govern the transition between *S. aureus* colonization and invasive infection (15). More specifically, the *agrBDCA* operon, integral to a functioning Agr QS system, allows *S. aureus* to coordinate the formation and disassembly of biofilm and is required for approximately 90% of *S. aureus* infections (16). The Agr QS system is conserved among *S. aureus* strains, but variations within AgrD and the C-terminus of AgrB result in four different mature AIPs that are uniquely recognized by four differently configured AgrC receptors (17). Each of the four *S*. *aureus* Agr groups has a different biological consequence to the host: Agr-I is linked to enterotoxin disease; Agr-II is linked to early vancomycin-resistance and endocarditis; Agr-III is linked to endocarditis and menstrual toxic shock syndrome; and Agr-IV is linked to exfoliative disease (15, 18). Despite these differences, the Agr QS system in each group helps regulate virulence factor gene expression (19) and facilitate biofilm formation and dispersal (20, 21). When the Agr QS system is turned on, *S. aureus* maintains a planktonic lifestyle. Conversely, when the Agr QS system is turned off, *S. aureus* forms a biofilm. Importantly, this phenomenon can be impeded by competitive Agr signaling interference (22). This occurs when peptides produced by different species bind to AgrC and successfully initiate the quorum sensing pathways in neighboring cells (23, 24). Therefore, it is possible that certain probiotic strains produce peptides that reduce *S. aureus* biofilm formation through an activation of the Agr QS system (25).

One probiotic species, *Bacillus subtilis*, is gaining interest as a potential therapeutic against *S. aureus*. Although the potential health benefits associated with *B. subtilis* are well documented, the reproducibility of these interventions largely depends on the *B. subtilis* strain used (26, 27). *B. subtilis* strains are remarkably diverse and shaped both by their environment and their ability to acquire genes from closely related species (28, 29). Probiotic *Bacillus* species have been isolated from a variety of dairy products and fermented foods, both terrestrial and aquatic plants and animals, cropland soils, and the human gastrointestinal tract (30–33). Therefore, due to *B. subtilis’* diverse ecological range, it is perhaps not surprising that strain-specific genetic elements have evolved to help this species compete in a variety of environments (34, 35). *B. subtilis* excretes a variety of compounds (36) that might inhibit *S. aureus* biofilm growth (37); however, the precise compounds driving these phenomenon often go unidentified (25).

The overall aim of this study was to explore *B. subtilis* strain diversity and identify environmental isolates with potent antibiofilm activity against multi-drug-resistant *S. aureus*. The main objectives were to investigate the mechanisms of probiotic *B. subtilis* antagonism against *S. aureus*, identify the chemical signatures of probiotic compounds with antagonistic activity, and test their effects on *S. aureus* virulence and susceptibility to clinically important antibiotics. Interspecies competition is known to drive the evolution of enhanced competitive strategies and selfish quorum sensing phenotypes in polymicrobial environments (38–40). Therefore, we hypothesized that potential probiotic *Bacillus* strains obtained from these agricultural environments had likely co-evolved to compete with a variety of dairy pathogens, namely *S. aureus*, the primary etiological agent responsible for bovine mastitis (41, 42). In this study, we isolate and screen a library of 1,123 *Bacillus* strains sourced from a variety of contaminated dairy environments for antibiofilm activity against methicillin-resistant and methicillin-sensitive *S. aureus*. To investigate the potential genetic mechanisms driving *S. aureus* antagonism, we performed phylogenomic and pangenome analyses using *B. subtilis* 6D1, a strain with especially potent antibiofilm activity against *S. aureus*. We further investigated how this strain inhibits *S. aureus* biofilm growth, identified the compound(s) driving this activity, and then assessed these compounds’ protective effects against *S. aureus* infection in eukaryotic cell culture experiments. Employing purposeful probiotic screening strategies, investigating the strain-mediated mechanisms that reduce virulence and improve antibiotic efficacy, and subsequently identifying the strain-derived products responsible are essential to understand how probiotic microorganisms can be deployed to combat multidrug-resistant pathogens and help reduce the use of antibiotics intended to treat these pathogen-related infections.

## RESULTS

### *B. subtilis* 6D1 exhibits antibiofilm activity against *S. aureus* and harbors unique genetic traits not observed in closely related *B. subtilis* strains

**Fig 1:**
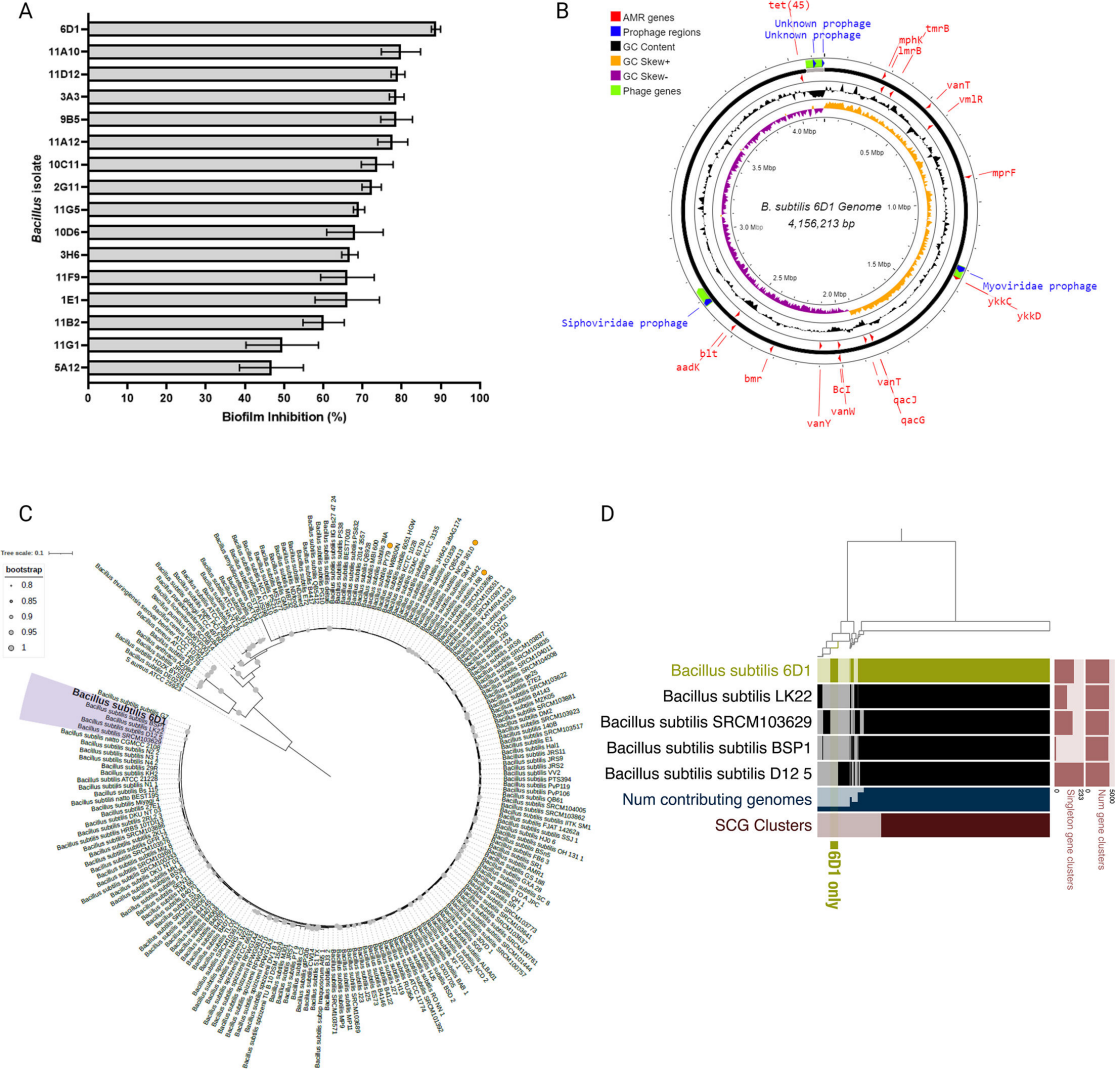
*B. subtilis* 6D1 exhibits antibiofilm activity against *S. aureus* and harbors unique genetic traits not observed in closely related *B. subtilis* strains. (**A**) Antibiofilm activity of 16 *Bacillus* cell-free extracts against *S. aureus* 29213. Error bars represent mean ± SD of eight independent replicates. (**B**) The assembled *B. subtilis* 6D1 genome (**C**) *B. subtilis* 6D1 shares > 95% identity with a group of *B. subtilis* genomes (purple) and is more distantly related to common laboratory type strains (yellow dots). (**D**) Pangenome analysis identified 156 unique genes (green) in *B. subtilis* 6D1 that were not detected in any strain from this closely related group. Singleton gene clusters identified range from 0 to 233, and the number of gene clusters identified range from 0 to 5,000.

High-throughput screening of 1,123 presumptive *Bacillus* strains revealed that 45 isolates exhibited antagonistic activity against *S. aureus* in drop diffusion and cross-streak assays. Cell-free extracts were then obtained from each of these 45 strains and assayed for antibiofilm activity against 38 clinical *S. aureus* strains (Table S1). *Bacillus* strains (*n* = 16) exhibiting broad antibiofilm activity were assayed for relevant pathogenic *Bacillus* toxin genes (43) prior to sequencing the 16S rRNA gene region for taxonomic classification (44, 45). Among these 16 candidates, a single strain, *B. subtilis* 6D1, exhibited the strongest antibiofilm activity against *S. aureus* and thus was selected for more detailed investigations (Fig. 1A). The genomic diversity among *B. subtilis* strains can be leveraged to understand how certain strain elements contribute to their antimicrobial production potential (35). Therefore, whole genome sequencing, phylogenomic analysis, and pangenome analyses of *B. subtilis* 6D1 were performed to search for unique genomic traits that might explain this strain’s prominent antibiofilm activity. Genomic assembly yielded two contigs—the 4 MB chromosome and an 84 kB plasmid similar to pBS32, a large ancestral plasmid rarely found in environmental isolates(46, 47) (Fig. 1B). The entire genome contained 4,156,213 base pairs with 43.65% guanine-cytosine (GC) content. A total of 4,309 protein-coding genes (CDS), 88 transfer RNA (tRNA) genes, and 30 ribosomal RNA (rRNA) genes were predicted. Of the 4,309 CDS predicted, 708 were proteins of unknown function, and 3,601 proteins had functional assignments—these included 1,055 proteins with Enzyme Commission (EC) numbers, 881 with Gene Ontology (GO) assignments, and 776 proteins that were mapped to Kyoto Encyclopedia of Genes and Genomes (KEGG) pathways. Sixteen antimicrobial resistance genes were identified only in the chromosome and were not flanked by any predicted mobile genetic elements (Fig. 1B).

Taxonomic identification and phylogenetic analysis confirmed this genome was a member of the *B. subtilis* group and was most similar to *B. subtilis* 75, and *B. subtilis* PTA-271, strains sourced from the rhizosphere of plant species capable of reducing plant pathogen colonization (Fig. S2). Phylogenomic analysis revealed *B. subtilis* 6D1 shares > 95% identity with a recently evolved group of *B. subtilis* genomes (Fig. 1C); however, this strain encodes an additional 156 singleton genes that were not present in these closely related strains (Fig. 1D; Table S3). Many of the singleton genes were of unknown function, but genes encoding a glycosyl hydrolase were identified. This class of enzymes can hydrolyze the glycosidic bonds between sugars, such as those found within the exopolysaccharides of biofilm matrices, and have been shown to reduce biofilm biomass by weakening the matrix and inducing bacterial dispersal (48, 49). Interestingly, glycosyl hydrolases can inhibit and degrade Staphylococcal and Pseudomonal biofilms and improve clearance of these pathogens when applied in conjunction with antibiotics (49). The second largest group of singleton genes identified were predicted to be of viral origin, including prophage genes from the family Siphoviridae. Genome mapping further confirmed the presence and location of a 25 kB and 27 kB prophage region predicted to belong to the Siphoviridae and Myoviridae family, respectively (Fig. 1B). The *B. subtilis* 6D1 strain appeared to maintain many ancestral *B. subtilis* genes (Fig. 1D), including a pBS32-like plasmid (Fig. 1B); however, our results suggest this strain may have recently acquired foreign genetic elements, possibly from phage, that increased its ability to degrade pathogenic biofilms.

### *B. subtilis* 6D1 inhibits *S. aureus* biofilm growth but not planktonic growth

**Fig 2:**
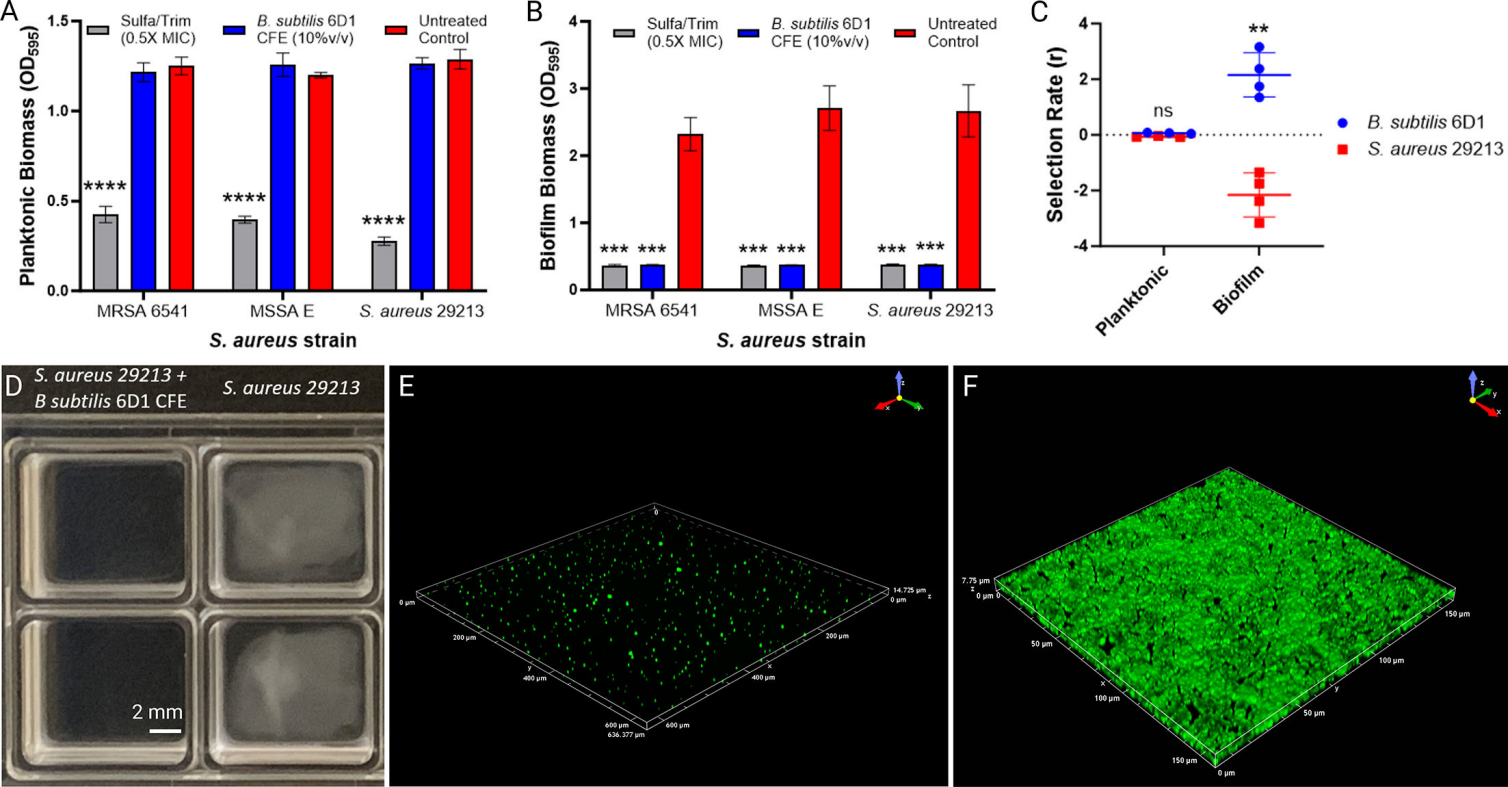
*B. subtilis* 6D1 inhibits *S. aureus* biofilm but not planktonic growth. (**A and B**) Compared with 0.5× MIC of Sulfamethoxazole/Trimethoprim, addition of 10% vol/vol *B. subtilis* 6D1 cell-free extract inhibits methicillin-susceptible (MSSA) and methicillin-resistant (MRSA) *S. aureus* biofilm growth, but not planktonic growth. Error bars represent mean ± SD of 12 independent replicates. Differences in biofilm inhibition were analyzed using a 2-tailed unpaired *t*-test where (***) *P* < 0.001, (****) *P* < 0.0001 compared with the untreated control. (**C**) Competition experiments performed in planktonic and biofilm conditions for 24 hours show *B. subtilis* 6D1 outcompetes *S. aureus* in a biofilm but not in a planktonic environment. Error bars represent mean ± SD of four independent replicates (**) *P* < 0.01. (**D**) Macroscopic observation of *S. aureus* ATCC 29213 biofilms grown for 24 hours and washed prior to SYTO 9 staining (**E**) SYTO 9 staining visualized by confocal microscopy confirmed antibiofilm activity of 10% vol/vol *B. subtilis* 6D1 CFE (**F**) compared with an untreated control of *S. aureus* ATCC 29213.

To further explore the mechanisms by which *B. subtilis* 6D1 inhibits *S. aureus* biofilms, cell-free extracts (CFEs) were applied to clinical methicillin-susceptible and methicillin-resistant *S. aureus* strains and evaluated for antimicrobial and antibiofilm activity. Compared with 0.5× MIC sulfamethoxazole/trimethoprim, *B. subtilis* 6D1 CFE applied at 10% vol/vol (~1 mg/mL protein concentration) significantly reduced biofilm formation of *S. aureus* ATCC 29213 and both clinical strains without inhibiting planktonic growth (Fig. 2A and B). Competition experiments in planktonic and biofilm environments also revealed *B. subtilis* 6D1 outcompetes *S. aureus* ATCC 29213 in a biofilm but not in a planktonic environment (Fig. 2C). These data suggest *B. subtilis* 6D1 inhibits *S. aureus* biofilm formation during competitive interactions, confirming that antibiofilm activity is not simply an artifact of monoculture CFE preparations. Macroscopic observation of *S. aureus* ATCC 29213 biofilms grown in the presence of CFE for 24 hours further supported our initial observations (Fig. 2D). Confocal image analysis confirmed antibiofilm activity of 10% vol/vol *B. subtilis* 6D1 CFE, reducing mean SYTO 9 intensity 77% compared with untreated wells (Fig. 2E and F). Subsequent size fractionation analyses revealed antibiofilm activity was maintained in CFE fractions < 3 kDa (Fig. S1), suggesting small peptides, rather than glycosyl hydrolases, are likely driving this effect. Taken together, these results demonstrate compounds produced by *B. subtilis* 6D1 inhibit biofilm formation of *S. aureus*.

### *B. subtilis* 6D1 cell-free extracts reduce *S. aureus* biofilm growth, disassemble mature biofilm, and improve biofilm inhibition when applied in conjunction with low doses of antibiotics

**Fig 3:**
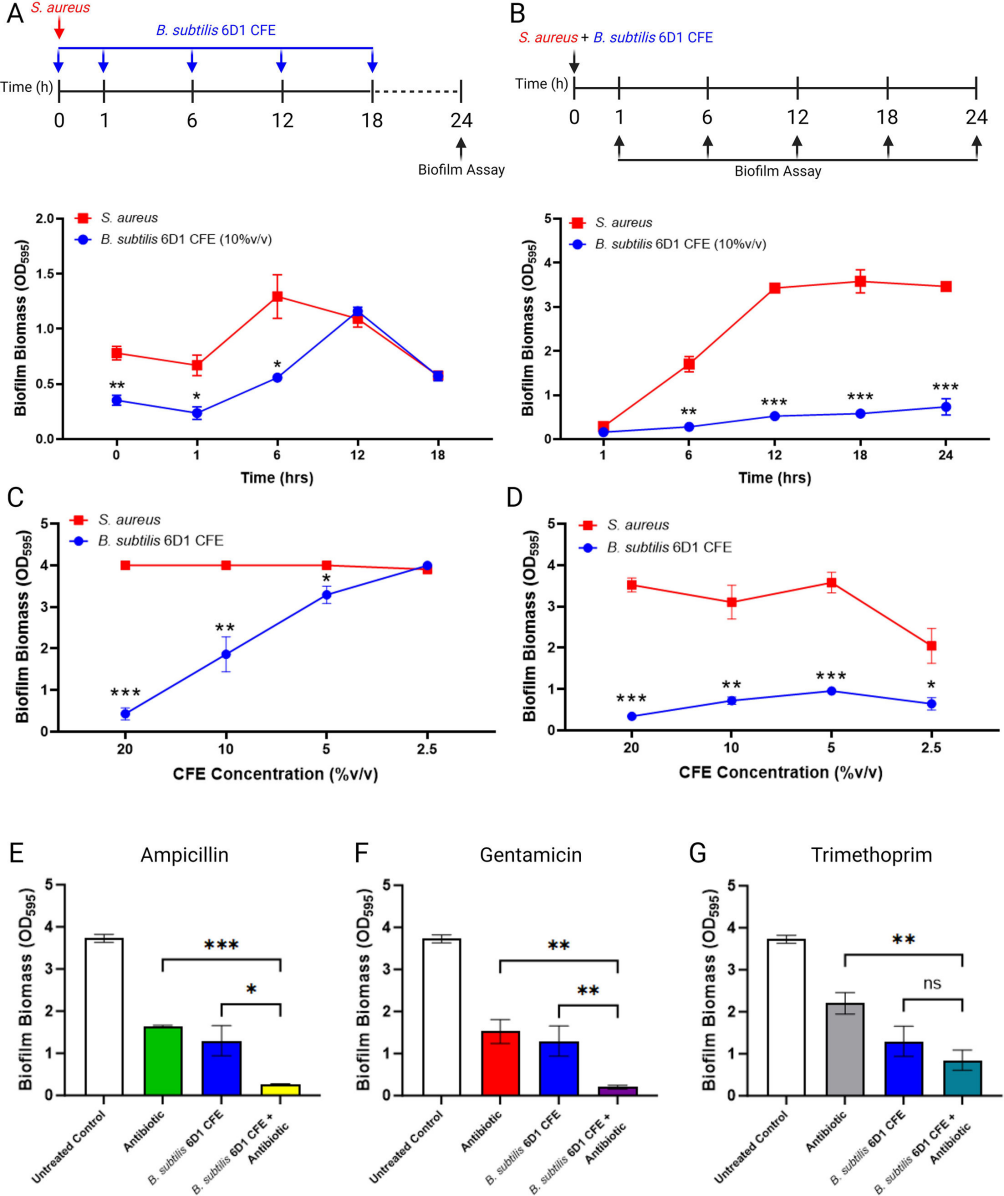
*B. subtilis* 6D1 cell-free extracts (CFEs) reduce *S. aureus* ATCC 29213 biofilm growth, disassemble mature biofilm, and improve biofilm inhibition when applied in conjunction with low doses of antibiotics. (**A**) *S. aureus* (5 × 10^5^ CFU/mL) was grown on polystyrene plates at 37°C for 0, 1, 6, 12, or 18 hours, followed by treatment with *B. subtilis* 6D1 CFE (10% vol/vol) at 37°C for up to 24 hours (**B**) *S. aureus* (5 × 10^5^ CFU/mL) was grown on polystyrene plates at 37°C for 1, 6, 12, 18, or 24 hours in the presence or absence of *B. subtilis* 6D1 CFE (10% vol/vol) (**C**) *B. subtilis* 6D1 CFE (20%, 10%, 5%, or 2.5% vol/vol) was applied concurrently with *S. aureus* at T0 prior to staining biofilm at 24 hours (**D**) *B. subtilis* 6D1 CFE (20%, 10%, 5%, or 2.5% vol/vol) was applied to *S. aureus* 24 hour biofilms and incubated at 37°C shaking at 100 rpm for 2 hours prior to staining residual biofilm. (**E–G**) *B. subtilis* 6D1 CFE (10% vol/vol) was applied in conjunction with 0.5 µg/mL antibiotic at T0 prior to measuring *S. aureus* biofilm growth at 24 hours; error bars represent mean ± SD in 12 independent replicates. Differences in biofilm inhibition and disruption were analyzed using a 2-tailed unpaired *t*-test (**A–D**) or one-way ANOVA, followed by Tukey’s multiple comparisons (**E–G**) where (*) *P* < 0.05; (**) *P* < 0.01; (***) *P* < 0.001.

To assess which stages of the *S. aureus* ATCC 29213 biofilm growth cycle (50) were impacted by *B. subtilis* 6D1, 10% vol/vol CFE was applied at various time points throughout biofilm growth. When CFE was applied to wells at time 0, 1, and 6 hours into the *S. aureus* biofilm growth cycle, differences in residual biofilm biomass were observed (Fig. 3A), indicating CFE is likely inhibiting *S. aureus* adherence. We hypothesized that if CFE disrupted adherence, applying CFE alongside *S. aureus* at time 0 would likely suppress biofilm formation throughout the maturation cycle. Additional experiments confirmed this hypothesis, showing that CFE applied concurrently with *S. aureus* suppressed biofilm growth, and biofilm growth did not recover within 24 hours (Fig. 3B). Together, these results suggest *B. subtilis* 6D1 produces soluble elements that inhibit biofilm adherence and early maturation—processes typically under the control of the Agr QS system (51). Furthermore, addition of *B. subtilis* 6D1 CFE reduced *S. aureus* biofilm formation (Fig. 3C) and disrupted mature biofilm (Fig. 3D) in a concentration-dependent manner. These data indicate a positive correlation between active compound abundance and antibiofilm activity.

Next, we tested whether application of *B. subtilis* 6D1 CFE improves antibiotic efficacy against biofilm growth if applied in conjunction with low doses of antibiotics. Ampicillin, gentamicin, and trimethoprim were selected for both their clinical relevance in treating Staphylococcal infections and their different antimicrobial mechanisms—ampicillin interferes with cell wall synthesis, gentamicin inhibits protein translation, and trimethoprim prevents DNA synthesis. To quantify synergistic, additive, indifferent, or antagonistic effects brought on by the addition of *B. subtilis* 6D1 CFE, minimum biofilm inhibition concentrations (MBIC) and minimum inhibitory concentrations (MIC) were determined for each antibiotic to identify the lowest permissible concentration that would allow *S. aureus* ATCC 29213 planktonic and biofilm growth. Fractional inhibitory concentration index (FICI) calculations revealed that application of 10% vol/vol *B. subtilis* 6D1 CFE in conjunction with ampicillin (FICI = 0.34 ± .08) and gentamicin (FICI = 0.28 ± .04) synergistically reduced *S. aureus* ATCC 29213 biofilm formation and possessed an additive inhibitory effect when applied in conjunction with trimethoprim (FICI = 0.87 ± .08).

To explore which CFE compounds might be driving this activity, the *B. subtilis* 6D1 genome was mined for biosynthetic gene clusters (BGCs) that might produce secondary metabolites. Seven BGCs were identified and subsequently mapped to reference BGCs identified in the undomesticated *B. subtilis* NCIB 3610 strain (GenBank Accession # ASM205596v1). Of these seven predicted BGCs, five were found to possess 100% similarity with reference clusters: bacilysin, bacillaene, bacillibactin, subtilosin A, and sporulation killing factor (Fig. S3). Interestingly, two BGCs showed 78% similarity to surfactin and 93% similarity to fengycin, non-ribosomal peptides with previously demonstrated quorum sensing interference and antibiofilm capabilities (52, 53). However, subsequent biofilm inhibition experiments using commercial fengycin obtained from *B. subtilis* revealed this compound increased biofilm formation in multiple Agr backgrounds (Fig. S4). Additional BGC mapping determined that the initially identified fengycin cluster was instead, plipistatin, a very similar lipopeptide but with a slightly different structural moiety (54). Furthermore, compared with the plipistatin reference BGC, the *B. subtilis* 6D1 BGC has lost both *ppsA* and *ppsB* non-ribosomal peptide synthetase (NRPS) genes (Fig. S3), suggesting either this metabolite is structurally distinct from plipistatin or this BGC is not efficiently transcribed (55). Compared with the surfactin reference BGC, *srfAA* and *srfAB* NRPS genes identified in *B. subtilis* 6D1 shared 75% and 77% identity, respectively (Fig. S3). *B. subtilis* 6D1 also harbors an additional ABC transporter downstream of *yciC* not identified in the reference BGC. Taken together, we conclude that *B. subtilis* 6D1 harbors biosynthetic gene clusters that closely resemble surfactin BGCs—a peptide capable of inhibiting *S. aureus* biofilm and inducing AIP secretion (53). Therefore, ethyl acetate extractions were performed to determine if these lipopeptides are produced and whether they contribute toward the antibiofilm activity observed.

### Peptides isolated from *B. subtilis* 6D1 cell-free extracts inhibit biofilm growth in all Agr backgrounds

**Fig 4:**
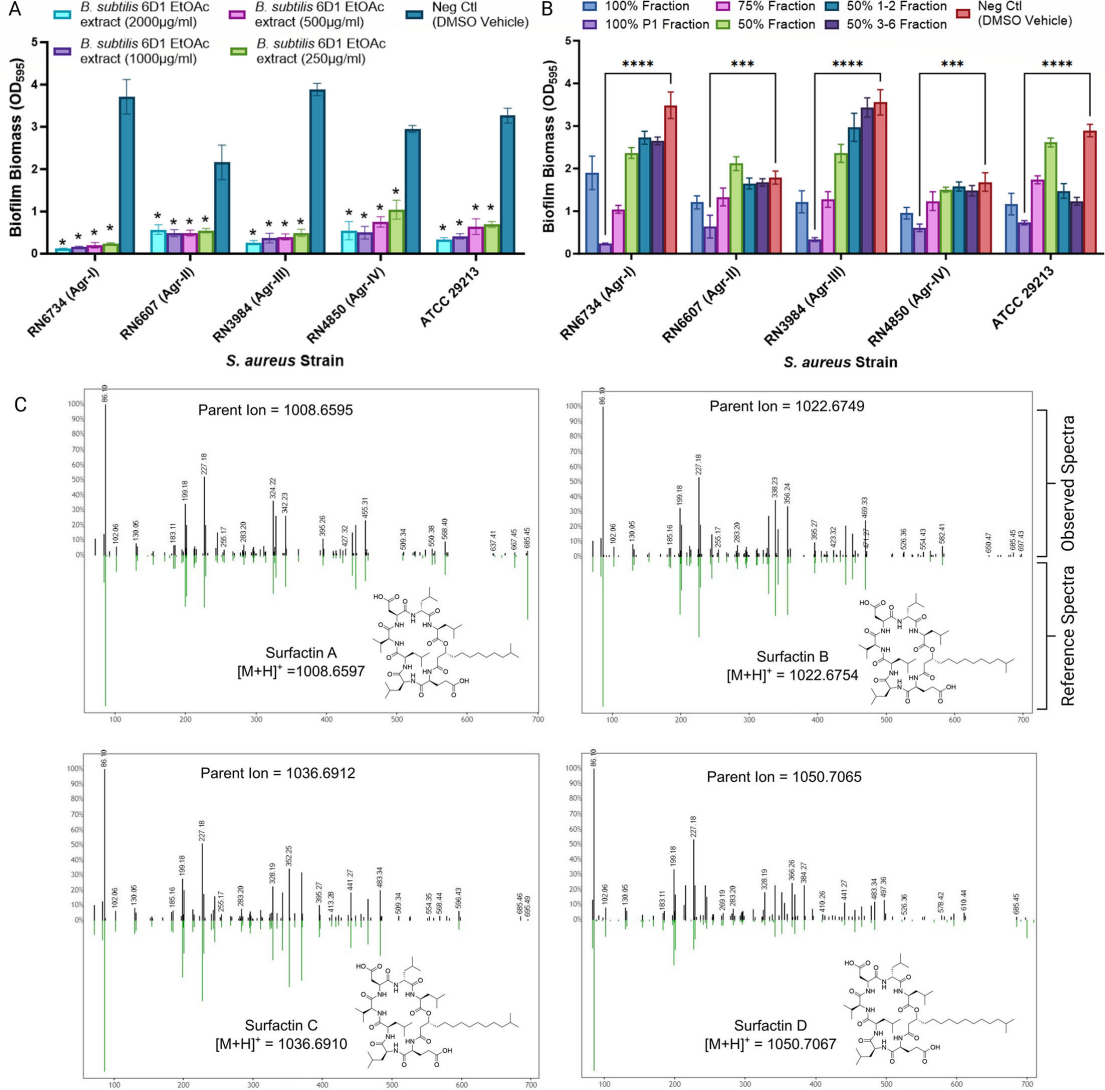
*B. subtilis* 6D1 derived peptides inhibit biofilm growth in all Agr backgrounds. (**A**) *B. subtilis* 6D1 ethyl acetate extracts exhibited dose-dependent antibiofilm activity against *S. aureus* strains with different AgrC receptors (*agr* I-IV) (*) *P*<0.0001 (**B**) Antibiofilm activity of *B. subtilis* 6D1 ethyl acetate (EtOAc) extracts (500 µg/mL) further separated by combiflash fractionation. Error bars represent mean ± SD in 12 independent replicates. Differences in biofilm inhibition were analyzed using one-way ANOVA, followed by Tukey’s multiple comparisons where (***) *P*<0.001, (****) P<0.0001 (**C**) Liquid Chromatography with tandem mass spectrometry spectra of *B. subtilis* 6D1 fraction 100%P1 m/z values that matched known molecules in the Global Natural Product Social Molecular Networking (GNPS) database. Black (observed) spectra represent peaks identified in fraction 100% P1, and green (reference) spectra represent GNPS database spectra. The x-axis represents m/z and the y-axis represents the relative abundance of the various ions.

*S. aureus* strains can harbor one of four distinct Agr QS systems, and although each Agr system possesses a unique receptor-ligand interaction, the Agr QS system in each group is responsible for regulating virulence factor gene expression and facilitating biofilm formation and dispersal (15, 56). To assess whether *B. subtilis* 6D1 peptides were driving *S. aureus* antibiofilm activity, a crude ethyl acetate extract resuspended in dimethyl sulfoxide (DMSO) was assessed for its antibiofilm activity against *S. aureus* strains possessing different Agr backgrounds. These ethyl acetate extracts obtained from crude *B. subtilis* 6D1 CFE revealed dose-dependent antibiofilm activity against all four Agr backgrounds (Fig. 4A).

*B. subtilis* 6D1 ethyl acetate extracts were separated further by flash chromatography using a stepwise gradient of water and methanol to obtain six distinct fractions with unique mass spectra. After drying, concentrated fractions were dissolved in DMSO and again evaluated for their ability to inhibit *S. aureus* biofilm formation. Compared with DMSO vehicle controls, fraction 100%P1 harbored the strongest antibiofilm activity among the six fractions (Fig. 4B). Although other fractions harbored antibiofilm activity, namely 100% and 75% fractions, 100%P1 yielded the most consistent antibiofilm activity across all Agr backgrounds. Feature-based molecular networking through the Global Natural Product Social Molecular Networking (GNPS) database confirmed the presence of surfactin A-D in fraction 100%P1 (Fig. 4C). A smaller sub-network with no known matches to GNPS database spectra was also identified in this fraction, and subsequent separation of this compound from the surfactins revealed it, too, possessed potent antibiofilm activity (Fig. S5). Both *B. subtilis* NCIB 3610 and another environmental *B. subtilis* strain, 9B5, harboring moderate antibiofilm activity were analyzed via liquid chromatography with tandem mass spectrometry (LC-MS/MS) alongside *B. subtilis* 6D1. Both 9B5 and 3610 strains produced surfactin, but neither strain possessed this unknown sub-network. Further component analysis on the 100%P1 fraction confirmed the presence of multiple surfactins as well as a unique peptide profile (Fig. S9 through S18). Collectively, we conclude that peptides produced by *B. subtilis* 6D1, namely surfactin A-D and a novel compound, prevent biofilm growth in all four *S*. *aureus* Agr backgrounds.

### *B. subtilis* 6D1 100%P1 fraction exhibits stronger antibiofilm activity compared with HPLC grade surfactin obtained from *B. subtilis*

**Fig 5:**
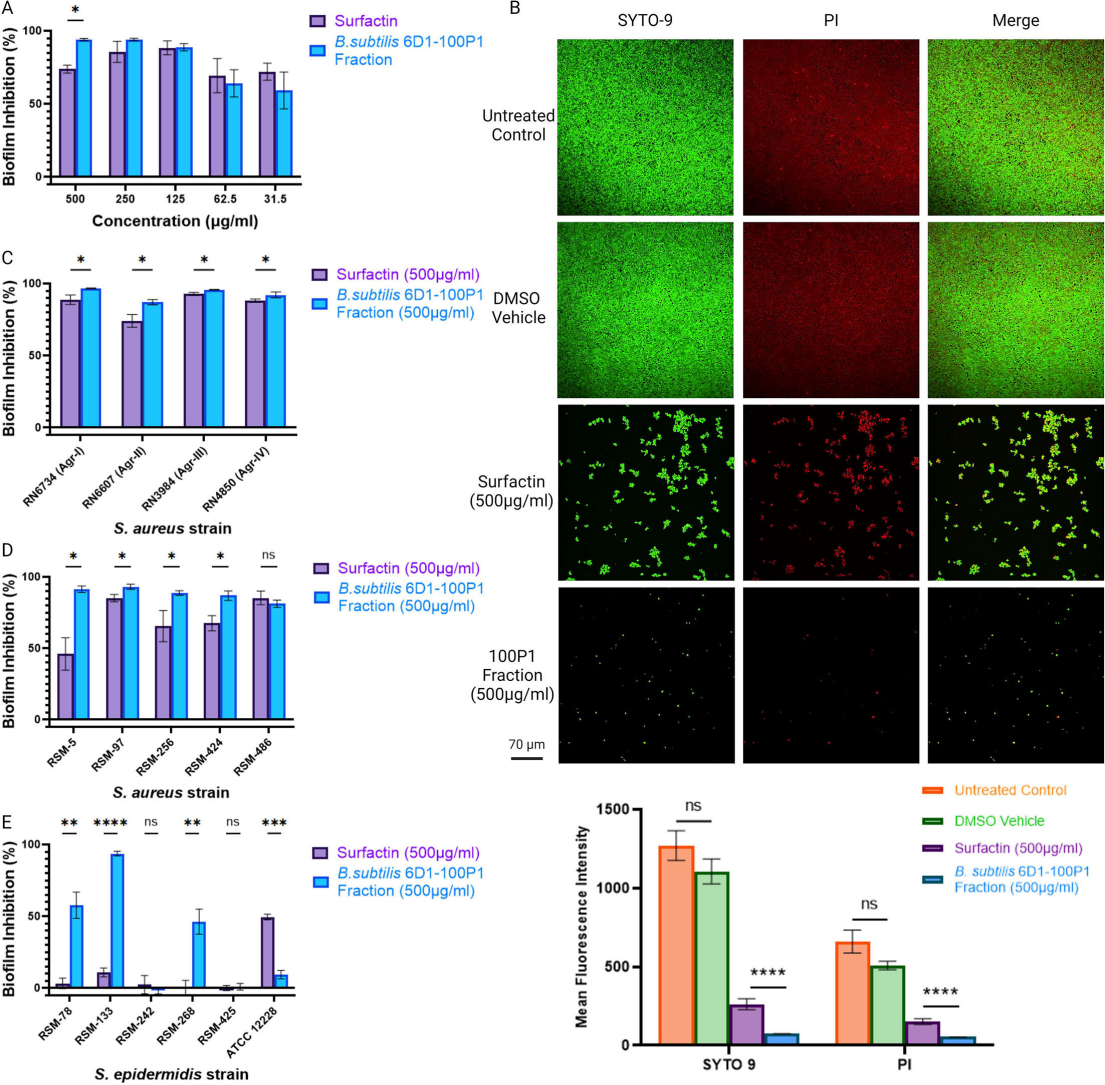
*B. subtilis* 6D1 100%P1 fraction exhibits stronger antibiofilm activity compared with commercial HPLC grade surfactin obtained from *B. subtilis*. (**A**) Antibiofilm activity of titrated concentrations of commercial surfactin and *B. subtilis* 6D1 fraction 100% P1 against *S. aureus* ATCC 29213. (**B**) Live (SYTO 9)/dead(PI) staining and confocal image analysis comparing the arithmetic mean fluorescence intensity of *S. aureus* ATCC 29213 biofilms treated with 500 µg/mL commercial surfactin and *B. subtilis* 6D1 fraction 100% P1, scale bar = 70 µm. Error bars represent mean ± SEM of 15 independent fields of view (**C**) Antibiofilm activity of commercial surfactin and *B. subtilis* 6D1 fraction 100% P1 against *S. aureus* strains with different AgrC receptors (*agr* I-IV) (**D**) Antibiofilm activity of commercial surfactin and *B. subtilis* 6D1 fraction 100% P1 against clinical *S. aureus* (**E**) and *S. epidermidis* strains. Error bars represent mean ± SD of 12 independent replicates. Differences in biofilm inhibition were analyzed using a 2-tailed unpaired *t*-test where (*) *P* < 0.05; (**) *P* < 0.01; (***) *P* < 0.001; (****) *P* < 0.0001.

A series of biofilm inhibition experiments were performed comparing the 100%P1 fraction and commercial high performance liquid chromatography (HPLC) grade surfactin produced by *B. subtilis* (Sigma-Aldrich, USA). Both 100%P1 and commercially obtained surfactin were standardized in DMSO to 10 mg/mL working stock concentrations to ensure that both identical DMSO volumes and metabolite concentrations were applied to test conditions. Additional DMSO titrations confirmed volumes used to deliver these peptides did not increase cell death compared with the untreated biofilm controls (Fig. S6). Although both commercial surfactin and fraction 100%P1 successfully inhibited *S. aureus* biofilm formation, preliminary titration experiments revealed that at 500 µg/mL, 100%P1 harbored stronger antibiofilm activity (94.1% ± 0.7%) than commercial surfactin alone (73.8% ± 2.6%) (Fig. 5A). Confocal image analysis confirmed these findings—100%P1 treated biofilms reduced SYTO 9 mean fluorescence intensity 72% more than commercial surfactin (Fig. 5B). Based on these observations, a 500 µg/mL concentration was used in all ensuing experiments. At this concentration, *B. subtilis* 6D1 fraction 100%P1 possessed stronger antibiofilm activity against all four Agr background strains with biofilm percent inhibition ranging from 88%–97% while biofilm inhibition ranged from 74%–92% for surfactin alone (Fig. 5C). This trend persisted when evaluating clinical *S. aureus* isolates. *B. subtilis* 6D1 fraction 100%P1 exhibited biofilm inhibition ranging from 82%–93% while biofilm inhibition associated with surfactin exposure was much less consistent, ranging from 46%–85% in the same isolates (Fig. 5D). Further separation of fraction 100%P1 revealed that both the surfactin mixture and the unique compound found in this fraction exhibited strong biofilm inhibition and disruption activity (Fig. S5). Therefore, it is possible this 100%P1 fraction, comprised of four surfactin isoforms and a unique compound, is either additively or synergistically increasing *S. aureus* antibiofilm activity compared with commercial surfactin.

Interestingly, differences between 100%P1 and surfactin were more apparent when evaluating biofilm inhibition activity against coagulase-negative *Staphylococcus epidermidis*, another species with a functioning Agr QS system that can form biofilm on indwelling medical devices (57). Fraction 100%P1 successfully inhibited 3/5 clinical *S. epidermidis* isolates; however, biofilm inhibition did not exceed 10% for any of the five clinical strains treated with commercial surfactin (Fig. 5E). Conversely, surfactin only inhibited biofilm formation of *S. epidermidis* ATCC 12228 while fraction 100%P1 did not have this effect. Based on these data, we conclude that at 500 µg/mL concentrations, *B. subtilis* 6D1 fraction 100%P1 comprised of surfactin A-D, and a unique compound exhibits stronger antibiofilm activity than commercial surfactin against *S. aureus* strains.

### *B. subtilis* 6D1 modulates gene expression associated with *S. aureus* quorum sensing

**Fig 6:**
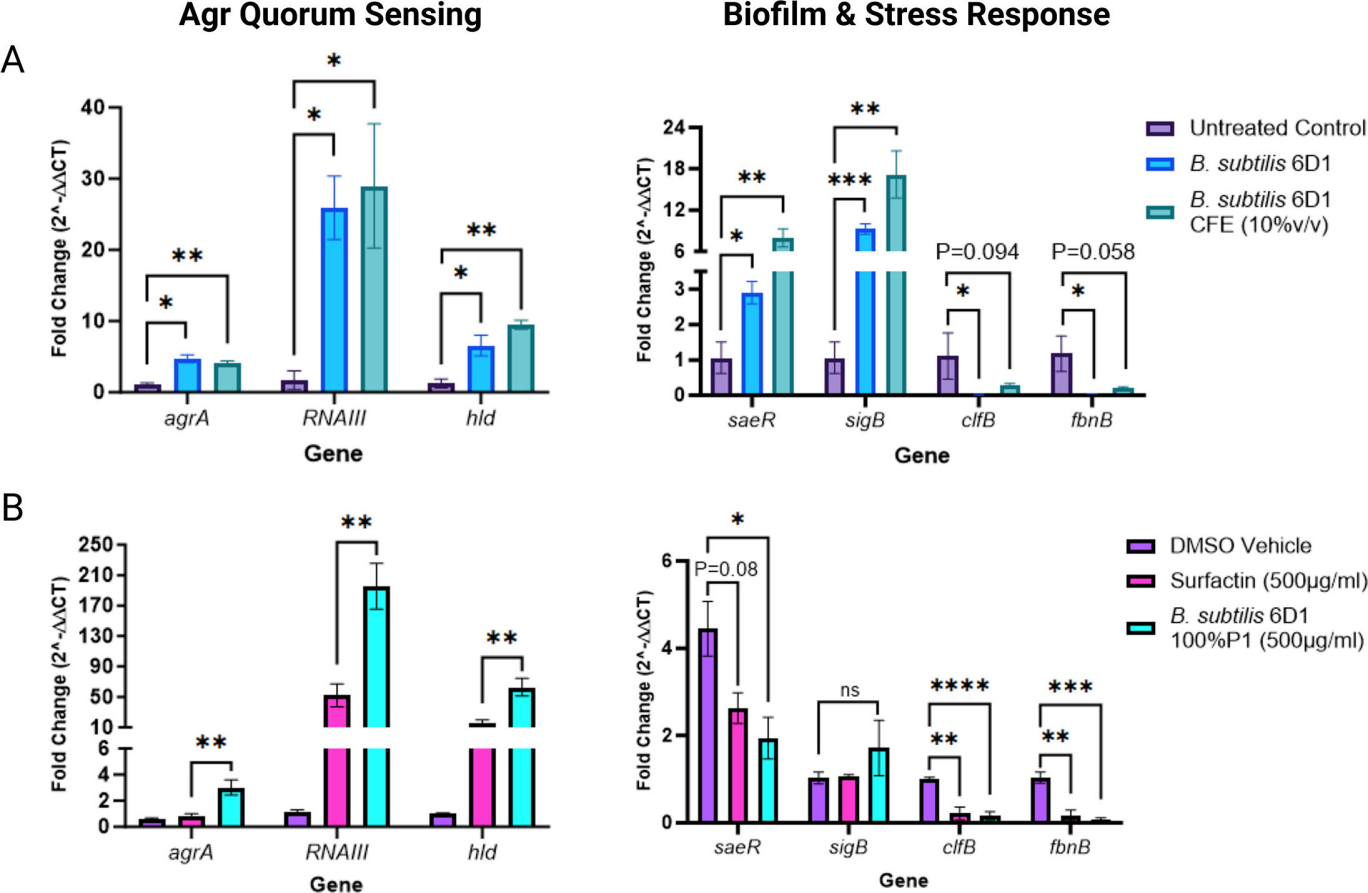
*B. subtilis* 6D1 modulates gene expression associated with *S. aureus* Agr quorum sensing and biofilm development. (**A**) *B. subtilis* 6D1 (blue) and 10% vol/vol *B. subtilis* 6D1 cell-free extract (teal) and (**B**) *B. subtilis* 6D1 fraction 100%P1 (light blue) and commercial surfactin (pink) were applied to *S. aureus* ATCC 29213 for 24 hours before RNA extraction and (left) Agr associated and (right) biofilm and stress-associated gene expression analysis via RT-qPCR. Error bars represent mean ± SD of nine independent replicates. Differential gene expression data were analyzed using one-way ANOVA followed by Tukey’s multiple comparisons using log2-transformed data where (*) *P* < 0.05; (**) *P* < 0.01; (***) *P* < 0.001 (****) *P* < 0.0001.

*S. aureus* quorum sensing involves the recognition of AIPs by their cognate AgrC receptors, which prompts the phosphorylation of AgrA and the subsequent expression of the Agr operon (24). We hypothesized that *B. subtilis* 6D1 peptides can bind AgrC and upregulate the Agr quorum sensing system in *S. aureus*. RT-qPCR experiments were conducted to investigate how *B. subtilis* 6D1, its associated cell-free extract, and the isolated 100%P1 fraction altered the expression of *S. aureus* biofilm and QS-related genes (6) (Table S2). Our data demonstrated that both 10% vol/vol CFE and *B. subtilis* 6D1 cells applied in a 1:1 ratio with *S. aureus* ATCC 29213 increased expression of *agrA*, *RNAIII*, and *hld* ranging from 4-fold to 29-fold (Fig. 6A). *AgrA*, *RNAIII*, and *hld* are all located in the *S. aureus* Agr operon, and when upregulated, transition cells from a biofilm lifestyle to a planktonic lifestyle (51). Other genes also upregulated in response to *B. subtilis* 6D1, and CFE exposure included the global stress response regulator, *sigB*, and *saeR*, the response regulator of a two-component signal transduction system that coordinates with the Agr QS system to process environmental signals and regulate the expression of numerous surface-bound and secreted proteins (58, 59). Compared with the *B. subtilis* 6D1 treatment, *B. subtilis* 6D1 CFE elicited a 9.2-fold and 6.3-fold stronger expression response for both *sigB* and *saeR*, respectively.

Based on our previous data showing an increase in antibiofilm activity of fraction 100%P1 compared with commercial surfactin, follow-up gene expression studies were conducted to determine whether these peptide mixtures elicited different gene expression responses in *S. aureus*. In line with our previous observations, gene expression analysis confirmed 100%P1 applied at 500 µg/mL increased Agr-related gene expression more than commercial surfactin applied at the same concentration (Fig. 6B Left). Despite these differences, both peptide mixtures elicited a stronger Agr-mediated gene expression response than either *B. subtilis* 6D1 or the associated CFE. Compared with the CFE treatment, application of the purified 100%P1 fraction increased *RNAIII* and *hld* expressions 166-fold and 53-fold, respectively. Interestingly, neither surfactin nor the 100%P1 fraction increased *sigB* or *saeR* expression that was observed in the *B. subtilis* 6D1 or associated CFE treatments (Fig. 6B Right). These data suggest different compounds present in the CFE are prompting the expression of multiple regulatory pathways in *S. aureus* including those responsible for Agr quorum sensing.

### *B. subtilis* 6D1 reduces *S. aureus* ATCC 29213 virulence in a human intestinal cell line

**Fig 7:**
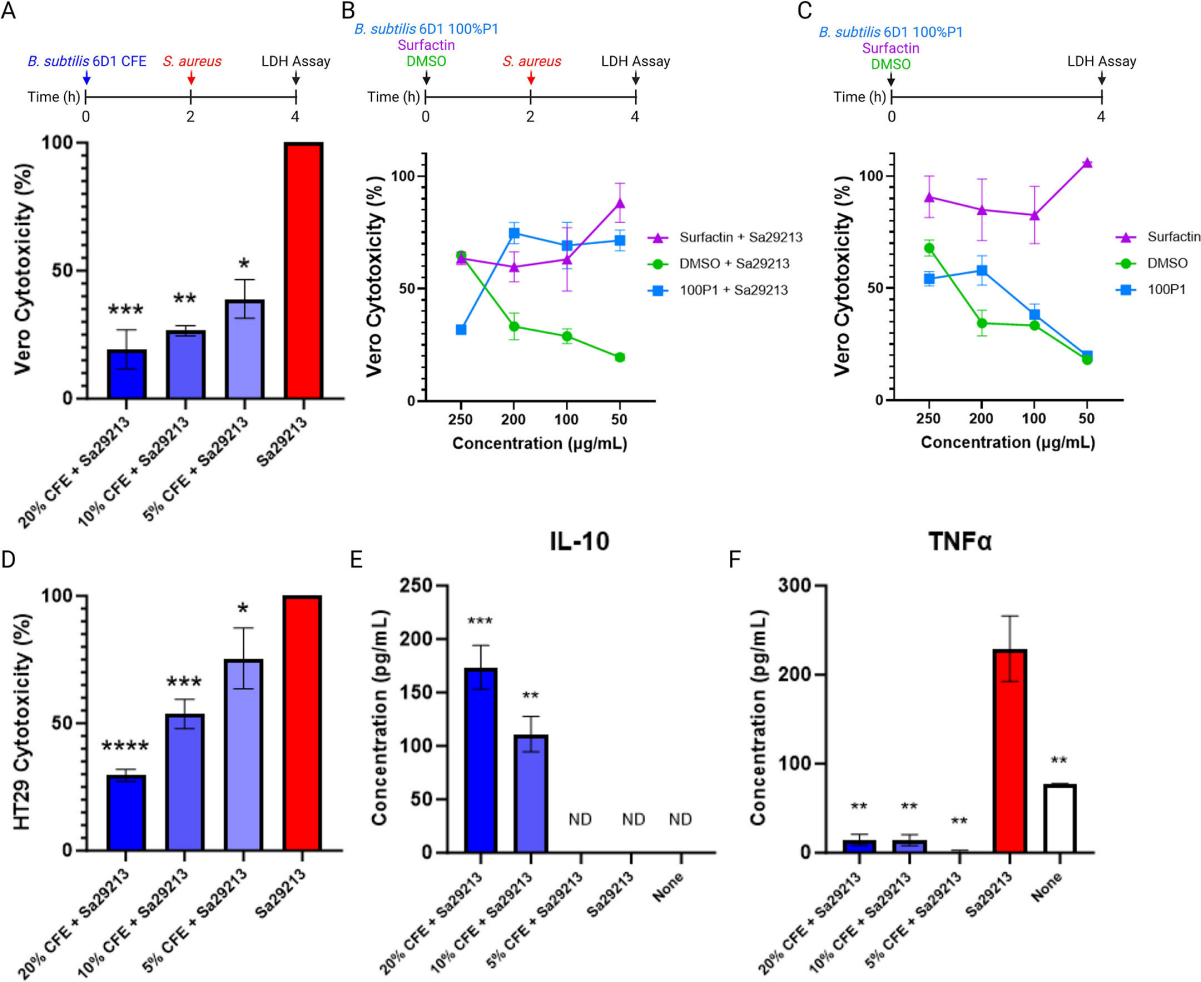
*B. subtilis* 6D1 reduces *S. aureus* ATCC 29213 virulence in a human intestinal cell line. (**A**) Vero cells pretreated for 2 hours with increasing concentrations of *B. subtilis* 6D1 CFE reduced cytotoxicity of *S. aureus* ATCC 29213 (Sa29213) induced cell death; data normalized to Sa29213 positive infection control. (**B**) Cytotoxicity of Vero cells pretreated for 2 hours with increasing concentrations of fraction 100%P1 and surfactin delivered in DMSO in the presence and (**C**) absence of a Sa29213 challenge; data normalized to LDH positive control. (**D**) HT29 cells pretreated for 2 hours with increasing concentrations of *B. subtilis* 6D1 CFE inhibit Sa29213 induced cytotoxicity; data normalized to Sa29213 positive infection control (**E**) HT29 cells pretreated for 2 hours with *B. subtilis* 6D1 CFE increase production of the anti-inflammatory cytokine IL-10 (**F**) and inhibits the production of the pro-inflammatory cytokine TNFα brought on by Sa29213 challenge. Error bars represent mean ± SD in 6 independent replicates. Vero and HT29 cell cytotoxicity results were analyzed using one-way ANOVA, followed by Tukey’s multiple comparisons relative to the positive infection control. Cytokine levels were compared using Welch’s non-parametric tests relative to the positive infection control where (*) *P*<0.05; (**) *P*<0.01; (***) *P*<0.001 (****) *P*<0.0001, ND = not detected.

It is known that staphylococcal virulence factor production and host cell invasion are mediated by Agr-dependent processes (60); hence, it is important to establish whether compounds altering *agr* expression also modify *S. aureus* virulence (61). Therefore, both Vero (CCL81) and HT29 cell lines derived from primate kidney and human gut epithelial tissues, respectively, were used to assess the cytotoxic and ameliorative effects of *B. subtilis* 6D1 CFE, fraction 100%P1, and commercial surfactin in the presence and absence of an *S. aureus* challenge. Both 100%P1 and commercially obtained surfactin were standardized in DMSO to 10 mg/mL working stock concentrations to ensure that both identical DMSO volumes and fraction dry weight/volume concentrations were applied to test conditions. At 250 µg/mL, fraction 100%P1 reduced *S. aureus*-induced cytotoxicity from 43.4% ± 12.8% to 31.8% ± 2.2%; however, this protective effect was lost at concentrations below 200 µg/mL (Fig. 7B). In the absence of an *S. aureus* challenge, fraction 100%P1 was less cytotoxic than commercial surfactin and exhibited a decreasing trend in line with the DMSO vehicle (Fig. 7C). In fact, fraction 100%P1 was significantly less cytotoxic than commercial surfactin at all concentrations except 200 µg/mL (*P* = 0.09). Interestingly, reduced DMSO concentrations also appeared to protect Vero cells, significantly inhibiting *S. aureus-*induced cytotoxicity by 24% at 50 µg/mL (Fig. 7B). This phenomenon has been observed in other cell culture studies, where low concentrations of DMSO elicit an immunomodulatory response and increased cellular activation; however, these results are not universal and appear to be cell line-specific (62, 63).

Next, we investigated whether *B. subtilis* 6D1 CFE possessed any additional protective effects beyond those observed using the refined 100%P1 fraction. Surprisingly, *B. subtilis* 6D1 CFE inhibited *S. aureus-*induced Vero cell toxicity in a dose-dependent manner (Fig. 7A). Based on these data, we sought to investigate whether the ameliorative effects observed with *B. subtilis* 6D1 CFE in Vero cells were maintained in a human intestinal cell line (HT29) and quantify specific immunological markers that may be driving this effect. Application of CFE exhibited a similar dose-dependent protective effect in HT29 cells challenged with *S. aureus* 29213 (Fig. 7D). Further investigation revealed this protective effect was assisted by the production of anti-inflammatory cytokine IL-10 and reduced production of the pro-inflammatory cytokine, TNFα (Fig. 7E and F). Together, these data demonstrate that while fraction 100%P1 exerts strong antibiofilm activity, the mixture of compounds in *B. subtilis* 6D1 CFE may possess broader beneficial properties to help combat a variety of *S. aureus* virulence strategies.

## DISCUSSION

This study successfully addressed its primary objectives by demonstrating that *B. subtilis* 6D1 antagonizes *S. aureus* through several mechanisms. These include inhibiting biofilm formation and attachment, increasing *S. aureus* sensitivity to antibiotics, and stimulating an adaptive cytokine response in human intestinal epithelial cells that helped reduce *S. aureus* cytotoxicity. These findings underscore the importance of exploring *B. subtilis* strain diversity to identify strains with unique compound signatures that antagonize *S. aureus*, reduce its virulence, and increase its antibiotic susceptibility. As a first step toward identifying novel *B. subtilis*-derived products capable of reducing virulence and improving antibiotic efficacy against multidrug-resistant *S. aureus*, it is essential to source probiotic strains from environments where frequent interactions with *S. aureus* might occur (30). In our case, 1,123 *Bacillus* strains were sourced from bovine milk and feces and a variety of environments impacted by multiple dairy operations with frequent antibiotic use comprising nearly 100,000 dairy cows in Kewaunee County, WI (64). We hypothesized that *Bacillus* strains obtained from these environments had likely evolved coercion tactics to compete with bovine *S. aureus* strains through inhibition of biofilm formation (65). In line with this hypothesis, we isolated 16 *Bacillus* strains with moderate to strong antibiofilm activity against *S. aureus*, a number that coincides with other studies deploying high-throughput screening methods to identify probiotic strains with antagonistic activity against *S. aureus* (66, 67). Further screening led to the discovery of *B. subtilis* 6D1, a strain that possessed 156 unique genes not found in other closely related strains (Fig. 1) and produced multiple peptides with antibiofilm activity including a novel compound (Fig. 4; Fig. S5). That *B. subtilis* 6D1 produced multiple surfactin isoforms coincides with another study that showed less than <0.1% of strains screened from candidate libraries were capable of producing excess surfactin (68). Together, these data suggest that purposeful probiotic screening strategies accompanied with comparative genomic analyses can be used to identify unique strains with desirable mechanisms of action. This systems approach can further help elucidate the mechanisms by which a probiotic strain performs a desired action. For instance, a variety of probiotic strains can help resolve *S. aureus* infections through bactericidal mechanisms (12, 37); however, recent evidence has demonstrated certain *Bacillus* strains can also inhibit virulence without killing these pathogenic microorganisms (69, 70). In line with these findings, application of *B. subtilis* 6D1 CFE inhibited *S. aureus* biofilm growth and eradicated mature biofilm in a dose-dependent manner (Fig. 3C and D) without inhibiting planktonic growth (Fig. 2A). Moreover*,* the ability of *B. subtilis* 6D1 to outcompete *S. aureus* ATCC 29213 in a biofilm but not in a planktonic environment suggests that the antibiofilm activity of this strain is maintained in a coculture environment (Fig. 2C). While we acknowledge these competitive benefits observed in *B. subtilis* 6D1 may be diminished or abrogated in a more complex polymicrobial environment, others report that these beneficial probiotic effects may indeed persist in the human gut and sites of *S. aureus* infection (70, 71). In fact, *B. subtilis* 6D1’s inability to inhibit *S. aureus* planktonic growth was also observed in *B. subtilis* H28, a strain reported to successfully alleviate *S. aureus*-induced mastitis infection in a murine model (70).

Subsequent gene expression assays confirmed the application of *B. subtilis* 6D1 cells, CFE, and the refined 100%P1 fraction all upregulated genes located in the *S. aureus* Agr quorum sensing operon (Fig. 6), further indicating this strain can deploy QSI-mediated antibiofilm mechanisms when in direct competition with *S. aureus*. In addition to upregulating *agrA*, increased expression of both *RNAIII* and *hld* indicate that the phosphorylation of AgrA is prompting the transcription of both the P2 and P3 promoters of the Agr regulon. *B. subtilis* 6D1 and its associated CFE including fraction 100%P1 all prevent *S. aureus* biofilm growth by upregulating genes controlled by the Agr QS system (Fig. 6). However, unlike the 100%P1 fraction, application of *B. subtilis* 6D1 and its associated CFE also increased the expression of *sigB* and *saeR* (Fig. 6A Right). Interestingly, both SigB and the SaeR/S two-component system are essential to persist intracellularly and evade the host’s innate immune system (72, 73). However, to accomplish this state of persistence, *S. aureus* also needs to silence Agr (56). The Sae-regulon also includes genes associated with biofilm formation (nucleases) and dispersal (proteases) factors. Because 100%P1 failed to upregulate *saeR* (Fig. 6B right), it is possible that different compounds produced by *B. subtilis* 6D1 not found in the 100%P1 fraction might promote expression of the Sae system independently of the Agr system and negatively impact biofilm formation by upregulating *saeR* and stimulating *S. aureus* protease production (58). In support of this hypothesis, it was recently demonstrated that cyclic dipeptides produced by *Lactobacillus reuteri* RC14 can independently downregulate the Agr and SaeR/S signaling systems and inhibit *S. aureus* virulence (74, 75).

We were surprised to observe similar *S. aureus* gene expression responses after exposure to *B. subtilis* 6D1 CFE and live cells; however, these data suggest *B. subtilis* 6D1 and its associated CFE might both inhibit virulence strategies used by pathogenic Staphylococcal species. Furthermore, our data provide additional evidence for the benefit of using a mixture of probiotic-derived compounds rather than a single compound. The synergistic effects of *B. subtilis* 6D1 CFE when applied alongside low doses of gentamicin and ampicillin (Fig. 3E and F) align with previous studies investigating the improved inhibitory effects seen when *B. subtilis* CFE was paired with gentamicin and penicillin to treat an osteomyelitis infection in mice (76). The overuse of antibiotics is a major concern in both agriculture and hospital settings, and the use of probiotic-derived compounds alongside lower doses of antibiotics may help reduce the use of antibiotics and decrease the dissemination of antibiotic-resistance genes in these environments. Furthermore, application of multiple metabolites may also prolong their utility by reducing the rate at which resistant mutants appear as is often the concern when using singular therapies (77, 78). In further support of deploying a mixture of probiotic-derived compounds, separation of *B. subtilis* 6D1 CFE into the active fraction 100%P1, comprised of surfactin A-D and an unknown compound, revealed this mixture of compounds harbored greater and broader antibiofilm activity than that of commercial surfactin also obtained from *B. subtilis* (Fig. 5A through E). To our knowledge, this is the first study demonstrating *B. subtilis*-derived peptides exhibit broad antibiofilm activity against all four *S*. *aureus* Agr backgrounds. However, despite increasing the expression of the Agr regulon, more biochemical analyses are required to confirm whether *B. subtilis* 6D1-derived peptides are directly or indirectly driving this effect. The autoinducing peptides produced by *S. aureus* all possess terminal hydrophobic residues that are predicted to create a “hydrophobic knob” that is crucial for recognition by their cognate AgrCs (Fig. S7A)(79). In comparison, the cyclic peptide of the surfactins adopts a saddle conformation in membrane-mimicking micelles (80), which brings Leu2, Leu3, and Leu6 into an arrangement similar to the “hydrophobic knob” of the AIPs (Fig. S7B). It is possible that the surfactins may mimic the AIPs and directly bind and activate AgrC. Alternatively, it may be that these peptides indirectly induce the production of *S. aureus* AIPs, which in turn bind their cognate AgrC receptors and prompt Agr upregulation. The presence of surfactin has been shown to increase AIP abundance in *S. aureus* supernatants (53); however, additional biochemical assays are needed to confirm how surfactins upregulate the Agr system. Antibiofilm activity of 100%P1 against multiple *S. epidermidis* strains was also somewhat surprising (Fig. 5E). *S. epidermidis* also harbors an Agr system that succumbs to QSI and cross-talk between similarly structured AgrD configurations (81); however, the AgrD peptide variants found in *S. epidermidis* are structurally different than those found in *S. aureus* (82). Despite this, it has been reported that peptides capable of interfering with the *S. aureus* Agr system are also capable of interfering with the *S. epidermidis* Agr systems (83). This suggests the mixture of peptides produced by *B. subtilis* 6D1 may in fact possess broader QSI activity than tested here.

Based on these peptides’ ability to decrease biofilm formation in multiple Agr backgrounds, we hypothesized that *B. subtilis* 6D1’s ability to reduce biofilm formation by upregulating Agr may inadvertently increase the expression and production of multiple virulence factors also under control of the Agr system (15). However, our cell culture data contradicted this hypothesis. The protective and immunomodulatory effects afforded by *B. subtilis* 6D1 CFE in both Vero and HT29 cells indicate the mixture of small peptides produced by this strain can reduce *S. aureus* 29213 virulence through stimulation of an intestinal adaptive cytokine response. One potential explanation for these findings centers around increased transcription of *sigB* (Fig. 6A Right). Upregulation of SigB has been shown to reduce the expression of multiple virulence factors in an effort to subvert the human immune system (73). However, agr-induced RNAIII levels are elevated in *sigB* mutants (84), implying that *sigB* expression should be reduced if *agrA* expression increases. This assumption contradicts our findings. However, since fraction 100%P1 failed to upregulate *sigB* (Fig. 6B Right), we hypothesize that this difference can be attributed to different CFE compounds independently altering *S. aureus* gene expression associated with biofilm formation and the global stress response. For instance, subtilosin A produced by *B. subtilis* KATMIRA1933 can inhibit biofilm formation in clinical *S. aureus* strains and disrupt quorum sensing in *Listeria monocytogenes* and *Salmonella enterica* (69, 85, 86). A biosynthetic gene cluster for subtilosin A was also identified in the *B. subtilis* 6D1 genome with high sequence similarity to the reference cluster (Fig. S3). Therefore, it is possible subtilosin A may be contributing to the QSI or anti-virulence activity observed in experiments where crude CFE was employed. Based on these data, it is tempting to speculate that combative strategies employed by probiotic bacteria might also inadvertently reduce the risk of chronic *S. aureus* infections by upregulating genes typically repressed during chronic disease states (20, 21). Interestingly, HT29 monolayers treated with 20% vol/vol CFE harbored less LDH accumulation than untreated monolayers not challenged with *S. aureus*, suggesting this consortium may improve gut epithelial integrity in unchallenged cell types. HT29 cells have been frequently used to study the intestinal immune response to bacterial infection, adhesion, and survival (87), and although they represent a valuable model due to their similarities with enterocytes of the small intestine, their limitations and the relevance to the *in vivo* situation are still under debate. Nevertheless, our results align with previous research demonstrating how probiotic derived small molecules, termed postbiotics, can improve gut physiological processes and adaptive immunity (88, 89).

In addition to exhibiting stronger antibiofilm activity, our cell culture results also demonstrated the 100%P1 fraction was less cytotoxic than commercial surfactin (Fig. 7C) and decreased *S. aureus-*induced cytotoxicity in Vero cells when applied at 250 µg/mL (Fig. 7B). Work by Lee and colleagues demonstrated that each surfactin isoform (A-D) reduces macrophage cell viability at high concentrations but protects macrophage cells against an LPS challenge at lower concentrations (68). These data suggest that commercial surfactin may have been applied at too high a concentration to observe any protective benefits in Vero cells infected with *S. aureus*. It is also possible that differences between the commercial surfactin product and fraction 100% P1 are due to the combination and varying concentrations of multiple surfactin isoforms in fraction 100%P1 compared with the commercial surfactin that is enriched with surfactin C (Fig. S9). Alternatively, this activity may be due to the presence of the uncharacterized peptide found in 100%P1, since refined fractions enriched with this peptide also inhibited *S. aureus* biofilm growth and disassembled mature biofilm (Fig. S5 and S9). To purify this peptide and elucidate its structure, batch-culturing was used to increase the abundance of this unknown peptide; however, the resulting concentrations were too low to deduce an accurate molecular formula or structure. We further attempted to enrich this unknown compound by knocking out the *B. subtilis* 6D1 surfactin gene cluster and obtaining new extracts without any surfactins. However, attempts to knock out the surfactin gene cluster using natural transformation, SPP1 phage transduction, and transposon insertion mutagenesis (90) were all unsuccessful. Further genomic analysis revealed that *B. subtilis* 6D1 has an endogenous pBS32-like plasmid (Fig. 1B) known to encode proteins that inhibit natural competence and DNA uptake (46, 91). Therefore, it is possible that this strain possesses an impaired competence machinery that inhibited the uptake of foreign DNA in our experiments.

### Limitations of the study

This study presents a comprehensive investigation into the potential of *B. subtilis*-derived compounds, particularly from strain 6D1, to mitigate *S. aureus* biofilm formation and virulence. However, there are several key limitations. First, while our screening approach yielded promising *Bacillus* strains with antibiofilm activity, our study’s reliance on a single geographic location may limit the generalizability of our results to other ecosystems and microbial interactions. Second, while *B. subtilis* 6D1 and its secreted compounds exhibited potent antibiofilm activity and modulation of *S. aureus* gene expression, the exact mechanisms underlying its efficacy remain to be fully elucidated and require further biochemical analyses. For example, while we characterized multiple surfactin isoforms in fraction 100%P1, it remains unknown what the binding affinity and specificity of individual surfactin isoforms to the AgrC receptor are and how these peptides compete for AgrC binding sites with self-secreted AIPs produced by *S. aureus*. Future studies using purified *B. subtilis* 6D1 peptides rather than refined fractions like 100%P1 will help us to understand the binding affinity and specificity of these compounds to a variety of AgrC receptors found in multiple Staphylococcal Agr signaling systems (81, 82, 92). Additionally, technical challenges hindered the purification and structural characterization of the potential novel compound identified in our mass spectra chromatograms and molecular networking analyses (Fig. S9). Despite multiple approaches and numerous failed attempts to knock out the surfactin gene cluster in *B. subtilis* 6D1, future genomic engineering of this strain may require CRISPR-based gene editing to remove the surfactin gene cluster and assist in the purification of this novel compound.

Another limitation of our study is the perceived paradox between Agr activation and reduced toxicity in the cell culture assays. While the number and diversity of compounds present in the CFE may have upregulated signaling cascades (SigB and Sae) known to, in some cases, restrict virulence factor production and stability (73, 93), it is more likely that the 2 hour pre-treatment of cells with *B. subtilis* 6D1 CFE prior to *S. aureus* exposure stimulated a robust cytokine response that was able to ameliorate virulence associated with Agr upregulation brought on by the CFE. However, because we did not quantify virulence factor production directly, we can only speculate that these virulence factors, if produced, were insufficiently abundant or active at the time of collection to overshadow the improved cytokine response afforded HT29 cells pre-treated with *B. subtilis* 6D1 CFE. While our *in vitro* assays demonstrate the potential therapeutic benefits of *B. subtilis* 6D1 and its associated compounds, *in vivo* studies are essential to validate these findings and assess their clinical relevance more thoroughly. Future studies utilizing animal infection models (94–98) will be crucial to assess the therapeutic feasibility and safety profile of *B. subtilis* 6D1 and its associated compounds. These studies will also help assess the utility of *B. subtilis* 6D1 in complex and highly variable communities seen in the gastrointestinal tract and sites of *S. aureus* infection.

Despite these challenges and limitations, our work demonstrates that *B. subtilis* 6D1 may serve multiple roles in preventing *S. aureus* virulence by inhibiting biofilm formation and attachment, increasing sensitivity to antibiotics, and stimulating an adaptive cytokine response capable of reducing *S. aureus* cytotoxicity and potentially improving intestinal health. Collectively, our work illustrates the importance of exploring the genomic diversity present within a single relatively well-characterized bacterial species like *B. subtilis* and emphasizes how this exploration can lead to the identification of unique probiotic strains capable of producing novel molecules with potential therapeutic value. Given this species diversity, a thorough understanding of probiotic strain mechanisms, like those described for *B. subtilis* 6D1, is essential if they are to serve as preventive and/or therapeutic options against biofilm-forming multidrug-resistant pathogens like *S. aureus*.

## MATERIALS AND METHODS

### Strains and chemicals used in this study

Please see Table S1 for a list of strains used in this study. Surfactin from *B. subtilis* > 98% (CAS: 24730–31-2) (Lot:0000134293) (Source:0000128758) and fengycin from *B. subtilis* ≥ 90% (CAS: 102577–03-7) (Source: SLCK5954) were purchased from Sigma-Aldrich, USA. Lysostaphin (CAS:9011–93-2) (Lot:109M4010V) used to extract RNA from *S. aureus* was also purchased from Sigma-Aldrich, USA. Dimethyl Sulfoxide (CAS:67–68-5) (Lot: 184176) used to deliver peptides and as a comparable vehicle control was purchased from Fisher Chemical, USA.

### *Bacillus* candidate screening

To attain presumptive *Bacillus* isolates, 50 environmental samples comprised of bovine feces and milk, and manure-contaminated river sediment and soil were collected across Kewaunee County, Wisconsin—home to 16 concentrated animal feeding operations and more than 200 smaller dairy farms. One gram per sample was applied to 9 mL 0.1% peptone, heated at 72°C for 20 minutes to induce *Bacillus* sporulation, and plated aerobically on trypticase soy agar (TSA) plates. To ensure only a single strain was obtained, a total of 1,123 presumptive *Bacillus* colonies were individually picked with sterile toothpicks and inoculated into fresh square TSA plates in a 6 × 6 grid. To reduce overgrowth from any fastidious strains, plates were grown at room temperature for 48 hours. After incubation, each strain was sub-cultured on agar plates to ensure pure culture isolation and subsequently in 500 µL trypticase soy broth (TSB) in 96-well deep v-well reservoirs (Thermo Scientific, USA). All cultures were grown for 24 hours at 37°C and used immediately for antimicrobial screening assays.

All *Bacillus* strains were assessed for antagonistic activity against methicillin-resistant and methicillin-susceptible clinical and type strains of *S. aureus* using drop diffusion overlay and cross streak assays (99). For drop diffusion assays, all 1,123 presumptive *Bacillus* isolates were grown for 24 hours in TSB at 37°C, and 5 µL was spotted onto square petri plates (VWR International, USA) with TSA media in a 6 × 6 grid fashion and allowed to dry. Seven milliliters of soft TSA (0.75% agar) harboring 150 µL of exponential phase *S. aureus* cultures (~2.5 x 10^8^ CFU/mL) were overlaid onto the TSA plate and allowed to dry. Plates were then inverted and incubated at 37°C for 18 hours before measuring clearing zones indicative of antagonistic activity. A total of 75 strains exhibiting inhibition against both *S. aureus* strains were subsequently assayed using cross-streak methods. Briefly, 100 µL of each presumptive *Bacillus* culture was inoculated into 10 mL TSB and incubated overnight at 37°C. A loop full of each culture was struck vertically down the center of a square TSA plate and incubated for an additional 16 hours at 37°C. The next day, nine different methicillin-susceptible and methicillin-resistant clinical and type strains, including the two used previously for drop diffusion assays, were struck perpendicularly towards the *Bacillus* streak, allowed to dry, and incubated at 37°C overnight prior to measuring zones of inhibition. An additional library of *Staphylococcus* strains isolated from the milk of 27 different dairy cows diagnosed with mastitis was used to assess the antagonistic activity of candidate *Bacillus* strains.

*Bacillus* strains (*n* = 46) exhibiting broad antagonistic activity against *S. aureus* in both assays were individually cultured in 125 mL baffled shake flasks and incubated at 37°C shaking at 230 RPM for 48 hours prior to harvesting cell-free extracts for use in subsequent biofilm and planktonic inhibition assays. After incubation, cultures were centrifuged at 4°C at 10,500 × *g* for 20 minutes. Cell-free extract (CFE) was harvested and filter-sterilized using a 0.22 µm surfactant-free cellulose acetate filter (Corning, USA). CFEs were flash frozen in liquid nitrogen and stored at −80°C for future use. A total of 39 clinical methicillin-susceptible and methicillin-resistant *S. aureus* strains were used to assess the antibiofilm activity of *Bacillus* CFEs. A list of all Staphylococcal strains used throughout this work is outlined in Table S1. *Bacillus* strains (*n* = 16) exhibiting broad antimicrobial potential in a planktonic or biofilm environment were assayed for relevant pathogenic *Bacillus* toxin genes (43) prior to sequencing the 16S rRNA gene region for taxonomic classification. Among these 16 candidates, a single strain, *B. subtilis* 6D1, exhibited stronger antibiofilm activity without affecting planktonic growth and thus was selected for more detailed investigations.

### Whole genome sequencing, phylogenomic analysis, and pangenome analysis

Whole genome sequencing and assembly were performed on *B. subtilis* 6D1 to conduct comparative genomic studies and mine for potentially unique genetic traits. Briefly, *Bacillus* DNA was isolated using a DNeasy Blood and Tissue Kit (Qiagen, Germany) according to the manufacturer’s instructions. To improve sequencing accuracy at the nucleotide level, both Illumina (400Mbs) and Nanopore (300Mbs) sequencing was performed at SeqCenter (Pittsburgh, USA). Quality control and adapter trimming were performed with bcl2fastq (100) and porechop (101) for Illumina and Nanopore sequencing, respectively. Hybrid read assembly was performed *de novo* with Unicycler (102), and assembly statistics were recorded with QUAST (103). The taxonomy classification of the *B. subtilis* 6D1 genome assembly was confirmed using GTDB-tk (Gene Bank Accession # CP129123-CP129124). Assemblies were annotated with Prokka (104), CARD (105), Phigaro (106), and VirSorter2 (107). Genome maps were created using Proksee (104). Assemblies were also analyzed in AntiSmash 6.0 to search for unique biosynthetic gene clusters (BGCs) (108). Unique BGCs were further compared with reference BGCs using Clinker (109). Phylogenetic analysis comparing *B. subtilis* 6D1 with 98 similarly identified Refseq genomes using 500 random single copy protein-coding genes was performed using Minhash similar genome finder and a codon tree builder pipeline using a randomized axelerated maximum likelihood (RAxML) phylogeny model found in the Bacterial and Viral Bioinformatics Resource Center (BV-BRC) (110).

All available genomes of *B. subtilis* on the Integrated Microbial Genomes (IMG) database were downloaded, and phylogenomic analysis was performed using Anvi’o 6.2 (111). Specifically, we annotated the single copy core genes in all the genomes, concatenated a set of 71 core genes and aligned them in Anvi’o. The phylogenomic tree was constructed according to an approximately-maximum-likelihood method as implemented in FastTree 2.1.10 (112) using the Jones-Taylor-Thorton model. The phylogenomic tree was edited using iTOL (113). Pangenome analyses utilized only genomes that were most closely related to *B. subtilis* 6D1 in the phylogenomic tree (purple clade in Fig. 1C). Pangenome analysis was performed in Anvi’o as follows: a genome storage database was generated comprised of the *B. subtilis* 6D1 assembly and each of the closely related genomes; the pangenome analysis was run using the following parameters: minbit = 05 and a mcl-inflation = 0.9 for the clustering. Diamond was used to compare the genes in sensitive mode (114). The pangenome was visualized, inspected, and edited in Anvi’o. Annotation of unique *B. subtilis* 6D1 genes was performed in EggNOG-mapper v2.1.12.

### Competition assays

For planktonic competitions, 250 µL (5 × 10^7^ CFU) of exponential phase *B. subtilis* 6D1 and *S. aureus* ATCC 29213 were added to 25 mL TSB in a baffled shake flask and incubated at 37°C shaking at 200 RPM. Biofilm competitions utilized 7 mm polystyrene beads (Polysciences, Inc, Warrington, PA) as a surface for cell attachment, biofilm growth, and biofilm dispersal (115). For biofilm competitions, equal concentrations of *B. subtilis* 6D1 and *S. aureus* populations (~1 × 10^5^–1 × 10^6^) were added to a glass test tube containing 5 mL of 1.5% TSB + 0.3% glucose media (116) and a single polystyrene bead (117, 118). After 24 hours of incubation at 37°C in a roller drum, the bead was placed in 1 mL phosphate-buffered saline (PBS) and sonicated with a handheld Qsonica model CL-188 instrument (QSonica, USA) for 10 seconds at 60 Hz to remove attached cells. Detached cells were serially diluted and plated on TSA for CFU enumeration. For all competitions, CFU counts were obtained for both bacterial species at time point 0 and after 24 hours of incubation. Strain fitness and selection rate were calculated as previously described by Travisano and Lenski (119).

### Biofilm inhibition and disruption assays

*Bacillus* CFEs were applied at varying concentrations to Nunc treated 96-well microplates (Thermo Scientific, USA) containing 1.5% TSB + 0.3% glucose media (116). Test wells were seeded with 5 × 10^5^ CFU/mL of *S. aureus* and incubated statically at 37°C. After 24 hours, media was removed, and biofilms were gently washed three times with PBS, fixed for 20 minutes at 55°C, and stained with 0.1% crystal violet (CV) for 15 minutes. After 15 minutes, CV was removed, and wells were washed twice with PBS and allowed to dry. CV was solubilized with 30% acetic acid. Biofilm inhibition was measured spectrophotometrically relative to untreated control wells at OD_595_. To assess biofilm disruption, *Bacillus* CFE (2.5%–20% vol/vol) was mixed with PBS and applied to *S. aureus* 24-hour mature biofilms and incubated at 37°C shaking at 100 RPM for 2 hours. To account for any mechanical disruption not driven by CFE, biofilms treated only with PBS were used as a negative control. Residual biofilms were fixed for 20 minutes at 55°C and quantified by crystal violet staining as outlined above. Assays were performed in quadruplicate and averaged across three independent experiments. Biofilm inhibition and removal were confirmed via CFU plating after the initial PBS wash but prior to any CV staining.

### Antibiotic synergy biofilm inhibition assays

*Bacillus* CFEs exhibiting antibiofilm potential were applied in conjunction with ampicillin, gentamicin, and trimethoprim. Antibiotic concentrations were based on preliminary biofilm and planktonic minimum inhibitory concentrations and recommended CLSI breakpoints (120). Checkerboard assays and fractional inhibitory concentration indices were used to assess whether coadministration of antibiotic and *Bacillus* CFE was synergistic(≤0.5), additive (0.5–1.0), or indifferent (1–4)(99). Briefly, 5 × 10^5^ CFU/mL *S*. *aureus* ATCC 29213 was inoculated in polystyrene 96-well plates and treated with either 10% vol/vol *Bacillus* CFE, antibiotic (either gentamicin, ampicillin, or trimethoprim) or a combined antibiotic + *Bacillus* CFE treatment. Each antibiotic or antibiotic + *Bacillus* CFE combination was added at time point 0, and biofilm growth was measured via CV staining after 24 hours at 37°C incubation.

### Gene expression assays

RNA was isolated from 24-hour broth cultures seeded with 5 × 10^5^ CFU/mL *S*. *aureus* ATCC 29213 co-inoculated with either *B. subtilis* 6D1 cell-free extract (10% v/v), 5 × 10^5^ CFU/mL *B. subtilis* 6D1 cells (1:1), commercial grade surfactin (500 µg/mL), or *B. subtilis* 6D1 100%P1 fraction (500 µg/mL) using a Qiagen RNeasy Mini kit adapted to include a modified lysis step with lysostaphin (121). Prior to RNA extraction, each treated culture was mixed 1:2 with RNA Protect (Qiagen, Germany), vortexed for 10 seconds, and incubated at ambient temperature for 10 minutes. In total, 200 µL of lysis buffer (30 mM Tris, 1 mM EDTA, 20 mg/mL lysozyme, 100 µg/mL lysostaphin, pH = 8) and 20 µL proteinase K were applied to each culture and incubated at ambient temperature on a rotary shaker (Fisher Scientific, USA) for 45 minutes. RNA quality and quantity were determined with a Qubit spectrophotometer (Thermo Scientific, USA). cDNA was obtained using the Qiagen Quantitect reverse transcriptase kit according to the manufacturer’s instructions (Qiagen, Germany). qPCR conditions were optimized and evaluated on a BioRad CFX96 real-time instrument (Bio-Rad Laboratories, USA) based on previous work (6). RT-qPCR reaction volumes (20 µL) were comprised of 10 ng cDNA, 300 nM forward and reverse primers, and SYBR green master mix (Bio-Rad Laboratories, USA). The real-time cycling conditions were as follows: 95°C for 10 minutes, followed by 40 cycles of 95°C for 15 seconds and between 58.5°C and 60°C (based on primer melting temperature) for 1 minute. Gene expression was quantified relative to the *rpoB* gene (6, 83) and calculated using the Livak method (122). RNA expression was determined using three independent bacterial cultures tested in triplicate. Gene primers and their associated melting temperatures are listed in Table S2.

### Confocal laser scanning microscopy

To assess the impact of *B. subtilis* 6D1 compounds on *S. aureus* biofilm formation, ibidi 8-μwell coated plates (ibidi, Germany) containing 400 µL 1.5% TSB + 0.3% glucose were used to grow and subsequently stain residual biofilms. Bacterial viability staining was performed using the Live/Dead BacLight Bacterial Viability Kit (Thermo Scientific, USA) according to manufacturer’s instructions. Briefly, both SYTO 9 and propidium iodide (PI) stains were thawed away from light and mixed with sterile distilled water at a final concentration of 0.1% for each dye. This mixture was equilibrated at ambient temperature for 10 minutes, and 200 µL of the resulting mixture was applied to each well and incubated at ambient temperature for 15 minutes in the dark. Prior to imaging, the stain solution was removed, and biofilms were washed twice with sterile PBS. Biofilms were immediately observed using a 40× objective and measured via confocal laser scanning microscopy (CLSM) on a Nikon Eclipse Ti instrument (Nikon, Japan). Five pre-determined fields of view were imaged per well on three independent wells per biofilm culture condition. Images and mean fluorescence intensity were analyzed in Imaris (version 9.9) as previously described (123).

### *Bacillus* CFE preparation for LC-MS/MS analysis

To extract and separate lipopeptides from crude *Bacillus* CFE, liquid-liquid extractions using ethyl acetate were performed as previously described (124–126). Briefly, *Bacillus* strains were cultured in 500 mL TSB in 2L baffled shake flasks for 48 hours and centrifuged (4,300 RPM, 30 minutes) at 4°C. Three liters of culture supernatant were mixed in a 1:1 vol ratio (vol/vol) with ethyl acetate and allowed to sit overnight with occasional mixing. Ethyl acetate fractions were collected using a separatory funnel and dried under vacuum in a Rotavapor rotary evaporator (Buchi AG, Switzerland) at 32°C. Metabolites were resuspended in DMSO and filtered through a 0.45 µm Durapore filter (Millipore, USA). Extracts were reassessed for antibiofilm activity prior to LC-MS/MS analysis. Briefly, 100 µL solubilized ethyl acetate extracts were mixed with 15 µL of 2% acetonitrile and 0.1% formic acid prior to 10 µL injections on a 25cm × 75 µm column. MS/MS data were collected on charge states 1–6 using an Orbitrap analyzer.

### Fractionation of ethyl acetate organic and 100%P1 extracts

To simplify the ethyl acetate extract, flash chromatography was performed on a Teledyne Isco CombiFlash NextGen 100, fitted with a C18 column as previously described (124). Briefly, the extract was dissolved in 25% methanol and injected into the column. A stepwise gradient of water (A) and methanol (B) from 15%B, 25%B, 50%B, 75%B, to 100%B was used to separate the extract. Fractions were collected using the automatic fraction collector. During the stepwise gradient, individual peaks were collected separately from the rest of the flow through to provide a fraction enriched with those compounds. These peaks were labeled as X%P1 for the first peak separated in any given fraction. Divided fractions were combined and dried *in vacuo* using a rotary evaporator. To further separate 100%P1, this fraction (221 mg) was separated on a reversed-phase open column (RediSep Rf C18 50 g; 60% to 100% aq. methanol) to generate two subfractions containing only surfactins (100%P1-A) or only the unique compound (100%P1-B). Fraction 100%P1-B (142 mg) was purified using a Dionex UltiMate 3000 HPLC system, using a Luna C18 column (250 mm × 15 mm, Phenomenex), running acetonitrile with 0.1% formic acid and H_2_O with 0.1% formic acid as the mobile phase.

### LC-MS/MS analysis

Fractions and isolated peaks were dried *in vacuo*, resuspended in 50% methanol, and analyzed on a Q-Exactive quadrupole orbitrap mass spectrometer (Thermo Scientific, USA) coupled to a Dionex ultra-performance liquid chromatography (UPLC) system (Thermo Scientific, USA). The UPLC method was 5% methanol for 0.5 minutes followed by a linear gradient from 5% methanol to 97% methanol over 16 minutes. The 97% methanol wash was held for 2 minutes before switching back to 5% methanol over 0.5 minutes and re-equilibrating at 5% methanol for 1 minute. The UPLC flow rate was 0.35 mL/min over a Phenomenex Kinetex XB-C18 chromatography column with dimensions 2.1 × 100 mm and particle size 2.6 µm. The mass spectrometer scanned from 200 to 2000 m/z in positive mode, and ion fragmentation was achieved using stepped normalized collision energy of 30%, 35%, and 40%. The data were collected in profile mode and manually inspected and filtered using MzMINE2 (127). MzMINE2 was used to create an aligned feature list and quantification table of the mass spectral data of the biologically active peak. These files were used to perform feature-based molecular networking through GNPS (128). The GNPS spectral libraries were searched for matches to submitted MS/MS spectra with a cosine score threshold of 0.7 and a minimum of 6 matched peaks to the library spectra. The resulting network file was visualized and analyzed using Cytoscape v3.8.0.

### Cytokine and cytotoxicity assays

Gibco Dulbecco’s Modified Eagle Medium (DMEM) (Thermo Scientific, USA) consists of 10% fetal bovine serum and 0.1% Gibco Antibiotic-Antimycotic (Thermo Scientific, USA). Cells were cultured for 7 days at 37°C in a carbogen (95% O_2_, 5% CO_2_) atmosphere, with the culture medium being changed every 2 to 3 days until cells were confluent. For CCL81 cell lines, approximately 2  ×  10^4^ cells were seeded into new 96 well plates (Corning, USA) and allowed to form monolayers for 48  hours prior to cytotoxicity testing. For HT29 cells, approximately 2  ×  10^4^ cells were seeded into new 24-well plates (Corning, USA) and allowed to differentiate for 33 days prior to treatment. After removing media, cells were washed twice with Gibco PBS, pH 7.4 (Thermo Scientific, USA) and equilibrated as previously described before adding bacteria (129, 130). Vero cells were equilibrated in EC buffer (135 mM NaCl, 15 mM HEPES, 1 mM MgCl_2_, 1 mM CaCl_2_) for 4 hours before adding bacteria. HT29 cells were equilibrated in DMEM without antibiotic for 4 hours prior to adding bacteria. Exponential phase *S. aureus* ATCC 29213 cultures grown in 1.5% TSB + 0.3% glucose media were standardized to OD_600_ 1.0, centrifuged, washed with DPBS, and applied to appropriate test wells at a final concentration of ~6 × 10^7^ CFU/well. After treatment, cells were centrifuged at 600 g for 5 minutes. Cytotoxicity was determined using a lactate dehydrogenase (LDH) assay kit according to the manufacturer’s protocol (Abcam, UK). Using HT29 cells, the human pro-inflammatory TNF-α and the anti-inflammatory IL-10 ELISAs were performed using Quantikine Colorimetric Sandwich ELISA Kits (R&D Systems, USA) according to the manufacturer’s protocol. All treatments were performed in triplicate and assayed in duplicate.

### Statistical analysis

Statistical analyses were performed using GraphPad Prism 10.1.2 (San Diego, CA). Differences where *P* < 0.05 were considered significant. Differences in biofilm inhibition and disruption were analyzed using a 2-tailed unpaired *t*-test (two groups), or one-way ANOVA, followed by Tukey’s multiple comparisons (≥3 groups) (6, 131). Differential gene expression was analyzed using one-way ANOVA, followed by Tukey’s multiple comparisons using log2-transformed data (6, 122). Vero and HT29 cell cytotoxicity results were analyzed using one-way ANOVA, followed by Tukey’s multiple comparisons (132, 133). Cytokine levels were compared between the differently treated cell lines using Welch’s non-parametric tests (134).

## Data Availability

Genome assemblies were deposited in NCBI under BioProject accession number PRJNA988465. All LC-MS/MS data were deposited in the Center for Computational Mass Spectrometry’s MassIVE database under project number MSV000092675.

## SUPPLEMENTAL MATERIAL

Supplemental Text and Figures msystems.00712-24-s0001.pdf Supplemental methods and Figures S1-S17.

Supplemental Tables msystems.00712-24-s0002.docx Tables S1-S4.

ASM does not own the copyrights to Supplemental Material that may be linked to, or accessed through, an article. The authors have granted ASM a non-exclusive, world-wide license to publish the Supplemental Material files. Please contact the corresponding author directly for reuse.
