# Supplementary material for: Bacillus subtilis-derived peptides disrupt quorum sensing and biofilm assembly in multidrug-resistant Staphylococcus aureus
Source: mSystems. 2024 Jul 11;9(8):e00712-24. doi: 10.1128/msystems.00712-24 (PMC11334493; doi:10.1128/msystems.00712-24)
Supplement: Supplemental Text and Figures — Supplemental methods and Figures S1-S17. [file msystems.00712-24-s0001.pdf]

Supplemental Methods

Isolation of Surfactins from 100%P1 fraction

The 100%P1 fraction that has anti-biofilm activity was applied to a 50 g reverse-phase CombiFlash ISCO (RediSep Rf C18, Teledyne) eluted with a linear gradient system (90%→ 100% aqueous MeOH), which yielded two subfractions (100%P1-Sub1 and 100%P1-Sub2). Fraction 100%P1-Sub2 (134 mg) was purified using the Dionex UltiMate 3000 HPLC system, coupled with a Phenomenex Luna C18(1) column (5 μm, 100 Å, 250 × 10 mm) with an isocratic elution (flow rate: 2 mL/min, 98% aqueous acetonitrile), yielding compounds 1 (3.5 mg), 2 (3.3 mg), and 3 (4.8 mg), respectively. The isolated compounds were identified as surfactin A, surfactin B, and surfactin C by comparison with their HR-MS and NMR data from the literature(1, 2).

- Surfactin A: Amorphous powder; UV(MeOH) λmax (log ϵ) 194 (1.82), 222 (0.78) nm; 1H (700 MHz) and 13C NMR (175 MHz), see Figure S11 and Figure S12; HRESIMS (positive-ion mode) m/z 1008.6670 [M + H]+ (calcd. for C51H89N7O13, 1008.6597)
- Surfactin B: Amorphous powder; UV(MeOH) λmax (log ϵ) 192 (1.53), 224 (0.34) nm; 1H (700 MHz) NMR, see Figure S14; HRESIMS (positive-ion mode) m/z 1022.6840 [M + H]+ (calcd. for C52H91N7O13, 1022.6753)
- Surfactin C: Amorphous powder; UV(MeOH) λmax (log ϵ) 194 (1.68), 222 (0.62) nm; 1H (700 MHz) and 13C NMR (175 MHz), see Figure S16 and Figure S17; HRESIMS (positive-ion mode) m/z 1036.6970 [M + H]+ (calcd. for C53H93N7O13, 1036.6910)

Comparison of chromatograms of 100% P1 fractions and commercial surfactin samples

The comparative analysis of HPLC chromatograms was carried out using an Agilent 1290 UPLC with a G6550A Q-TOF system. Optimal chromatographic conditions for the separation of all samples were achieved using a mobile phase consisting of Buffer A (water + 0.1% formic acid) and Buffer B (acetonitrile + 0.1% formic acid), with the following gradient: 0–0.5 min 2% B, 0.5–10 min 2–95% B, 10–12 min 95% B, 12–12.1 min 95–2% B, 12.1–15 min 2% B. An Agilent Zorbax Eclipse Plus RRHD C18 column (2.1 x 100mm, 1.8um) was used for the analysis with a flow rate set to 0.40 ml/min and an injection volume of 10.0 μl. The total run time was 15 min at a column oven temperature of 30℃. The detection wavelength was set at 210 nm, where all surfactins exhibited maximum absorption.

References

1. Tang J-S, Gao H, Hong K, Yu Y, Jiang M-M, Lin H-P, Ye W-C, Yao X-S. 2007. Complete assignments of (1)H and (13)C NMR spectral data of nine surfactin isomers. Magn Reson Chem MRC 45:792–796.

2. Pang X, Zhao J, Fang X, Liu H, Zhang Y, Cen S, Yu L. 2017. Surfactin derivatives from Micromonospora sp. CPCC 202787 and their anti-HIV activities. J Antibiot (Tokyo) 70:105–108.

Research supported by the Office of Army Research. The views expressed in this paper are those of the authors and do not reflect the official policy or position of the Department of the Army, Department of Defense, or the U.S. Government.

Supplemental Figures

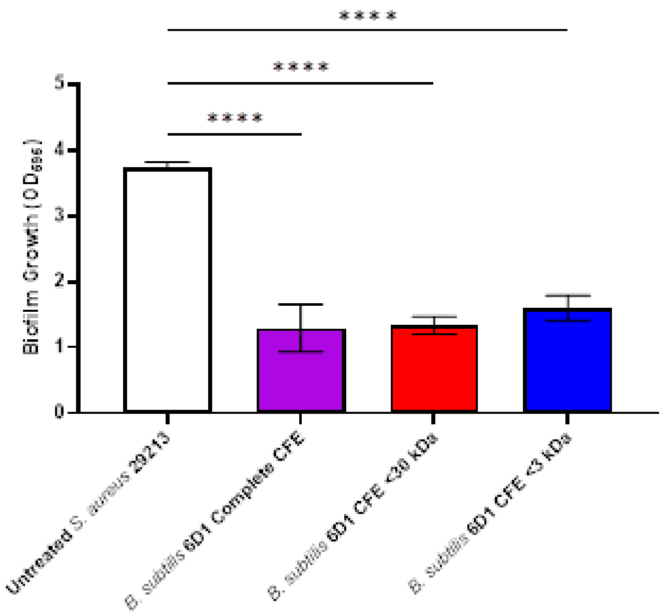

Figure S1. *B. subtilis* 6D1 CFE harboring low molecular weight metabolites (<3kDa) obtained through centrifugal filtration maintain antibiofilm activity against *S. aureus* ATCC 29213. *Bacillus* CFE fractions (>30kDa, <30kDa, and <3kDa) obtained via centrifugal filtration were measured for antibiofilm activity to narrow the size range of potentially active compounds. Error bars represent mean ± SD across 12 independent replicates. Differences were analyzed using a 2-tailed unpaired t-test where (****) P<0.0001 compared to the control

Research supported by the Office of Army Research. The views expressed in this paper are those of the authors and do not reflect the official policy or position of the Department of the Army, Department of Defense, or the U.S. Government.

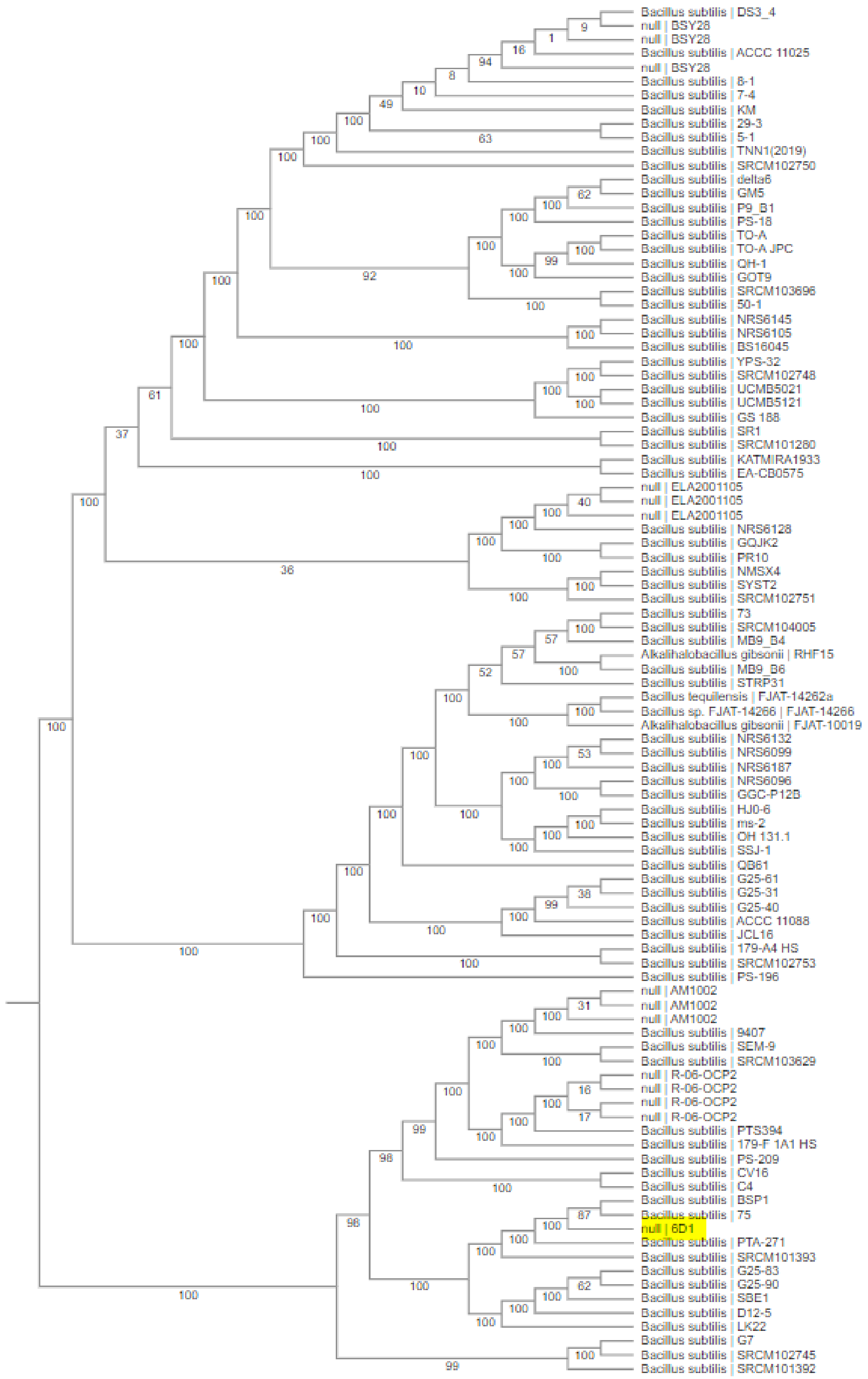

Figure S2. Phylogenetic analysis comparing *B. subtilis* 6D1 (highlighted) to 98 similarly identified Refseq genomes using 500 random single copy protein coding genes. *B. subtilis* 75 (Gene Bank Accession # CP045825) and *B. subtilis* PTA-271 (Gene Bank Accession # JACERQ010000010.1)

Research supported by the Office of Army Research. The views expressed in this paper are those of the authors and do not reflect the official policy or position of the Department of the Army, Department of Defense, or the U.S. Government.

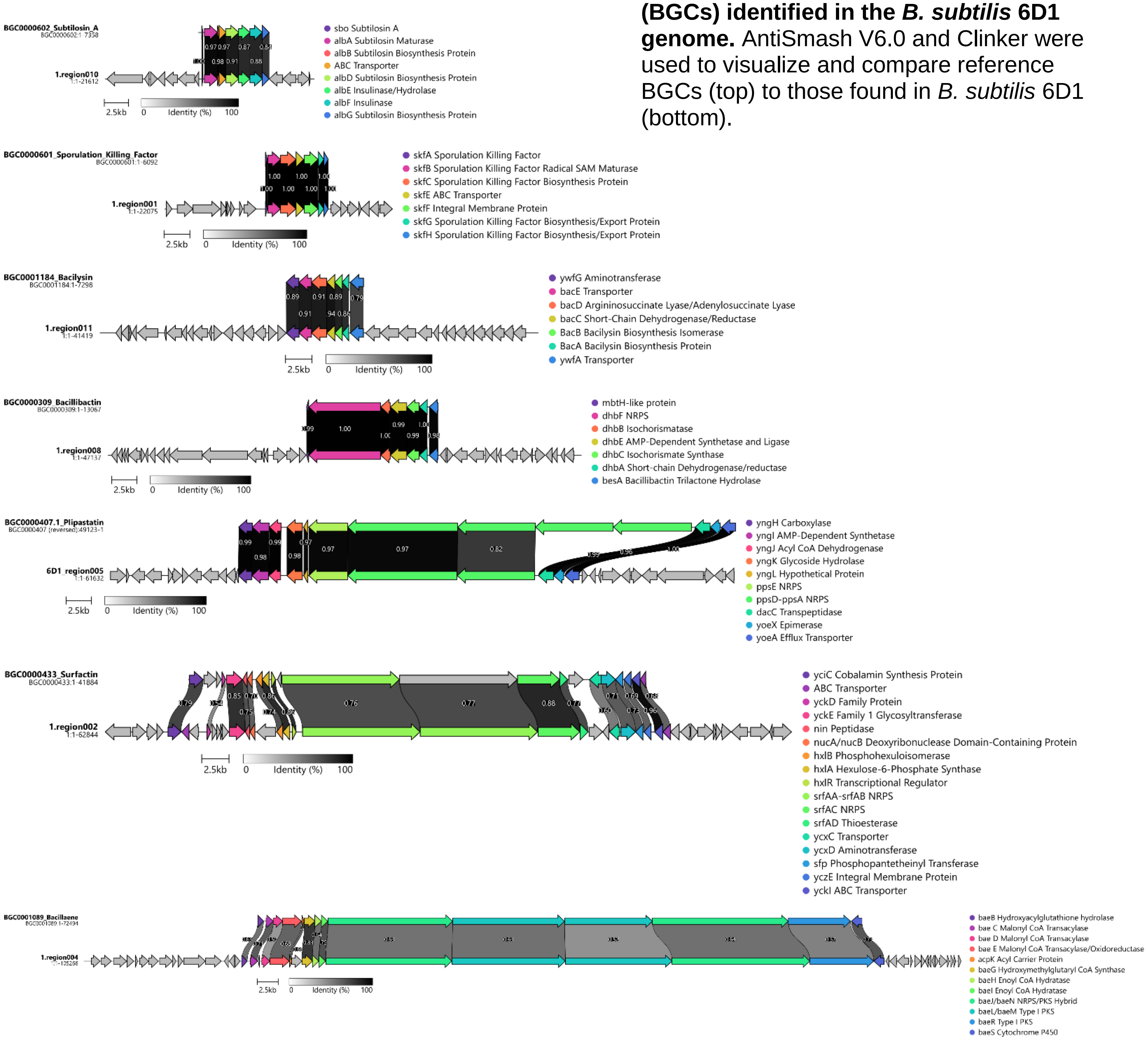

Figure S3. Biosynthetic gene clusters (BGCs) identified in the *B. subtilis* 6D1 genome. AntiSmash V6.0 and Clinker were used to visualize and compare reference BGCs (top) to those found in *B. subtilis* 6D1 (bottom).

Research supported by the Office of Army Research. The views expressed in this paper are those of the authors and do not reflect the official policy or position of the Department of the Army, Department of Defense, or the U.S. Government.

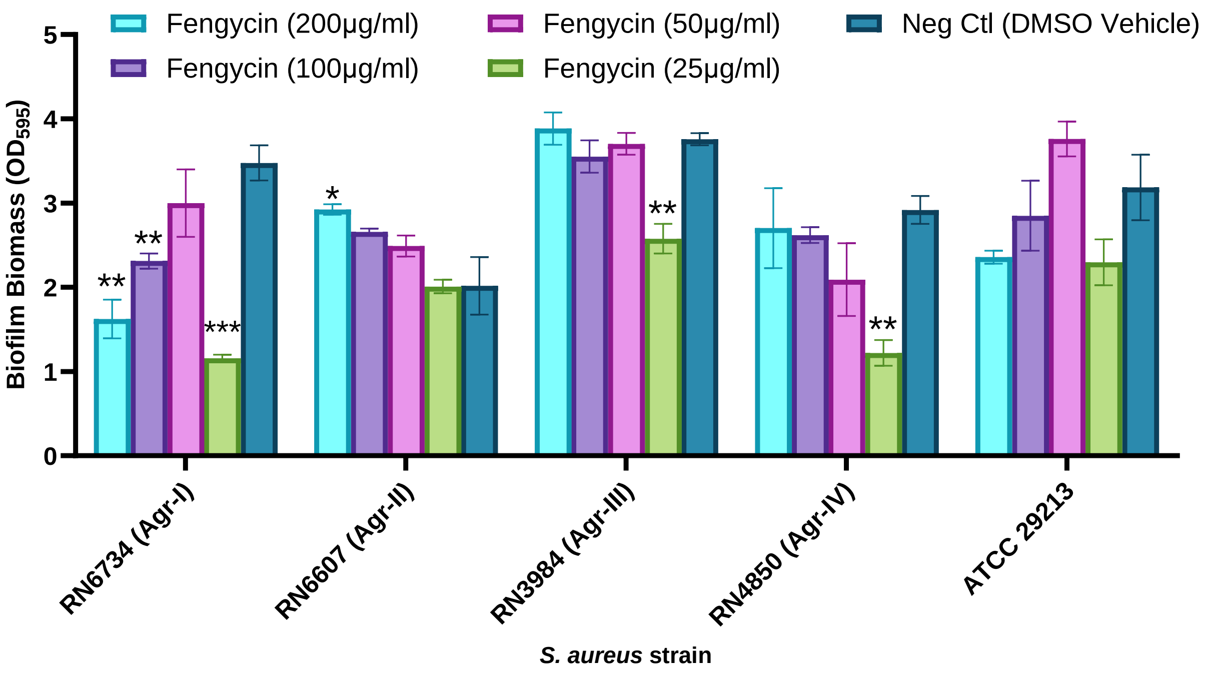

Figure S4. Commercial HPLC grade fengycin obtained from *B. subtilis* increases *S. aureus* biofilm formation in a concentration and strain specific manner. Error bars represent mean ± SD across 8 independent replicates; Differences were analyzed using a 2-tailed unpaired t-test where (*) $P<0.05$ (**) $P<0.01$ (***) $P<0.001$ compared to the control.

Research supported by the Office of Army Research. The views expressed in this paper are those of the authors and do not reflect the official policy or position of the Department of the Army, Department of Defense, or the U.S. Government.

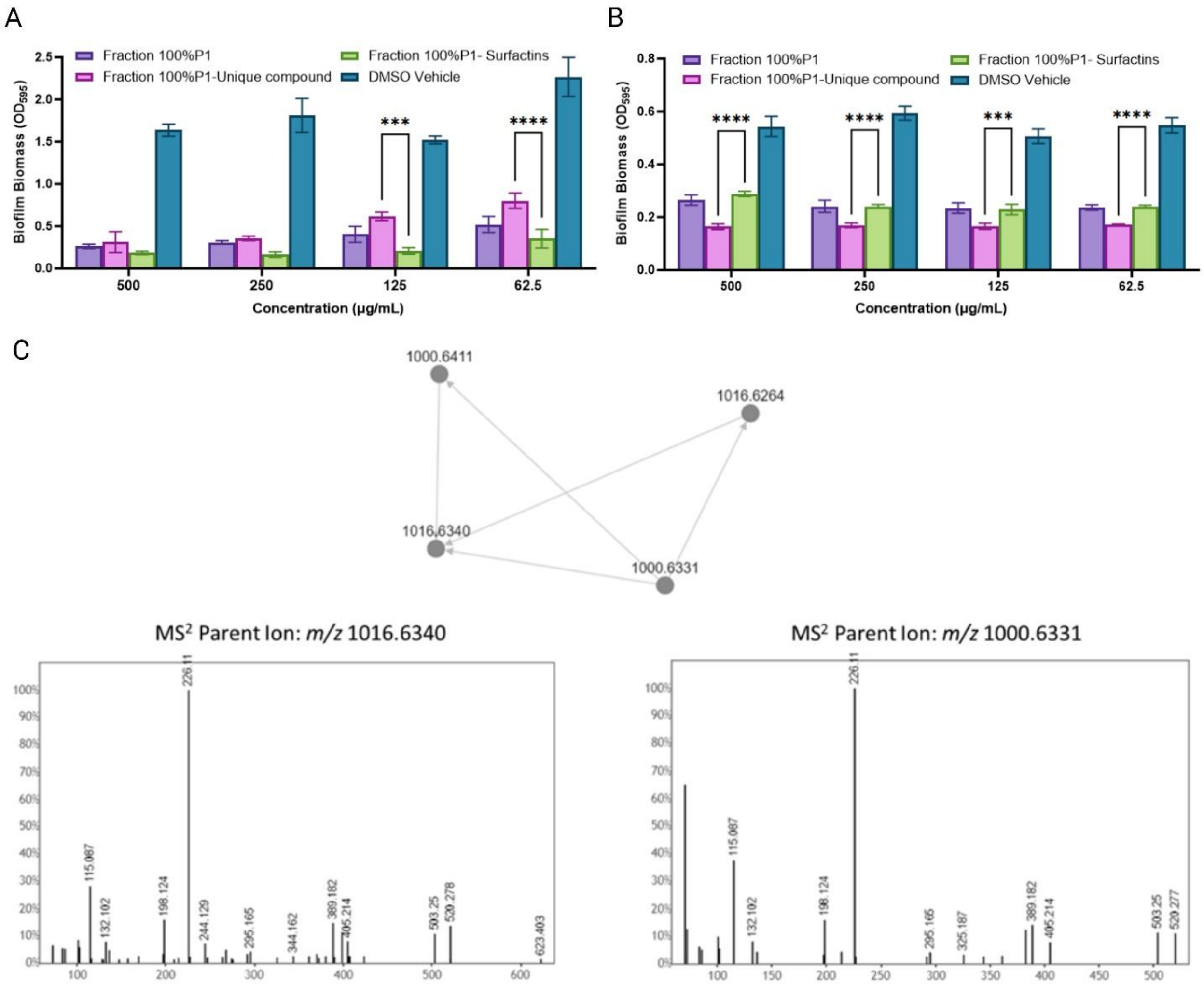

Figure S5. Unique compound identified in *B. subtilis* 6D1 fraction 100%P1 inhibits *S. aureus* biofilm growth and disrupts mature biofilm.
(A) Biofilm growth inhibition quantified using crystal violet (CV) staining. Either DMSO or *B. subtilis* 6D1 refined fractions were applied to *S. aureus* populations at T0 and incubated statically at 37 ºC for 24 hours. Residual biofilm biomass was quantified by CV staining and averaged across three independent experiments. **(B)** Biofilm disruption quantified using CV straining. Either DMSO or *B. subtilis* 6D1 refined fractions were applied to *S. aureus* 24-hour mature biofilms and incubated at 37ºC shaking at 100RPM for 2 hours. Residual biofilm biomass was quantified by crystal violet staining and averaged across three independent experiments. Error bars represent mean ± SD across 12 independent replicates. Differences were analyzed using a 2-tailed unpaired t-test where (****) P<0.0001, (***) P<0.001 **(C)** LC-MS/MS spectra and molecular network of *B. subtilis* 6D1 fraction 100%P1 novel compound. Nodes (m/z values) that are connected have a cosine score of at least 0.7 and represent similar molecules. Black spectra represent peaks identified in fraction P1-100% that did not match any known molecules in the GNPS database. The x-axis represents m/z and the y-axis represents the relative abundance of the various ions.

Research supported by the Office of Army Research. The views expressed in this paper are those of the authors and do not reflect the official policy or position of the Department of the Army, Department of Defense, or the U.S. Government.

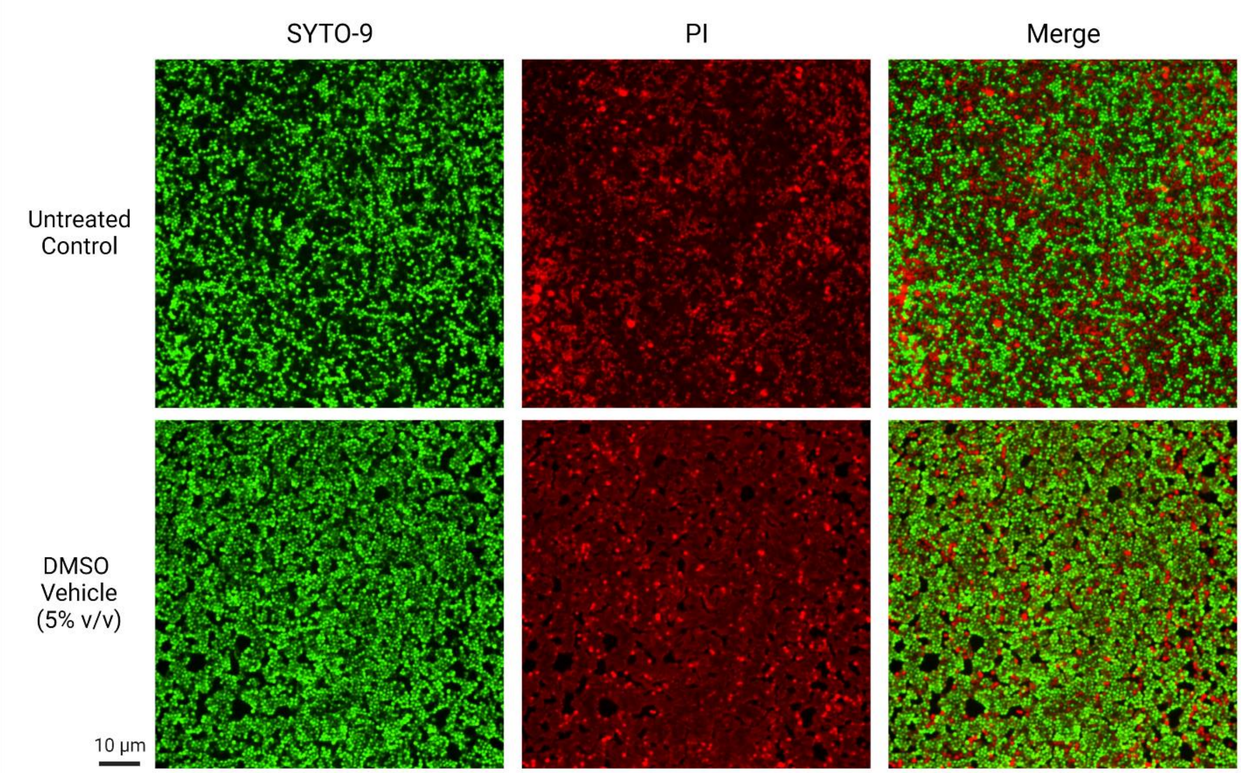

Figure S6. DMSO applied up to 5% v/v does not alter *S. aureus* ATCC 29213 biofilm growth or increase cell death compared to an untreated control. Scale bar = 10µm**.**

Research supported by the Office of Army Research. The views expressed in this paper are those of the authors and do not reflect the official policy or position of the Department of the Army, Department of Defense, or the U.S. Government.

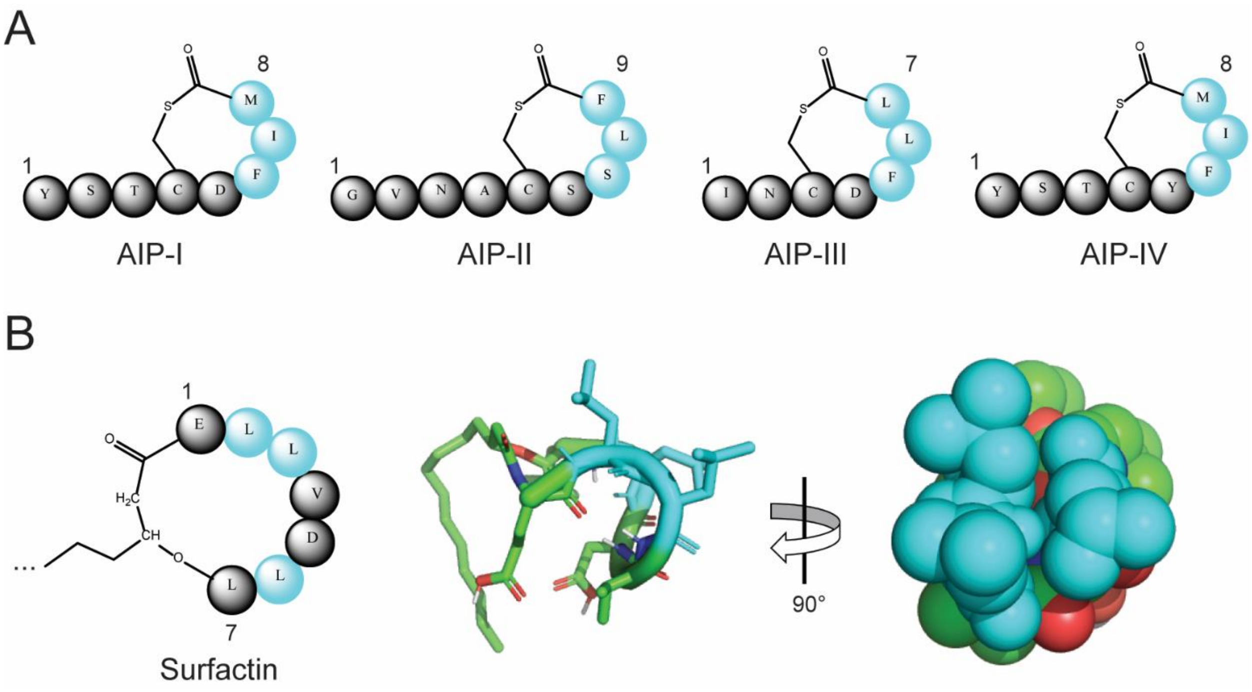

Figure S7: Comparison of the surfactins to *S. aureus* AIPs. A) Cartoon depiction of the *S. aureus* autoinducing peptides. Terminal residues which form the "hydrophobic knob" are highlighted in cyan. **B)** Structural depiction of the cyclic peptide of the surfactins. Leu2, Leu3, and Leu6 which create a similar "hydrophobic knob" are highlighted in cyan. "…" denotes continuation of the fatty acid tail. The structure of surfactin was modeled using the Protein Data Bank (PDB) file 2NPV. The PDB file was imported into PyMOL for visualization and molecular cartoons were generated using ChemDraw.

Research supported by the Office of Army Research. The views expressed in this paper are those of the authors and do not reflect the official policy or position of the Department of the Army, Department of Defense, or the U.S. Government.

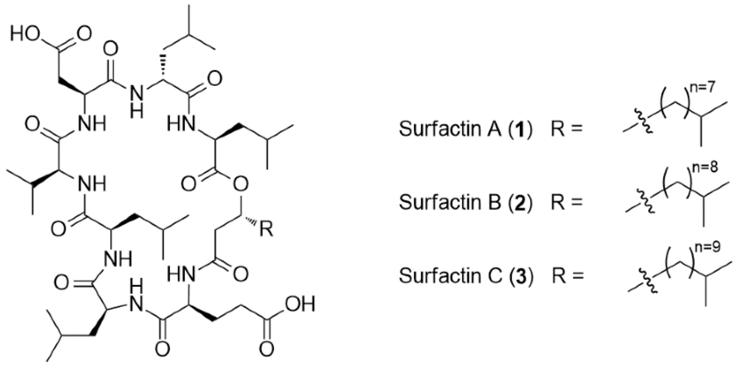

Figure S8. **Chemical structures of isolated surfactins from *B. subtilis* 6D1**

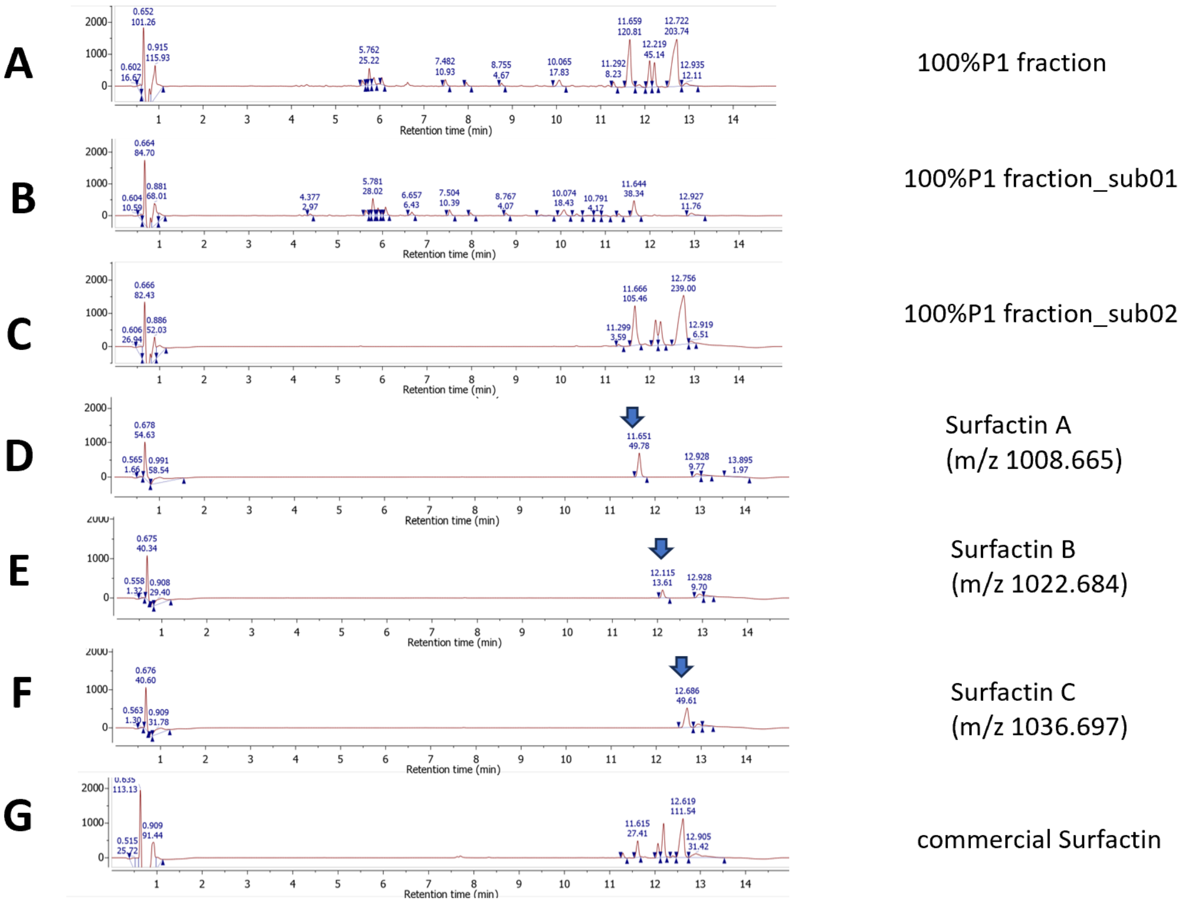

Figure S9. **UV (210nm) chromatograms of active fractions and isolated surfactins**. **A)** 100%P1 fraction; **B)** 100%P1-sub01 fraction (unique compound); **C)** 100%P1-sub02 fraction (surfactins); **D)** surfactin A; **E)** surfactin B; **F)** surfactin C; **G)** commercial surfactin

Research supported by the Office of Army Research. The views expressed in this paper are those of the authors and do not reflect the official policy or position of the Department of the Army, Department of Defense, or the U.S. Government.

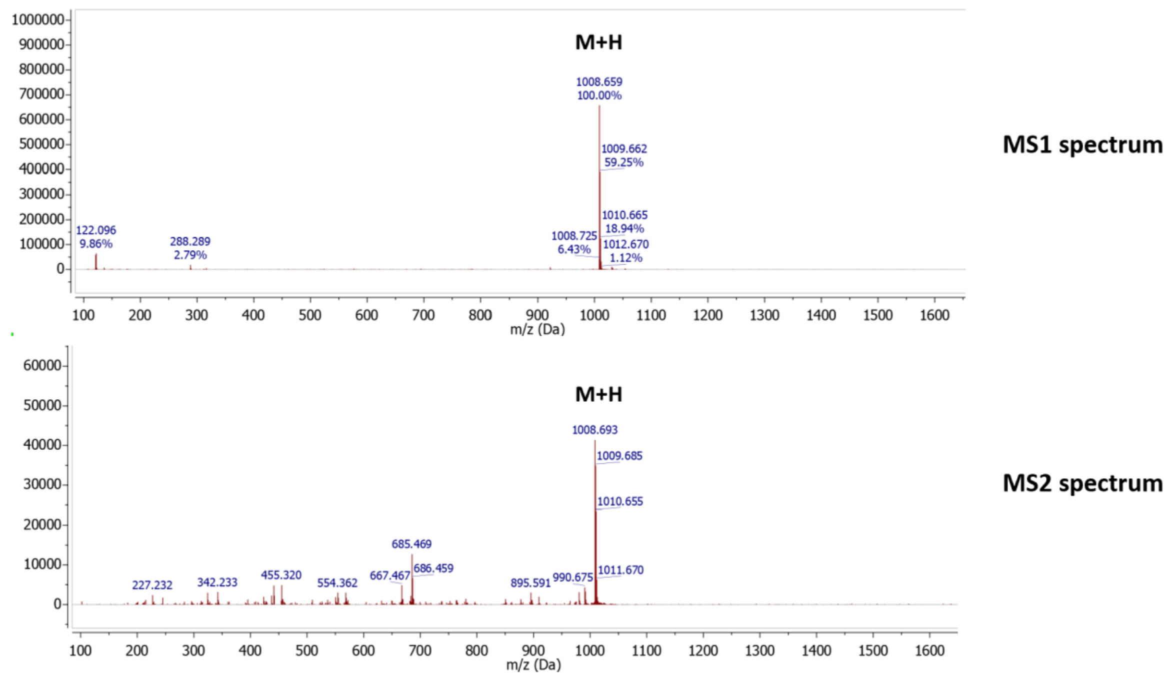

Figure S10. **HR-ESI-MS data of Surfactin A**

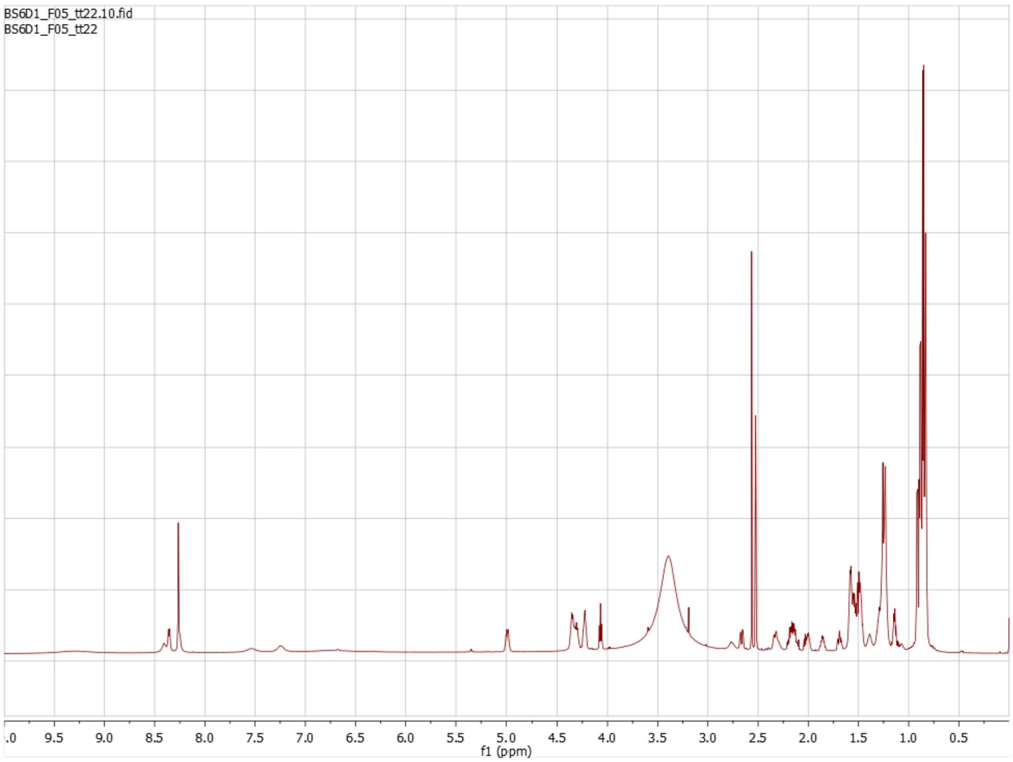

Figure S11. **The ^{1}H NMR spectrum of Surfactin A (DMSO-*d6*, 700 MHz)**

Research supported by the Office of Army Research. The views expressed in this paper are those of the authors and do not reflect the official policy or position of the Department of the Army, Department of Defense, or the U.S. Government.

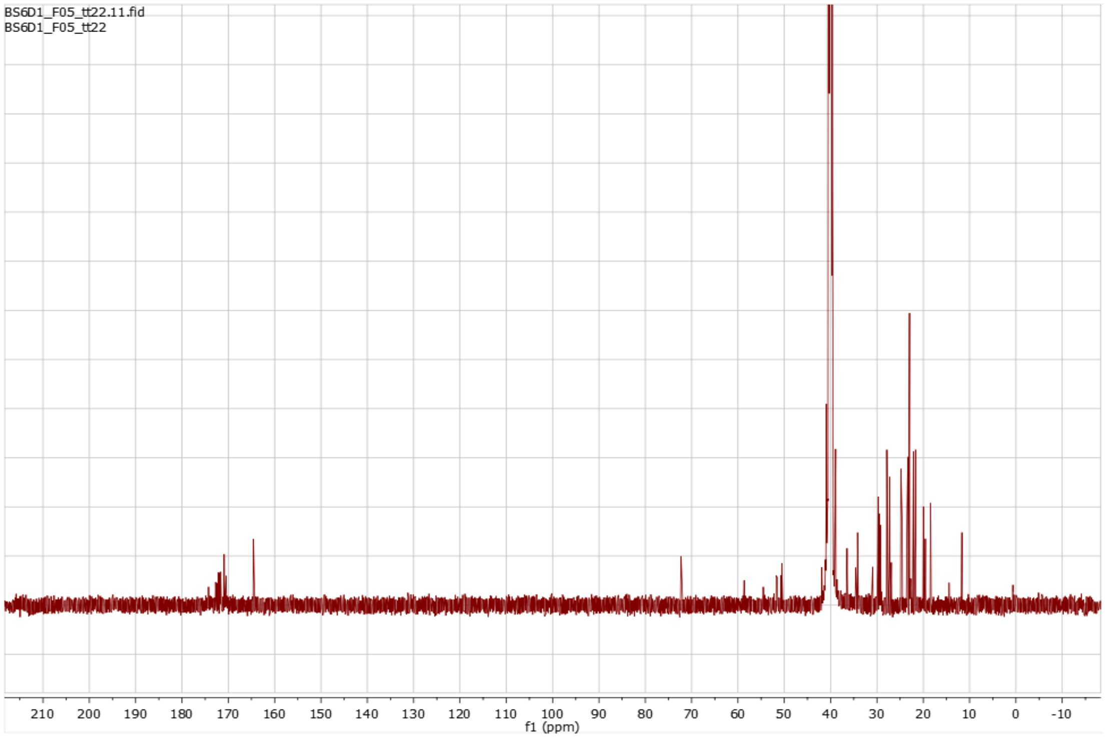

Figure S12. **The ^{13}C NMR spectrum of Surfactin A (DMSO-*d6*, 175 MHz)**

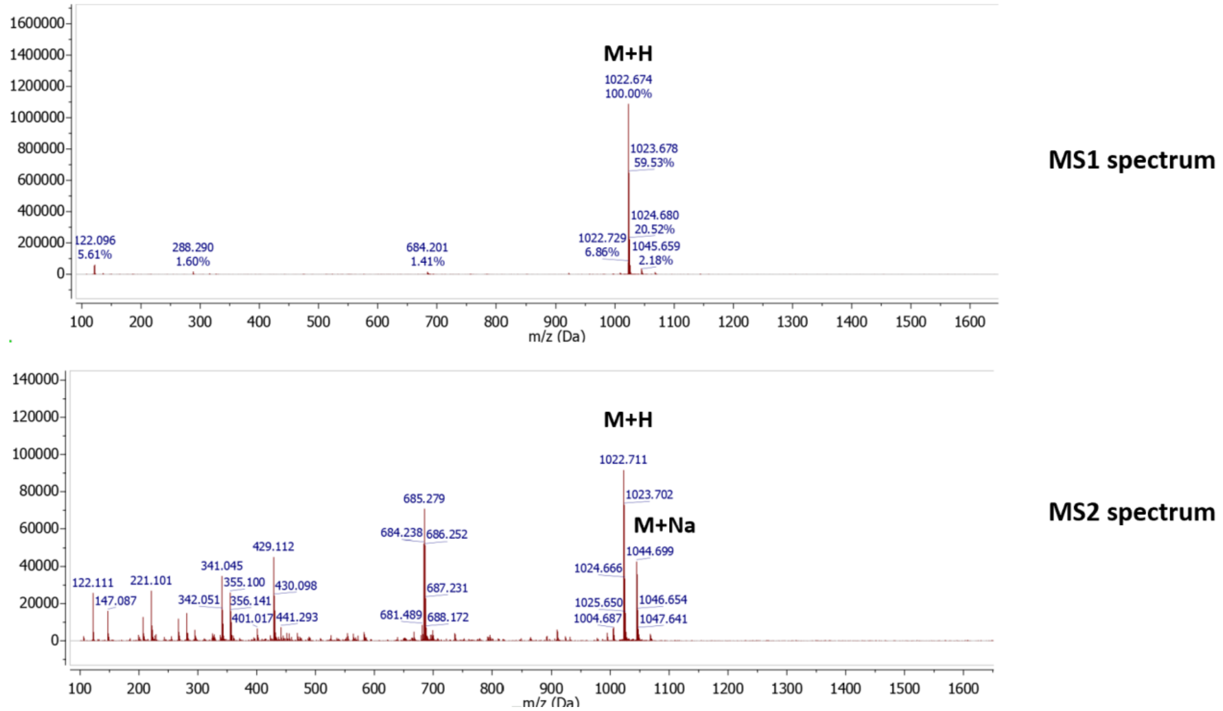

Figure S13. **HR-ESI-MS data of Surfactin B**

Research supported by the Office of Army Research. The views expressed in this paper are those of the authors and do not reflect the official policy or position of the Department of the Army, Department of Defense, or the U.S. Government.

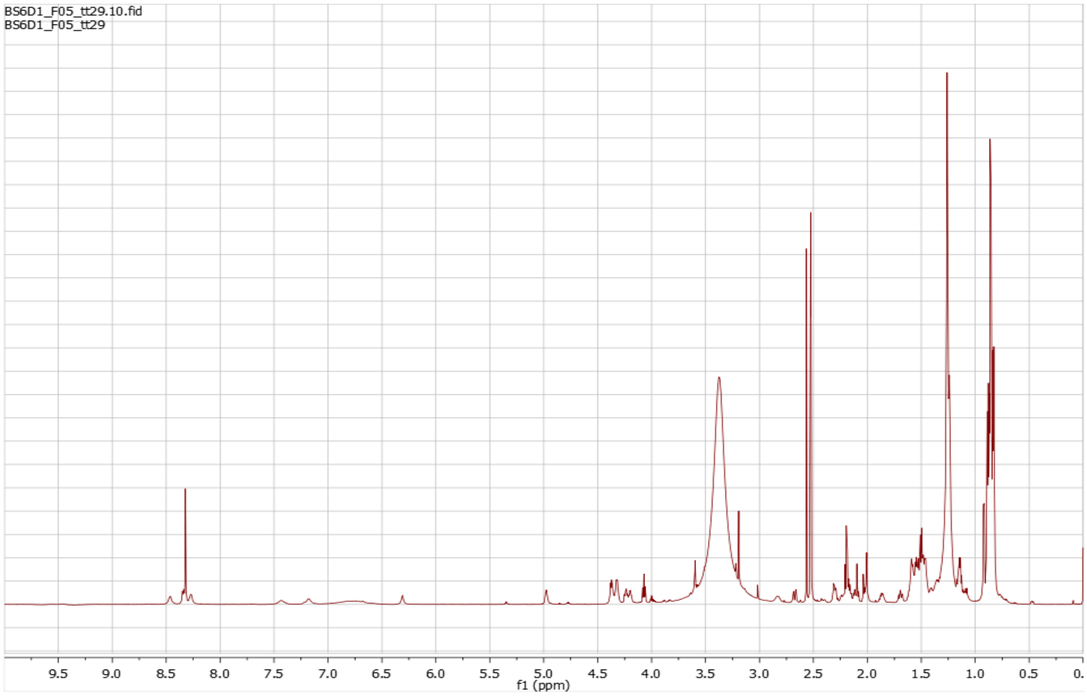

Figure S14. **The** [1]**H NMR spectrum of Surfactin B (DMSO-*d6*, 700 MHz)**

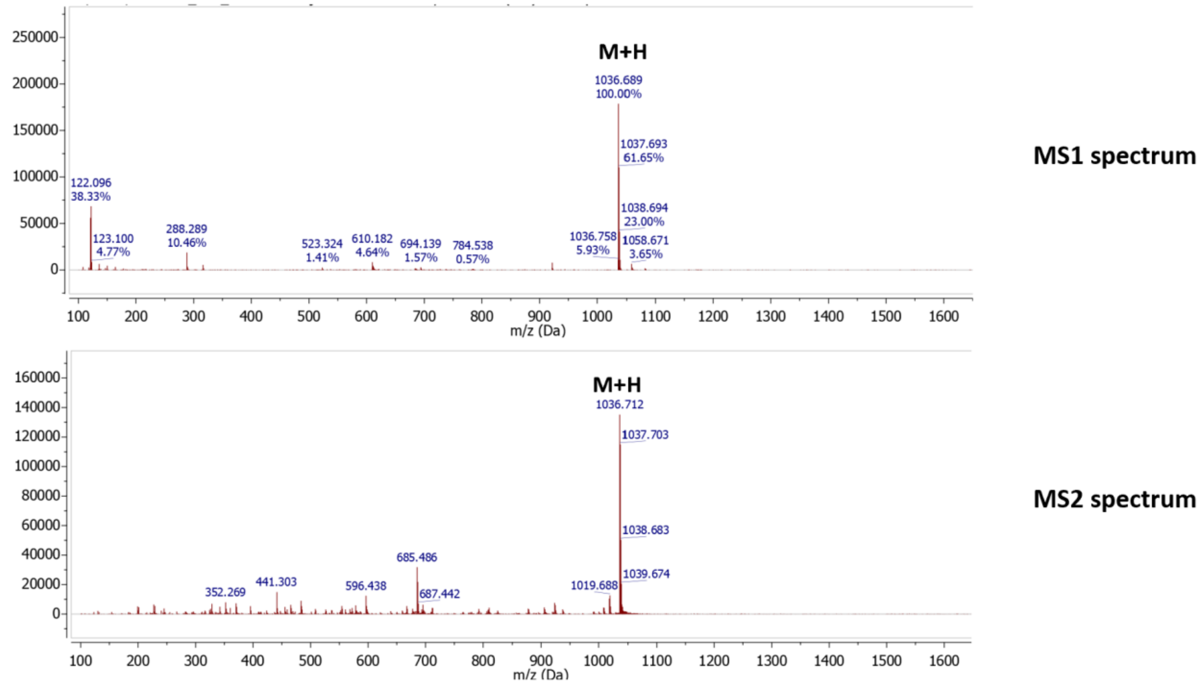

Figure S15. **HR-ESI-MS data of Surfactin C**

Research supported by the Office of Army Research. The views expressed in this paper are those of the authors and do not reflect the official policy or position of the Department of the Army, Department of Defense, or the U.S. Government.

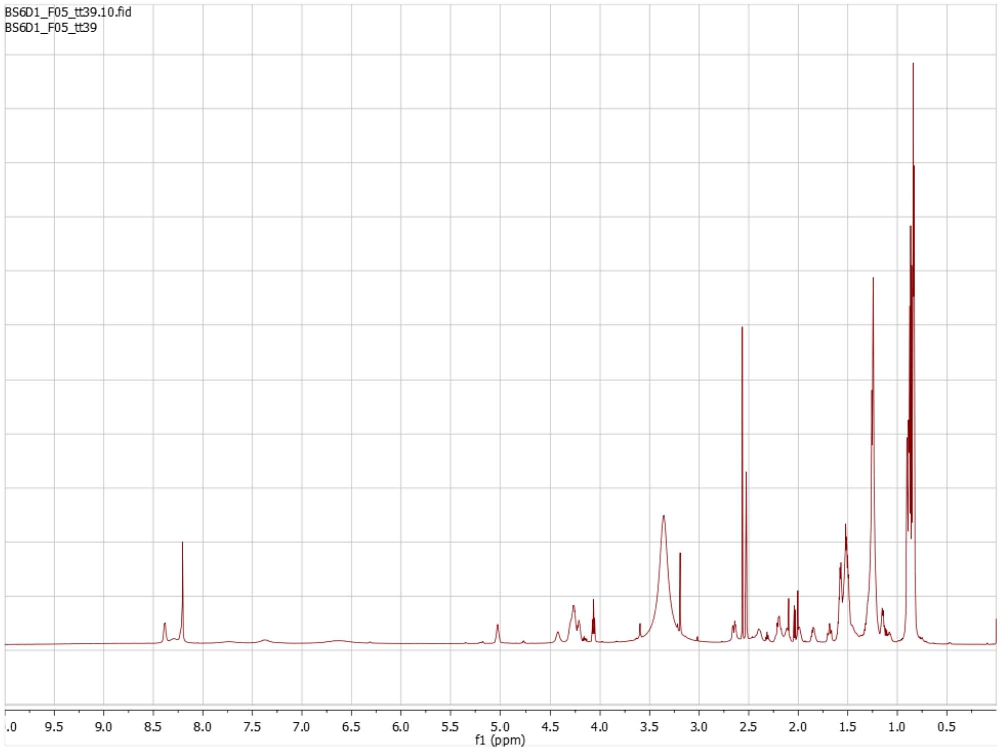

Figure S16. The ^{1}H NMR spectrum of Surfactin C (DMSO-d_6, 700 MHz)

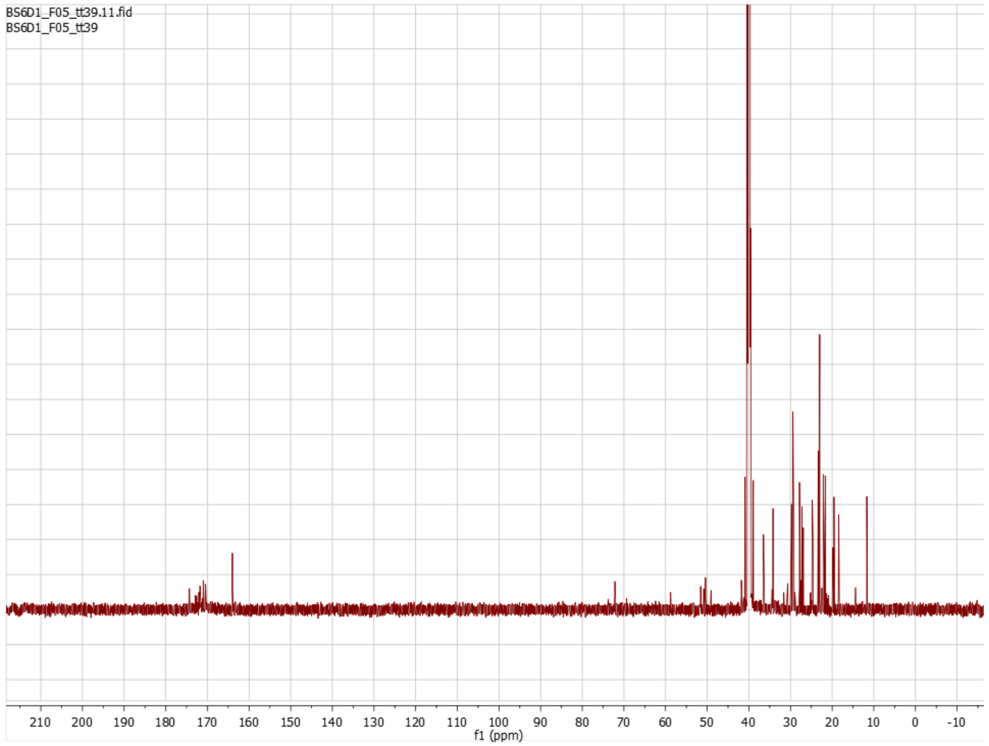

Figure S17. The ^{13}C NMR spectrum of Surfactin C (DMSO-d_6, 175 MHz)

Research supported by the Office of Army Research. The views expressed in this paper are those of the authors and do not reflect the official policy or position of the Department of the Army, Department of Defense, or the U.S. Government.
