# Supplementary material for: Bacillus subtilis-derived peptides disrupt quorum sensing and biofilm assembly in multidrug-resistant Staphylococcus aureus
Source: mSystems. 2024 Jul 11;9(8):e00712-24. doi: 10.1128/msystems.00712-24 (PMC11334493; doi:10.1128/msystems.00712-24)
Supplement: Supplemental Tables — Tables S1-S4. [file msystems.00712-24-s0002.docx]

**Supplementary Tables**

**Table S1. Bacterial Strain List**

| **Strain Name** | **Reference** | **Source** |
| --- | --- | --- |
| *S. aureus* ATCC 29213 | American Type Culture Collection |  |
| *S. aureus* ATCC G17 | American Type Culture Collection |  |
| *S. aureus* CLIN-A | This Study | Clinical sample of human wound infection |
| *S. aureus* CLIN-B | This Study | Clinical sample of human wound infection |
| *S. aureus* CLIN-C | This Study | Clinical sample of human wound infection |
| *S. aureus* CLIN-D | This Study | Clinical sample of human wound infection |
| *S. aureus* CLIN-E | This Study | Clinical sample of human wound infection |
| *S. aureus* CLIN-F | This Study | Clinical sample of human wound infection |
| *S. aureus* CLIN-G | This Study | Clinical sample of human wound infection |
| *S. aureus* 5917 | This Study | Clinical sample of human wound infection |
| *S. aureus* 5918 | This Study | Clinical sample of human wound infection |
| *S. aureus* 5919 | This Study | Clinical sample of human wound infection |
| *S. aureus* 5920 | This Study | Clinical sample of human wound infection |
| *S. aureus* 5921 | This Study | Titanium implant causing human infection |
| *S. aureus* 5922 | This Study | Clinical sample of human wound infection |
| *S. aureus* 6014 | This Study | Clinical sample of human wound infection |
| *S. aureus* 6015 | This Study | Clinical sample of human wound infection |
| *S. aureus* 6016 | This Study | Clinical sample of human wound infection |
| *S. aureus* 6017 | This Study | Clinical sample of human abscess |
| *S. aureus* 6018 | This Study | Clinical sample of human wound infection |
| *S. aureus* 6019 | This Study | Clinical sample of human wound infection |
| *S. aureus* 6020 | This Study | Clinical sample of human wound infection |
| *S. aureus* 6021 | This Study | Clinical sample of human wound infection |
| *S. aureus* 6533 | This Study | Clinical sample of human wound infection |
| *S. aureus* 6535 | This Study | Clinical sample of human wound infection |
| *S. aureus* 6536 | This Study | Clinical sample of human wound infection |
| *S. aureus* 6537 | This Study | Clinical sample of human wound infection |
| *S. aureus* 6538 | This Study | Clinical sample of human wound infection |
| *S. aureus* 6539 | This Study | Clinical sample of human wound infection |
| *S. aureus* 6540 | This Study | Clinical sample of human wound infection |
| *S. aureus* 6541 | This Study | Clinical sample of human wound infection |
| *S. aureus* 6563 | This Study | Clinical sample of human wound infection |
| *S. aureus* 6564 | This Study | Clinical sample of human wound infection |
| *S. aureus* 6565 | This Study | Titanium implant causing human infection |
| *S. aureus* 6566 | This Study | Clinical sample of human wound infection |
| *S. aureus* 6645 | This Study | Clinical sample of human wound infection |
| *S. aureus* 6647 | This Study | Clinical sample of human wound infection |
| *S. aureus* RN6734 (Prototypical agr-I) | Ji et al. 1997 |  |
| *S. aureus* RN6607 (Prototypical agr-II) | Ji et al. 1997 |  |
| *S. aureus* RN3984 (Prototypical agr-III) | Ji et al. 1997 |  |
| *S. aureus* RN4850 (Prototypical agr-IV) | Ji et al. 1997 |  |
| *S. aureus* RN7206 (agr-null derivative of RN6734) | Ji et al. 1997 |  |
| *S. aureus* RSM5 | This Study | Nasal lavage specimen from healthy child |
| *S. aureus* RSM97 | This Study | Nasal lavage specimen from healthy child |
| *S. aureus* RSM256 | This Study | Nasal lavage specimen from healthy child |
| *S. aureus* RSM424 | This Study | Nasal lavage specimen from sick child |
| *S. aureus* RSM486 | This Study | Nasal lavage specimen from healthy child |
| *S. epidermidis* ATCC 12228 | American Type Culture Collection |  |
| *S. epidermidis* RSM78 | This Study | Nasal lavage specimen from sick child |
| *S. epidermidis* RSM133 | This Study | Nasal lavage specimen from sick child |
| *S. epidermidis* RSM242 | This Study | Nasal lavage specimen from healthy child |
| *S. epidermidis* RSM268 | This Study | Nasal lavage specimen from sick child |
| *S. epidermidis* RSM425 | This Study | Nasal lavage specimen from sick child |
| *B. subtilis* NCIB 3610 | *Bacillus* Genetic Stock Center |  |
| *B. subtilis* 6D1 | This Study | River sediment from Scarboro Creek |
| *B. subtilis* 9B5 | This Study | River sediment from Scarboro Creek |
| *B. pumilus* 11D12 | This Study | Bovine manure from Kewaunee Country |
| *B. pumilus* 10D6 | This Study | River sediment from CAFO impacted stream |
| *B. pumilus* 11G1 | This Study | Bovine manure from Kewaunee Country |
| *B. pumilus* 11F9 | This Study | Bovine manure from Kewaunee Country |
| *B. pumilus* 11B2 | This Study | Bovine manure from Kewaunee Country |
| *Bacillus* 11A10 | This Study | River sediment from CAFO impacted stream |
| *Bacillus* 11A12 | This Study | River sediment from CAFO impacted stream |
| *Bacillus* 3A3 | This Study | River sediment from Kewaunee River source |
| *Bacillus* 10C11 | This Study | River sediment from Scarboro Creek |
| *Bacillus* 2G11 | This Study | River sediment outside Kewaunee watershed |
| *Bacillus* 11G5 | This Study | Bovine manure from Kewaunee Country |
| *Bacillus* 3H6 | This Study | River sediment from Casco Creek Crevice |
| *Bacillus* 1E1 | This Study | River sediment outside Kewaunee watershed |
| *Bacillus* 5A12 | This Study | River sediment from Casco Creek Crevice |

**Table S2. List of Gene Expression Targets and Primer Melting Temperatures (Tm)**

| **Gene** | **Forward primer (5’-3’)** | **Reverse primer (5’-3’)** | **Tm** |
| --- | --- | --- | --- |
| *agrA* | TGCGAAGACGATCCAAAAC | TTTAGCTTGCTCAAGCACCTC | 60°C |
| *aur* | GATGGTCGCACATTCACAAG | CGCCTGACTGGTCCTTATATTC | 60°C |
| *lrgB* | TATTGCCCGAGGATTAGCAC | CAAAGACAGGCACAACTGCTAC | 60°C |
| *lukD* | GTACTTAAGGCAGCCGGAAAC | CGCCCCAATAAAACTGTGAG | 60°C |
| *sigB* | TGATCGCGAACGAGAAATC | ATTGCCGTTCTCTGAAGTCG | 60°C |
| *capC* | CATCCAGAGCGGAATAAAGC | CGGAAATACCCGCTAATGAC | 59.5°C |
| *isaA* | TCCGACAAACACTGTTGACC | AATCCCCAAGCACCTAAACC | 59.5°C |
| *lytM* | ACGGTGTCGACTATGCAATG | ATTGCCGCCACCATAGTTAC | 59.5°C |
| *saeR* | CCAAGGGAACTCGTTTTACG | ACGCATAGGGACTTCGTGAC | 59.5°C |
| *fnbB* | GAACATGGTCAAGCACAAGG | ACGCCATAATTACCGTGACC | 59°C |
| *hla* | TCTTGGAACCCGGTATATGG | AGCGAAGTCTGGTGAAAACC | 59°C |
| *hlb* | GTGCCAAAGCCGAATCTAAG | ATCAGCGCGTTTATATTGTCC | 59°C |
| *hld* | AAGGAAGGAGTGATTTCAATGG | TTTGTTCACTGTGTCGATAATCC | 59°C |
| *psmα* | TCAAAAGCTTAATCGAACAATTCAC | AATGGCCCCCTTCAAATAAG | 59°C |
| *sarA* | TTGCTTTGAGTTGTTATCAATGG | CAATACAGCGAATTCTTCAAAGC | 59°C |
| *cidA* | CTTAGCCGGCAGTATTGTTG | GTTTGCACCGTCTTCTACCC | 58.5°C |
| *clfB* | TTATGGTGGTGGAAGTGCTG | TGGACTTGGTTCTGGATCTG | 58.5°C |
| *RNAIII* | AAGCCATCCCAACTTAATAACC | GCACTGAGTCCAAGGAAACTAAC | 58.5°C |
| *rpoB* | ACAACCACTTGGCGGTAAAG | ATGCTTCAAGTGCCCATACC | 60°C |

**Table S3. List of Unique Genes Identified in *B. subtilis* 6D1 Genome**

| **Description** | **Seed ortholog** | **e-value** | **Max annotation level** | **COG category** |
| --- | --- | --- | --- | --- |
| Prophage endopeptidase tail | 279010.BL03515 | 1.90E-275 | Bacilli | Cell motility; Intracellular trafficking & secretion |
| Catalyzes the first step in the D-alanylation of lipoteichoic acid | 224308.BSU18320 | 0 | Bacilli | Secondary structure |
| Peptidase activity | 10717.Q9ZXF7_BPPH1 | 1.30E-102 | Siphoviridae | Function unknown |
| Phage terminase small subunit | 1274524.BSONL12_06108 | 2.10E-101 | Bacilli | Replication and repair |
| Protein of unknown function (DUF669) | 279010.BL03492 | 1.67E-93 | Bacilli | Function unknown |
| Phosphoribosyl-ATP pyrophosphohydrolase | 1408303.JNJJ01000009_gene2204 | 6.13E-53 | Bacilli | Function unknown |
| Sequence-specific DNA binding | 10717.Q786F1_BPPH1 | 2.01E-82 | Siphoviridae | Function unknown |
| Phage head-tail joining protein | 1274524.BSONL12_06148 | 6.10E-51 | Bacilli | Function unknown |
| BhlA holin family | 224308.BSU21420 | 4.44E-38 | Bacilli | Function unknown |
| Phage tail protein | 279010.BL03514 | 1.50E-131 | Bacilli | Function unknown |
| DNA gyrase B | 881953.E0YIY1_9CAUD | 4.70E-228 | Caudovirales | Function unknown |
| Nuclease activity | 10717.Q9ZXC2_BPPH1 | 3.10E-121 | Siphoviridae | Function unknown |
| Phage portal protein | 1434319.W5RVB1_9CAUD | 1.50E-130 | Siphoviridae | Function unknown |
| Phage portal protein | 66692.ABC1331 | 1.10E-219 | Bacilli | Function unknown |
| DNA gyrase/topoisomerase IV, subunit A | 881953.E0YIY2_9CAUD | 9.40E-181 | Caudovirales | Function unknown |
| Major facilitator superfamily | 1348908.KI518625_gene1647 | 4.37E-26 | Bacilli | Amino Acid/carbohydrate/inorganic ion metabolism & transport |
| Recombinase | 10717.Q9T200_BPPH1 | 4.00E-288 | Siphoviridae | Function unknown |
| HNH endonuclease | 1274524.BSONL12_06103 | 2.80E-65 | Bacilli | Defense mechanism |
| Nucleoside 2-deoxyribosyltransferase | 1384057.CD33_16495 | 5.17E-62 | Bacilli | Nucleotide metabolism and transport |
| PemK-like, MazF-like toxin of type II toxin-antitoxin system | 1297581.H919_00930 | 4.08E-18 | Bacilli | Signal transduction |
| SPP1 phage holin | 240302.BN982_00173 | 7.04E-14 | Bacilli | Function unknown |
| capsid protein | 66692.ABC1333 | 1.90E-204 | Bacilli | Function unknown |
| Terminase | 1274524.BSONL12_06113 | 0 | Bacilli | Function unknown |
| Guanylate kinase homologues. | 596330.HMPREF0628_1632 | 2.92E-09 | Clostridia | Nucleotide metabolism and transport |
| DNA packaging | 224308.BSU26200 | 3.62E-59 | Bacilli | Replication and repair |
| Phage tail tube protein | 1196324.A374_08734 | 1.08E-35 | Bacilli | Function unknown |
| Hydrolase activity | 10717.Q9ZXC4_BPPH1 | 0 | Siphoviridae | Function unknown |
| DNA protection | 10717.Q9ZXC8_BPPH1 | 3.10E-119 | Siphoviridae | Function unknown |
| Caudovirus prohead serine protease | 240302.BN982_03592 | 4.50E-65 | Bacilli | Function unknown |
| N-acetylmuramoyl-L-alanine amidase activity | 1406785.U5PU90_9CAUD | 1.38E-85 | Caudovirales | Function unknown |
| DNA packaging | 1033739.CAEU01000069_gene8 | 4.26E-29 | Bacteria | Function unknown |
| Phage tail tape measure protein | 1274524.BSONL12_06173 | 0 | Bacilli | Cell cycle control and mitosis |
| AAA domain | 1178540.BA70_16395 | 7.50E-211 | Bacilli | Cell cycle control and mitosis |
| 3D domain | 66692.ABC0489 | 1.51E-29 | Bacilli | Cell membrane biogenesis |
| DNA primase activity | 1434319.W5RVC4_9CAUD | 1.80E-113 | Siphoviridae | Function unknown |
| Phage tail tape measure protein | 1340434.AXVA01000005_gene4862 | 2.10E-251 | Bacilli | Cell cycle control and mitosis |
| Phage integrase family | 1434319.W5RV39_9CAUD | 3.70E-122 | Siphoviridae | Function unknown |
| Ribonucleotide reductase, small chain | 1434319.W5RVC6_9CAUD | 7.50E-130 | Siphoviridae | Function unknown |
| Phage tail protein | 411465.PEPMIC_00022 | 2.30E-239 | Firmicutes | Function unknown |
| HNH endonuclease | 1500386.A0A060AKD6_9CAUD | 3.64E-41 | Siphoviridae | Function unknown |
| Pfam:Peptidase_M78 | 10717.Q9T201_BPPH1 | 4.72E-92 | Siphoviridae | Function unknown |
| Helix-turn-helix | 86416.Clopa_0552 | 1.62E-12 | Clostridia | Transcription |
| Aspartate phosphatase | 720555.BATR1942_06825 | 5.40E-135 | Bacilli | Function unknown |
| Phage terminase, small subunit | 1500386.A0A060AKR4_9CAUD | 4.92E-39 | Siphoviridae | Function unknown |
| Immunity protein 70 | 1274524.BSONL12_12931 | 3.62E-86 | Bacilli | Function unknown |
| Immunity protein 70 | 1274524.BSONL12_06218 | 2.60E-82 | Bacilli | Function unknown |
| Phage terminase | 1033739.CAEU01000068_gene122 | 3.40E-261 | Bacilli | Function unknown |
| DNA helicase activity | 1500386.A0A060AB93_9CAUD | 1.60E-132 | Siphoviridae | Function unknown |
| Glycosyl hydrolases family 25 | 666686.B1NLA3E_01320 | 1.00E-145 | Bacilli | Cell membrane biogenesis |
| Transcriptional regulator | 265729.GS18_0220175 | 4.38E-13 | Bacilli | Transcription |
| Bacteriophage HK97-gp10, putative tail-component | 1274524.BSONL12_06153 | 2.81E-65 | Bacilli | Function unknown |
| YopX protein | 1536769.P40081_15180 | 4.03E-11 | Bacilli | Function unknown |
| PfkB family carbohydrate kinase | 272556.CF65_00429 | 3.71E-09 | Gamma-proteobacteria | Carbohydrate metabolism and transport |
| Replication initiator protein A (RepA) N-terminus | 1329250.WOSG25_090160 | 1.57E-15 | Bacilli | Function unknown |
| Endonuclease that specifically degrades the RNA of RNA- DNA hybrids | 1128398.Curi_c12400 | 4.61E-60 | Clostridia | Replication and repair |
| Uncharacterized protein YqaH | 1178540.BA70_16380 | 1.78E-28 | Bacilli | Function unknown |
| Phage gp6-like head-tail connector protein | 1274524.BSONL12_06143 | 8.77E-54 | Bacilli | Function unknown |
| Phage tail tube protein | 1274524.BSONL12_06163 | 3.20E-101 | Bacilli | Function unknown |
| Domain of unknown function (DUF1738) | 1415775.U729_3188 | 1.40E-71 | Clostridia | Replication and repair |
| Phage capsid family | 1034347.CAHJ01000091_gene1634 | 1.60E-108 | Bacilli | Function unknown |
| Integrase family | 1321778.HMPREF1982_00376 | 1.21E-92 | Clostridia | Replication and repair |
| Ribonucleoside-diphosphate reductase activity, thioredoxin disulfide as acceptor | 1434319.W5RV23_9CAUD | 0 | Siphoviridae | Function unknown |
| Bacterial DNA polymerase III alpha subunit | 1434319.W5RV95_9CAUD | 0 | Siphoviridae | Function unknown |
| Flavodoxin | 1444310.JANV01000144_gene363 | 6.76E-12 | Bacilli | Energy production and conversion |

**Table S4. List of Single Copy Orthologs Used for *B. subtilis* 6D1 Phylogenetic Analysis**

| **PGFam** | **Alignment Score** | **Alignment Length** | **Number of Sequences** | **Mean Square Frequency** | **Proposed Number of Gaps** | **Used In Analysis** | **Product** |
| --- | --- | --- | --- | --- | --- | --- | --- |
| PGF_02704551 | 34.62 | 1199 | 98 | 1 | 0 | TRUE | DNA-directed RNA polymerase beta' subunit (EC 2.7.7.6) |
| PGF_00045963 | 33.84 | 1148 | 98 | 0.999 | 0 | TRUE | Pyruvate carboxylase (EC 6.4.1.1) |
| PGF_07830674 | 29.51 | 878 | 98 | 0.996 | 0 | TRUE | Alanyl-tRNA synthetase (EC 6.1.1.7) |
| PGF_07058357 | 27.93 | 786 | 98 | 0.996 | 0 | TRUE | Single-stranded-DNA-specific exonuclease RecJ |
| PGF_04438983 | 27.81 | 774 | 98 | 1 | 0 | TRUE | ATP-dependent protease La (EC 3.4.21.53) Type I |
| PGF_12779560 | 27.46 | 775 | 98 | 0.986 | 0 | TRUE | Phage infection protein |
| PGF_01444054 | 27.28 | 749 | 98 | 0.997 | 0 | TRUE | ATP-dependent RNA helicase |
| PGF_01671660 | 26.69 | 801 | 98 | 0.943 | 0.046 | TRUE | Transcriptional regulator, AraC family |
| PGF_04883561 | 26.28 | 710 | 98 | 0.986 | 0 | TRUE | Assimilatory nitrate reductase large subunit (EC 1.7.99.4) |
| PGF_00840774 | 25.82 | 673 | 98 | 0.995 | 0 | TRUE | Heterodimeric efflux ABC transporter, multidrug resistance => BmrD subunit of BmrCD |
| PGF_06875694 | 25.76 | 668 | 98 | 0.997 | 0 | TRUE | Penicillin-binding protein 3 |
| PGF_04333086 | 25.73 | 668 | 98 | 0.995 | 0 | TRUE | DNA ligase (NAD(+)) (EC 6.5.1.2) |
| PGF_06459503 | 25.41 | 648 | 98 | 0.998 | 0 | TRUE | Serine/threonine protein kinase PrkC, regulator of stationary phase |
| PGF_00005140 | 25.32 | 646 | 98 | 0.996 | 0 | TRUE | ABC transporter-like sensor and permease protein YvcS |
| PGF_00960048 | 25.04 | 639 | 98 | 0.991 | 0.006 | TRUE | PTS system, fructose-specific IIA component (EC 2.7.1.202) / PTS system, fructose-specific IIB component (EC 2.7.1.202) / PTS system, fructose-specific IIC component |
| PGF_06841555 | 24.93 | 643 | 98 | 0.983 | 0.001 | TRUE | hypothetical protein |
| PGF_07573550 | 24.78 | 622 | 98 | 0.993 | 0 | TRUE | ABC transporter-like sensor and permease protein YxdM |
| PGF_02788357 | 24.66 | 611 | 98 | 0.998 | 0.001 | TRUE | Two-component sensor kinase SA14-24 |
| PGF_00012969 | 24.21 | 590 | 98 | 0.997 | 0 | TRUE | Phosphomethylpyrimidine synthase ThiC (EC 4.1.99.17) |
| PGF_00045994 | 24.16 | 585 | 98 | 0.999 | 0 | TRUE | Pyruvate kinase (EC 2.7.1.40) / Phosphohistidine swiveling domain |
| PGF_00066435 | 24.12 | 643 | 98 | 0.951 | 0.045 | TRUE | Asparagine synthetase [glutamine-hydrolyzing] (EC 6.3.5.4) YisO |
| PGF_10523783 | 23.89 | 572 | 98 | 0.999 | 0 | TRUE | Acetyl-CoA synthetase (EC 6.2.1.1) |
| PGF_00025227 | 23.87 | 571 | 98 | 0.999 | 0 | TRUE | Acetolactate synthase, catabolic (EC 2.2.1.6) |
| PGF_00055052 | 23.85 | 571 | 98 | 0.998 | 0 | TRUE | Sulfite reductase [NADPH] hemoprotein beta-component (EC 1.8.1.2) |
| PGF_08632970 | 23.8 | 573 | 98 | 0.994 | 0 | TRUE | diguanylate cyclase/phosphodiesterase (GGDEF & EAL domains) with PAS/PAC sensor(s) |
| PGF_00421624 | 23.7 | 577 | 98 | 0.987 | 0.012 | TRUE | DNA polymerase X family |
| PGF_00051108 | 23.59 | 562 | 98 | 0.995 | 0.001 | TRUE | Septation ring formation regulator EzrA |
| PGF_00990926 | 23.43 | 562 | 98 | 0.988 | 0.001 | TRUE | Trehalose-6-phosphate hydrolase (EC 3.2.1.93) |
| PGF_00419557 | 23.19 | 541 | 98 | 0.997 | 0 | TRUE | Copper resistance protein CopC / Copper resistance protein CopD |
| PGF_00423553 | 23.08 | 549 | 98 | 0.985 | 0.006 | TRUE | Dipeptide ABC transporter, substrate-binding protein DppA (TC 3.A.1.5.2) |
| PGF_03294256 | 22.68 | 536 | 98 | 0.979 | 0.017 | TRUE | PTS system, maltose-specific IIC component / PTS system, maltose-specific IIB component (EC 2.7.1.208) |
| PGF_04294677 | 22.56 | 518 | 98 | 0.991 | 0.002 | TRUE | 2-isopropylmalate synthase (EC 2.3.3.13) |
| PGF_07491427 | 22.52 | 509 | 98 | 0.998 | 0 | TRUE | Inner membrane protein YqiK |
| PGF_00056814 | 22.49 | 515 | 98 | 0.991 | 0 | TRUE | Anthranilate synthase, aminase component (EC 4.1.3.27) |
| PGF_00013509 | 22.49 | 512 | 98 | 0.994 | 0 | TRUE | IMP cyclohydrolase (EC 3.5.4.10) / Phosphoribosylaminoimidazolecarboxamide formyltransferase (EC 2.1.2.3) |
| PGF_01675349 | 22.48 | 510 | 98 | 0.996 | 0 | TRUE | Purine nucleoside ABC transporter, ATP-binding protein |
| PGF_00013418 | 22.32 | 501 | 98 | 0.997 | 0 | TRUE | Oxygen-independent coproporphyrinogen-III oxidase-like protein HemZ |
| PGF_00420806 | 22.31 | 503 | 98 | 0.995 | 0 | TRUE | D-alanine--poly(phosphoribitol) ligase subunit 1 (EC 6.1.1.13) |
| PGF_00007119 | 22.25 | 513 | 98 | 0.982 | 0.001 | TRUE | Galactose-1-phosphate uridylyltransferase (EC 2.7.7.10) |
| PGF_00054087 | 22.18 | 492 | 98 | 1 | 0 | TRUE | Stage IV sporulation protein A |
| PGF_05424184 | 22.11 | 496 | 98 | 0.993 | 0 | TRUE | Na(+) H(+) antiporter subunit D |
| PGF_00402817 | 22.09 | 490 | 98 | 0.998 | 0 | TRUE | Betaine aldehyde dehydrogenase (EC 1.2.1.8) |
| PGF_00962420 | 22.08 | 490 | 98 | 0.998 | 0 | TRUE | Arginine decarboxylase (EC 4.1.1.19) |
| PGF_00008337 | 21.94 | 483 | 98 | 0.998 | 0 | TRUE | Glutamyl-tRNA synthetase (EC 6.1.1.17) @ Glutamyl-tRNA(Gln) synthetase (EC 6.1.1.24) |
| PGF_10345122 | 21.81 | 479 | 98 | 0.997 | 0 | TRUE | Lactate utilization protein LutB |
| PGF_00066854 | 21.76 | 475 | 98 | 0.998 | 0 | TRUE | Aspartate ammonia-lyase (EC 4.3.1.1) |
| PGF_00956915 | 21.74 | 486 | 98 | 0.986 | 0 | TRUE | O-succinylbenzoic acid--CoA ligase (EC 6.2.1.26) |
| PGF_00008876 | 21.7 | 484 | 98 | 0.987 | 0 | TRUE | Glycogen synthase, ADP-glucose transglucosylase (EC 2.4.1.21) |
| PGF_00012901 | 21.69 | 473 | 98 | 0.997 | 0 | TRUE | Hydroxyaromatic non-oxidative decarboxylase protein C (EC 4.1.1.-) |
| PGF_03068639 | 21.54 | 471 | 98 | 0.993 | 0.002 | TRUE | Amino-acid permease RocC |
| PGF_03446029 | 21.54 | 475 | 98 | 0.988 | 0.01 | TRUE | PTS system, trehalose-specific IIB component (EC 2.7.1.201) / PTS system, trehalose-specific IIC component |
| PGF_06132116 | 21.49 | 463 | 98 | 0.999 | 0 | TRUE | L-cystine uptake protein TcyP, sodium:anion symporter family |
| PGF_00035888 | 21.45 | 470 | 98 | 0.989 | 0 | TRUE | Predicted glycolate dehydrogenase, 2-subunit type (EC 1.1.99.14), iron-sulfur subunit GlcD |
| PGF_00064101 | 21.41 | 464 | 98 | 0.994 | 0.001 | TRUE | UDP-glucose dehydrogenase in teichuronic acid synthesis TuaD (EC 1.1.1.22) |
| PGF_02620298 | 21.37 | 461 | 98 | 0.995 | 0 | TRUE | Argininosuccinate lyase (EC 4.3.2.1) |
| PGF_10483952 | 21.31 | 459 | 98 | 0.995 | 0 | TRUE | PTS system, IIB component / PTS system, IIC component |
| PGF_00064600 | 21.24 | 475 | 98 | 0.975 | 0.02 | TRUE | Uncharacterized RNA methyltransferase YfjO |
| PGF_02648683 | 21.24 | 456 | 98 | 0.995 | 0.002 | TRUE | PTS system, N-acetylmuramic acid-specific IIB component (EC 2.7.1.192) / PTS system, N-acetylmuramic acid-specific IIC component |
| PGF_03405430 | 21.24 | 455 | 98 | 0.996 | 0 | TRUE | D-glucarate transporter |
| PGF_03318742 | 21.23 | 452 | 98 | 0.999 | 0 | TRUE | PTS system, N-acetylglucosamine-specific IIC component / PTS system, N-acetylglucosamine-specific IIB component (EC 2.7.1.193) |
| PGF_04560429 | 21.23 | 451 | 98 | 1 | 0 | TRUE | Mg/Co/Ni transporter MgtE, CBS domain-containing |
| PGF_00053892 | 21.18 | 450 | 98 | 0.998 | 0 | TRUE | Spore germination protein YpeB |
| PGF_00066263 | 21.13 | 479 | 98 | 0.966 | 0.019 | TRUE | Uronate isomerase (EC 5.3.1.12) |
| PGF_00880747 | 21.12 | 453 | 98 | 0.992 | 0 | TRUE | Na+/H+ antiporter NhaC |
| PGF_00008774 | 21.11 | 448 | 98 | 0.997 | 0 | TRUE | Glycine dehydrogenase [decarboxylating] (glycine cleavage system P1 protein) (EC 1.4.4.2) |
| PGF_00184655 | 21.09 | 448 | 98 | 0.997 | 0 | TRUE | hypothetical protein |
| PGF_00053945 | 21.05 | 445 | 98 | 0.998 | 0 | TRUE | Sporulation protein YkvU |
| PGF_00769755 | 21.05 | 447 | 98 | 0.995 | 0 | TRUE | 16S rRNA (cytosine(967)-C(5))-methyltransferase (EC 2.1.1.176) |
| PGF_03146251 | 20.91 | 450 | 98 | 0.985 | 0.002 | TRUE | Uric acid permease PucJ |
| PGF_00028525 | 20.89 | 441 | 98 | 0.995 | 0.004 | TRUE | Oxaloacetate decarboxylase involved in citrate fermentation (EC 4.1.1.3) |
| PGF_00007024 | 20.84 | 436 | 98 | 0.998 | 0 | TRUE | GTP-binding protein EngA |
| PGF_00058560 | 20.84 | 521 | 98 | 0.913 | 0.08 | TRUE | Transcriptional regulator GabR of GABA utilization (GntR family with aminotransferase-like domain) |
| PGF_00421032 | 20.81 | 448 | 98 | 0.983 | 0 | TRUE | D-serine ammonia-lyase (EC 4.3.1.18) |
| PGF_00066502 | 20.76 | 431 | 98 | 1 | 0 | TRUE | Asparaginyl-tRNA synthetase (EC 6.1.1.22) |
| PGF_06935032 | 20.73 | 430 | 98 | 1 | 0 | TRUE | Adenylosuccinate synthetase (EC 6.3.4.4) |
| PGF_00064241 | 20.72 | 441 | 98 | 0.987 | 0 | TRUE | UPF0214 protein YfeW |
| PGF_01054379 | 20.71 | 431 | 98 | 0.998 | 0 | TRUE | Adenylosuccinate lyase (EC 4.3.2.2) @ SAICAR lyase (EC 4.3.2.2) |
| PGF_03064306 | 20.68 | 428 | 98 | 0.999 | 0 | TRUE | Sporulation kinase C (EC 2.7.13.3) |
| PGF_00066695 | 20.67 | 443 | 98 | 0.982 | 0 | TRUE | Vitamin B12 ABC transporter, ATP-binding protein BtuD / Adenosylcobinamide amidohydrolase (EC 3.5.1.90) |
| PGF_09188652 | 20.64 | 430 | 98 | 0.995 | 0 | TRUE | Molybdopterin molybdenumtransferase (EC 2.10.1.1) |
| PGF_02939833 | 20.63 | 434 | 98 | 0.99 | 0.001 | TRUE | UPF0053 membrane protein YrkA |
| PGF_00001028 | 20.61 | 428 | 98 | 0.996 | 0 | TRUE | Uncharacterized zinc protease YmfH |
| PGF_06833830 | 20.6 | 436 | 98 | 0.987 | 0 | TRUE | hypothetical protein |
| PGF_02452671 | 20.55 | 424 | 98 | 0.998 | 0 | TRUE | Cell division trigger factor (EC 5.2.1.8) |
| PGF_09398028 | 20.54 | 422 | 98 | 1 | 0 | TRUE | Uncharacterized protease YrrO |
| PGF_00015701 | 20.53 | 423 | 98 | 0.998 | 0 | TRUE | Isocitrate dehydrogenase [NADP] (EC 1.1.1.42) |
| PGF_03004613 | 20.51 | 424 | 98 | 0.996 | 0 | TRUE | Histidyl-tRNA synthetase (EC 6.1.1.21) |
| PGF_03515627 | 20.49 | 426 | 98 | 0.993 | 0 | TRUE | hypothetical protein |
| PGF_03685660 | 20.45 | 428 | 98 | 0.988 | 0.002 | TRUE | Uncharacterized MFS-type transporter YxiO |
| PGF_00049731 | 20.45 | 421 | 98 | 0.996 | 0 | TRUE | Aluminum resistance protein |
| PGF_00066935 | 20.44 | 466 | 98 | 0.947 | 0.045 | TRUE | Uracil permease @ Uracil:proton symporter UraA |
| PGF_01124177 | 20.33 | 417 | 98 | 0.996 | 0 | TRUE | Dihydrolipoamide succinyltransferase component (E2) of 2-oxoglutarate dehydrogenase complex (EC 2.3.1.61) |
| PGF_05387084 | 20.25 | 415 | 98 | 0.994 | 0 | TRUE | Gamma-glutamyl phosphate reductase (EC 1.2.1.41) |
| PGF_01443078 | 20.18 | 409 | 98 | 0.998 | 0 | TRUE | Predicted signal transduction protein |
| PGF_00736716 | 20.15 | 412 | 98 | 0.993 | 0 | TRUE | hypothetical protein |
| PGF_00021976 | 20.06 | 404 | 98 | 0.998 | 0 | TRUE | Uncharacterized metal ion transporter YcsG, Mn(2+)/Fe(2+) NRAMP family |
| PGF_03202156 | 20 | 400 | 98 | 1 | 0 | TRUE | S-adenosylmethionine synthetase (EC 2.5.1.6) |
| PGF_00092388 | 19.94 | 410 | 98 | 0.985 | 0.006 | TRUE | hypothetical protein |
| PGF_00034602 | 19.82 | 393 | 98 | 1 | 0 | TRUE | Poly-gamma-glutamate synthase subunit PgsB/CapB (EC 6.3.2.-) |
| PGF_02944756 | 19.74 | 391 | 98 | 0.998 | 0 | TRUE | 3-ketoacyl-CoA thiolase [fadN-fadA-fadE operon] (EC 2.3.1.16) |
| PGF_00023189 | 19.73 | 397 | 98 | 0.99 | 0 | TRUE | Uncharacterized MFS-type transporter YttB |
| PGF_10555225 | 19.67 | 412 | 98 | 0.969 | 0.022 | TRUE | Ornithine aminotransferase (EC 2.6.1.13) |
| PGF_00403927 | 19.67 | 393 | 98 | 0.992 | 0 | TRUE | Biosynthetic Aromatic amino acid aminotransferase alpha (EC 2.6.1.57) @ Aspartate aminotransferase (EC 2.6.1.1) |
| PGF_09155108 | 19.63 | 391 | 98 | 0.993 | 0 | TRUE | Exonuclease SbcD |
| PGF_00054051 | 19.6 | 411 | 98 | 0.967 | 0.029 | TRUE | Stage III sporulation protein AE |
| PGF_00417840 | 19.52 | 396 | 98 | 0.981 | 0.016 | TRUE | Chorismate synthase (EC 4.2.3.5) |
| PGF_00056157 | 19.51 | 389 | 98 | 0.989 | 0 | TRUE | Teichuronic acid biosynthesis glycosyl transferase TuaC |
| PGF_10149521 | 19.47 | 380 | 98 | 0.999 | 0 | TRUE | Glucose-1-phosphate adenylyltransferase (EC 2.7.7.27) |
| PGF_00027514 | 19.46 | 385 | 98 | 0.992 | 0 | TRUE | N-acetylornithine aminotransferase (EC 2.6.1.11) |
| PGF_09438139 | 19.39 | 378 | 98 | 0.998 | 0 | TRUE | Flagellar motor switch protein FliN |
| PGF_00032869 | 19.38 | 380 | 98 | 0.994 | 0 | TRUE | Isovaleryl-CoA dehydrogenase (EC 1.3.8.4) |
| PGF_04991657 | 19.38 | 379 | 98 | 0.995 | 0 | TRUE | Oxygen-independent coproporphyrinogen-III oxidase-like protein YggW |
| PGF_03048863 | 19.37 | 377 | 98 | 0.997 | 0 | TRUE | Response regulator aspartate phosphatase B |
| PGF_03028706 | 19.36 | 377 | 98 | 0.997 | 0 | TRUE | Spore coat protein CotSA |
| PGF_00420020 | 19.35 | 380 | 98 | 0.992 | 0.003 | TRUE | Cystathionine gamma-lyase (EC 4.4.1.1) @ Homocysteine desulfhydrase (EC 4.4.1.2) |
| PGF_00008313 | 19.34 | 415 | 98 | 0.949 | 0.042 | TRUE | Glutamine-dependent 2-keto-4-methylthiobutyrate transaminase |
| PGF_02653253 | 19.28 | 378 | 98 | 0.992 | 0.003 | TRUE | ABC transporter, RND-adapter-like protein YknX |
| PGF_00024274 | 19.27 | 380 | 98 | 0.989 | 0 | TRUE | N5-carboxyaminoimidazole ribonucleotide synthase (EC 6.3.4.18) |
| PGF_07072582 | 19.26 | 371 | 98 | 1 | 0 | TRUE | RNA polymerase sigma factor RpoD |
| PGF_07063065 | 19.26 | 371 | 98 | 1 | 0 | TRUE | Transcription termination protein NusA |
| PGF_00502238 | 19.26 | 371 | 98 | 1 | 0 | TRUE | Pyruvate dehydrogenase E1 component alpha subunit (EC 1.2.4.1) |
| PGF_00024656 | 19.22 | 382 | 98 | 0.983 | 0.005 | TRUE | Uncharacterized NADH-dependent flavin oxidoreductase YqiG |
| PGF_00404036 | 19.21 | 410 | 98 | 0.949 | 0.035 | TRUE | Biotin biosynthesis cytochrome P450 (EC 1.14.15.12) |
| PGF_04871820 | 19.21 | 371 | 98 | 0.997 | 0 | TRUE | Prephenate dehydrogenase (EC 1.3.1.12) |
| PGF_00722427 | 19.18 | 373 | 98 | 0.993 | 0 | TRUE | hypothetical protein |
| PGF_00007012 | 19.11 | 366 | 98 | 0.999 | 0 | TRUE | GTP-binding and nucleic acid-binding protein YchF |
| PGF_00056316 | 19.1 | 368 | 98 | 0.996 | 0 | TRUE | Tetraprenyl-beta-curcumene synthase (EC 4.2.3.130) |
| PGF_00016850 | 19.06 | 364 | 98 | 0.999 | 0 | TRUE | Branched-chain amino acid dehydrogenase [deaminating] (EC 1.4.1.9)(EC 1.4.1.23) |
| PGF_04146410 | 19.05 | 363 | 98 | 1 | 0 | TRUE | Protein-arginine kinase McsB (EC 2.7.14.1) |
| PGF_00408522 | 19.05 | 371 | 98 | 0.989 | 0 | TRUE | Putative isomerase YitF |
| PGF_00007560 | 19.04 | 366 | 98 | 0.995 | 0 | TRUE | Germination (Cortex hydrolysis) and sporulation protein GerM |
| PGF_03961783 | 19.03 | 365 | 98 | 0.996 | 0 | TRUE | Uncharacterized protein YvlB |
| PGF_00754446 | 18.99 | 410 | 98 | 0.938 | 0.048 | TRUE | Uncharacterized MFS-type transporter YceJ |
| PGF_09945671 | 18.98 | 433 | 98 | 0.912 | 0.085 | TRUE | Acetate kinase (EC 2.7.2.1) |
| PGF_00037527 | 18.95 | 364 | 98 | 0.993 | 0.001 | TRUE | Proline dipeptidase (EC 3.4.13.9) |
| PGF_00069766 | 18.95 | 365 | 98 | 0.992 | 0 | TRUE | Type III polyketide sythase producing alkylpyrones (B. subtilis BpsA) |
| PGF_01756633 | 18.94 | 399 | 98 | 0.948 | 0.03 | TRUE | Ferrichrome transport system permease protein FhuB |
| PGF_00828128 | 18.93 | 361 | 98 | 0.996 | 0 | TRUE | Putative aminopeptidase YsdC |
| PGF_04858171 | 18.93 | 363 | 98 | 0.993 | 0.001 | TRUE | Branched-chain acyl kinase |
| PGF_00014295 | 18.91 | 362 | 98 | 0.994 | 0 | TRUE | Inner spore coat protein CotH |
| PGF_00030640 | 18.87 | 356 | 98 | 1 | 0 | TRUE | Peptide chain release factor 1 |
| PGF_08308923 | 18.86 | 357 | 98 | 0.998 | 0 | TRUE | Sigma-M negative effector |
| PGF_02516666 | 18.83 | 356 | 98 | 0.998 | 0 | TRUE | Cytochrome c oxidase polypeptide II (EC 1.9.3.1) |
| PGF_00054982 | 18.71 | 354 | 98 | 0.994 | 0 | TRUE | Sulfate permease, Pit-type |
| PGF_00053851 | 18.65 | 351 | 98 | 0.996 | 0 | TRUE | Spore coat protein CotS |
| PGF_00028828 | 18.62 | 356 | 98 | 0.987 | 0 | TRUE | Uncharacterized oxidoreductase YxnA |
| PGF_09679949 | 18.57 | 358 | 98 | 0.981 | 0.005 | TRUE | DNA alkylation repair enzyme |
| PGF_07915158 | 18.52 | 346 | 98 | 0.996 | 0 | TRUE | N(6)-L-threonylcarbamoyladenine synthase (EC 2.3.1.234) |
| PGF_00811541 | 18.49 | 347 | 98 | 0.993 | 0 | TRUE | Uncharacterized membrane protein YkvI |
| PGF_00758189 | 18.43 | 344 | 98 | 0.994 | 0 | TRUE | hypothetical protein |
| PGF_07889681 | 18.39 | 345 | 98 | 0.99 | 0 | TRUE | N-acetyl-gamma-glutamyl-phosphate reductase (EC 1.2.1.38) |
| PGF_00247656 | 18.38 | 340 | 98 | 0.997 | 0 | TRUE | Putative membrane-bound acyltransferase YkrP |
| PGF_00417381 | 18.37 | 340 | 98 | 0.997 | 0 | TRUE | Central glycolytic genes regulator |
| PGF_00648450 | 18.36 | 337 | 98 | 1 | 0 | TRUE | Rod shape-determining protein MreB |
| PGF_00008864 | 18.35 | 345 | 98 | 0.988 | 0.006 | TRUE | Glycogen biosynthesis protein GlgD, glucose-1-phosphate adenylyltransferase family |
| PGF_00927902 | 18.33 | 348 | 98 | 0.983 | 0.002 | TRUE | hypothetical protein |
| PGF_00024478 | 18.3 | 335 | 98 | 1 | 0 | TRUE | NAD-dependent glyceraldehyde-3-phosphate dehydrogenase (EC 1.2.1.12) |
| PGF_00025057 | 18.27 | 355 | 98 | 0.97 | 0.018 | TRUE | NTD biosynthesis operon putative oxidoreductase NtdC (EC 1.-.-.-) |
| PGF_00037171 | 18.24 | 333 | 98 | 1 | 0 | TRUE | Probable low-affinity inorganic phosphate transporter |
| PGF_00689473 | 18.2 | 361 | 98 | 0.958 | 0.029 | TRUE | Allergen V5/Tpx-1 related |
| PGF_02944779 | 18.19 | 333 | 98 | 0.997 | 0 | TRUE | Acetoin dehydrogenase E1 component alpha-subunit (EC 2.3.1.190) |
| PGF_03198682 | 18.16 | 335 | 98 | 0.992 | 0 | TRUE | Protease IV |
| PGF_10315399 | 18.15 | 339 | 98 | 0.986 | 0 | TRUE | Oxidoreductase, zinc-binding dehydrogenase family (EC 1.1.1.-) |
| PGF_00907539 | 18.13 | 330 | 98 | 0.998 | 0 | TRUE | Branched-chain alpha-keto acid dehydrogenase, E1 component, alpha subunit (EC 1.2.4.4) |
| PGF_03677192 | 18.13 | 331 | 98 | 0.997 | 0 | TRUE | Lipoate-protein ligase A |
| PGF_00008611 | 18.12 | 345 | 98 | 0.975 | 0.001 | TRUE | Glycerol-3-phosphate dehydrogenase [NAD(P)+] (EC 1.1.1.94) |
| PGF_00412879 | 18.09 | 329 | 98 | 0.998 | 0 | TRUE | Putative glycosyltransferase CsbB |
| PGF_00412178 | 18.08 | 327 | 98 | 1 | 0 | TRUE | Branched-chain alpha-keto acid dehydrogenase, E1 component, beta subunit (EC 1.2.4.4) |
| PGF_02881645 | 18.08 | 332 | 98 | 0.992 | 0.006 | TRUE | Transcriptional regulator GanR, LacI family |
| PGF_00070244 | 18.04 | 331 | 98 | 0.991 | 0 | TRUE | BH0638 unknown conserved protein |
| PGF_01975849 | 18.02 | 337 | 98 | 0.982 | 0 | TRUE | (R)-2-hydroxyacid dehydrogenase, similar to L-sulfolactate dehydrogenase (EC 1.1.1.272) |
| PGF_05399159 | 18.01 | 325 | 98 | 0.999 | 0 | TRUE | Acetyl-coenzyme A carboxyl transferase alpha chain (EC 6.4.1.2) |
| PGF_00417233 | 17.93 | 332 | 98 | 0.984 | 0.007 | TRUE | L-Ala--D-Glu endopeptidase |
| PGF_06874967 | 17.89 | 322 | 98 | 0.997 | 0 | TRUE | [4Fe-4S]-AdoMet protein YtqA |
| PGF_06547054 | 17.86 | 322 | 98 | 0.996 | 0 | TRUE | Ribose ABC transporter, permease protein RbsC (TC 3.A.1.2.1) |
| PGF_09753067 | 17.86 | 322 | 98 | 0.995 | 0 | TRUE | Uncharacterized membrane protein YqjA |
| PGF_02614796 | 17.82 | 324 | 98 | 0.99 | 0.003 | TRUE | Uncharacterized membrane protein YkoY |
| PGF_00178344 | 17.79 | 320 | 98 | 0.995 | 0.001 | TRUE | Putative sporulation hydrolase CotR |
| PGF_00869466 | 17.76 | 317 | 98 | 0.998 | 0 | TRUE | Transcriptional activator protein med |
| PGF_00033289 | 17.72 | 340 | 98 | 0.961 | 0.029 | TRUE | Phosphate:acyl-ACP acyltransferase PlsX (EC 2.3.1.n2) |
| PGF_00416436 | 17.7 | 314 | 98 | 0.999 | 0.001 | TRUE | 3'->5' exoribonuclease Bsu YhaM |
| PGF_12795189 | 17.7 | 324 | 98 | 0.983 | 0 | TRUE | Transcriptional regulator, LysR family |
| PGF_02191019 | 17.68 | 325 | 98 | 0.981 | 0 | TRUE | Thiamine-monophosphate kinase (EC 2.7.4.16) |
| PGF_00069761 | 17.68 | 318 | 98 | 0.991 | 0.001 | TRUE | Cephalosporin-C deacetylase (EC 3.1.1.41) |
| PGF_00064754 | 17.67 | 319 | 98 | 0.989 | 0.009 | TRUE | Uncharacterized iron compound ABC uptake transporter, permease protein |
| PGF_10097367 | 17.65 | 314 | 98 | 0.996 | 0 | TRUE | Porphobilinogen deaminase (EC 2.5.1.61) |
| PGF_00064130 | 17.64 | 316 | 98 | 0.992 | 0 | TRUE | UDP-glucuronate 5'-epimerase (EC 5.1.3.12) |
| PGF_04645019 | 17.6 | 310 | 98 | 1 | 0 | TRUE | HPr kinase/phosphorylase |
| PGF_00423732 | 17.59 | 317 | 98 | 0.988 | 0.009 | TRUE | 4-hydroxy-3-methylbut-2-enyl diphosphate reductase (EC 1.17.7.4) |
| PGF_00071514 | 17.53 | 348 | 98 | 0.94 | 0.045 | TRUE | Uncharacterized protein YceB |
| PGF_05165078 | 17.48 | 317 | 98 | 0.982 | 0.002 | TRUE | Methionyl-tRNA formyltransferase (EC 2.1.2.9) |
| PGF_00760084 | 17.45 | 307 | 98 | 0.996 | 0 | TRUE | Cystathionine beta-synthase (EC 4.2.1.22) |
| PGF_04835795 | 17.43 | 304 | 98 | 1 | 0 | TRUE | Site-specific tyrosine recombinase XerC |
| PGF_00012288 | 17.43 | 315 | 98 | 0.982 | 0.002 | TRUE | Homocysteine S-methyltransferase (EC 2.1.1.10) |
| PGF_00017894 | 17.42 | 308 | 98 | 0.993 | 0 | TRUE | LysR-family transcriptional regulator Bsu YtlI |
| PGF_02907590 | 17.4 | 312 | 98 | 0.985 | 0.001 | TRUE | Uncharacterized transporter YcxC, EamA family |
| PGF_00064755 | 17.4 | 317 | 98 | 0.977 | 0.001 | TRUE | Uncharacterized iron compound ABC uptake transporter, substrate-binding protein |
| PGF_00063974 | 17.39 | 303 | 98 | 0.999 | 0 | TRUE | UDP-N-acetylenolpyruvoylglucosamine reductase (EC 1.3.1.98) |
| PGF_00058767 | 17.38 | 302 | 98 | 1 | 0 | TRUE | Transcriptional regulator of alpha-acetolactate operon AlsR |
| PGF_00031207 | 17.38 | 305 | 98 | 0.995 | 0.001 | TRUE | Uncharacterized transporter YvbV, EamA family |
| PGF_00064857 | 17.38 | 306 | 98 | 0.993 | 0 | TRUE | Uncharacterized oxidoreductase YqkF |
| PGF_00007027 | 17.34 | 301 | 98 | 0.999 | 0 | TRUE | GTP-binding protein Era |
| PGF_00071153 | 17.34 | 311 | 98 | 0.983 | 0 | TRUE | Bacillus subtilis catabolite repression transcription factor CcpB |
| PGF_01496598 | 17.32 | 303 | 98 | 0.995 | 0.001 | TRUE | Chemotaxis protein CheV (EC 2.7.3.-) |
| PGF_02390924 | 17.32 | 318 | 98 | 0.971 | 0.022 | TRUE | 16S rRNA (cytosine(1402)-N(4))-methyltransferase (EC 2.1.1.199) |
| PGF_10569727 | 17.31 | 303 | 98 | 0.994 | 0 | TRUE | LSU rRNA pseudouridine(1911/1915/1917) synthase (EC 5.4.99.23) |
| PGF_02346669 | 17.29 | 335 | 98 | 0.945 | 0.047 | TRUE | Ornithine carbamoyltransferase (EC 2.1.3.3) |
| PGF_03125357 | 17.26 | 304 | 98 | 0.99 | 0 | TRUE | O-Methyltransferase involved in polyketide biosynthesis |
| PGF_00425662 | 17.24 | 299 | 98 | 0.997 | 0.001 | TRUE | FIG00002411: Thioredoxin-fold protein |
| PGF_12669939 | 17.2 | 305 | 98 | 0.985 | 0.005 | TRUE | Ribose ABC transporter, substrate-binding protein RbsB (TC 3.A.1.2.1) |
| PGF_09162729 | 17.2 | 298 | 98 | 0.996 | 0 | TRUE | Flagellar hook-associated protein FlgL |
| PGF_00037828 | 17.17 | 298 | 98 | 0.994 | 0 | TRUE | Protease HtpX |
| PGF_10357700 | 17.16 | 302 | 98 | 0.988 | 0 | TRUE | Similar to ribosomal large subunit pseudouridine synthase D, Bacillus subtilis YhcT type |
| PGF_09221959 | 17.16 | 301 | 98 | 0.989 | 0 | TRUE | Methylisocitrate lyase (EC 4.1.3.30) |
| PGF_03889350 | 17.1 | 294 | 98 | 0.998 | 0 | TRUE | Uncharacterized protein YgxA |
| PGF_05797639 | 17.08 | 296 | 98 | 0.993 | 0 | TRUE | Predicted rhamnose oligosaccharide ABC transport system, permease component |
| PGF_00734140 | 17.06 | 297 | 98 | 0.99 | 0 | TRUE | hypothetical protein |
| PGF_03136713 | 17.06 | 292 | 98 | 0.998 | 0 | TRUE | Efflux ABC transporter, ATP-binding protein |
| PGF_00017897 | 17.03 | 293 | 98 | 0.995 | 0 | TRUE | LysR-family transcriptional regulator Bsu YwqM |
| PGF_00549380 | 17 | 290 | 98 | 0.999 | 0 | TRUE | 4-hydroxy-tetrahydrodipicolinate synthase (EC 4.3.3.7) |
| PGF_00017893 | 16.98 | 290 | 98 | 0.997 | 0 | TRUE | LysR-family transcriptional regulator BsdA |
| PGF_00006359 | 16.94 | 299 | 98 | 0.98 | 0 | TRUE | Fructokinase (EC 2.7.1.4) |
| PGF_02920362 | 16.93 | 287 | 98 | 0.999 | 0 | TRUE | Cell wall-binding protein YocH |
| PGF_00057415 | 16.9 | 288 | 98 | 0.996 | 0 | TRUE | Transcription antiterminator GlcT |
| PGF_07528295 | 16.89 | 289 | 98 | 0.994 | 0 | TRUE | hypothetical protein |
| PGF_02904727 | 16.89 | 287 | 98 | 0.997 | 0 | TRUE | hypothetical protein |
| PGF_04047965 | 16.87 | 293 | 98 | 0.985 | 0.003 | TRUE | hypothetical protein |
| PGF_00020984 | 16.82 | 283 | 98 | 1 | 0 | TRUE | Methenyltetrahydrofolate cyclohydrolase (EC 3.5.4.9) / Methylenetetrahydrofolate dehydrogenase (NADP+) (EC 1.5.1.5) |
| PGF_00064796 | 16.81 | 294 | 98 | 0.981 | 0.013 | TRUE | Uncharacterized membrane protein Bsu0528 (YdeO) |
| PGF_04872827 | 16.79 | 283 | 98 | 0.998 | 0 | TRUE | Chromosome (plasmid) partitioning protein ParB-2 |
| PGF_05682204 | 16.77 | 282 | 98 | 0.999 | 0 | TRUE | Glycine betaine ABC transport system, permease protein OpuAB |
| PGF_00426701 | 16.74 | 281 | 98 | 0.998 | 0 | TRUE | FIG005590: DegV family protein |
| PGF_02971400 | 16.73 | 280 | 98 | 1 | 0 | TRUE | Uncharacterized oxidoreductase YtbE |
| PGF_00059807 | 16.72 | 283 | 98 | 0.994 | 0 | TRUE | Uncharacterized transcriptional regulator YbbH, RpiR family |
| PGF_03150126 | 16.72 | 285 | 98 | 0.99 | 0 | TRUE | hypothetical protein |
| PGF_00409415 | 16.72 | 282 | 98 | 0.996 | 0.003 | TRUE | UPF0750 membrane protein YvjA |
| PGF_00026864 | 16.72 | 281 | 98 | 0.997 | 0 | TRUE | Octanoyl-[GcvH]:protein N-octanoyltransferase (EC 2.3.1.204) |
| PGF_00426877 | 16.71 | 282 | 98 | 0.995 | 0 | TRUE | LSU ribosomal maturation GTPase RbgA (B. subtilis YlqF) |
| PGF_00419444 | 16.68 | 290 | 98 | 0.979 | 0 | TRUE | Conserved protein LiaG in B. subtilis in Lia cluster |
| PGF_00047141 | 16.66 | 281 | 98 | 0.994 | 0 | TRUE | Alpha-arabinosides ABC transport system, permease protein AraQ |
| PGF_00049914 | 16.64 | 282 | 98 | 0.991 | 0.002 | TRUE | STAS domain protein |
| PGF_00016393 | 16.64 | 277 | 98 | 1 | 0 | TRUE | LSU ribosomal protein L2p (L8e) |
| PGF_00064875 | 16.64 | 285 | 98 | 0.986 | 0.001 | TRUE | Uncharacterized phosphatase YwpJ |
| PGF_03514578 | 16.63 | 277 | 98 | 0.999 | 0 | TRUE | Beta-glucoside bgl operon antiterminator, BglG family |
| PGF_00019066 | 16.63 | 296 | 98 | 0.966 | 0.007 | TRUE | Maltodextrose utilization protein YvdJ |
| PGF_07735780 | 16.62 | 278 | 98 | 0.997 | 0 | TRUE | hypothetical protein |
| PGF_03466304 | 16.61 | 281 | 98 | 0.991 | 0 | TRUE | Quorum-quenching lactonase YtnP |
| PGF_00026863 | 16.58 | 278 | 98 | 0.994 | 0.001 | TRUE | Octanoate-[acyl-carrier-protein]-protein-N-octanoyltransferase, LipM (EC 2.3.1.181) |
| PGF_03589817 | 16.58 | 282 | 98 | 0.987 | 0 | TRUE | Peptidoglycan N-acetylglucosamine deacetylase (EC 3.5.1.-) |
| PGF_00014397 | 16.53 | 278 | 98 | 0.992 | 0 | TRUE | Inosose isomerase (EC 5.3.99.11) |
| PGF_03152833 | 16.53 | 321 | 98 | 0.923 | 0.059 | TRUE | Ku domain protein |
| PGF_01019139 | 16.52 | 280 | 98 | 0.987 | 0.002 | TRUE | Uncharacterized oxidoreductase YusZ |
| PGF_06874321 | 16.48 | 276 | 98 | 0.992 | 0 | TRUE | Multiple sugar ABC transporter, permease protein MsmG |
| PGF_02475999 | 16.47 | 274 | 98 | 0.995 | 0.004 | TRUE | RsbT co-antagonist protein RsbRA |
| PGF_02868448 | 16.46 | 275 | 98 | 0.993 | 0 | TRUE | Uncharacterized transcriptional regulator YrhO, TrmB family |
| PGF_02781328 | 16.46 | 272 | 98 | 0.998 | 0 | TRUE | NAD synthetase (EC 6.3.1.5) |
| PGF_12908096 | 16.45 | 280 | 98 | 0.983 | 0.015 | TRUE | Spermidine synthase (EC 2.5.1.16) |
| PGF_00026300 | 16.43 | 271 | 98 | 0.998 | 0 | TRUE | Novel pyridoxal kinase, thiD family (EC 2.7.1.35) |
| PGF_12832328 | 16.42 | 278 | 98 | 0.985 | 0.002 | TRUE | BmrR regulator of multidrug efflux transporter Bmr |
| PGF_04149001 | 16.4 | 270 | 98 | 0.998 | 0 | TRUE | Hydroxymethylpyrimidine ABC transporter, transmembrane component |
| PGF_00045950 | 16.32 | 272 | 98 | 0.99 | 0 | TRUE | Pyrroline-5-carboxylate reductase (EC 1.5.1.2), ProG-like |
| PGF_03375596 | 16.3 | 270 | 98 | 0.992 | 0 | TRUE | Sugar phosphatase YidA (EC 3.1.3.23) |
| PGF_01176589 | 16.3 | 267 | 98 | 0.997 | 0 | TRUE | 4-hydroxy-tetrahydrodipicolinate reductase (EC 1.17.1.8) |
| PGF_01329082 | 16.3 | 289 | 98 | 0.959 | 0.021 | TRUE | 23S rRNA (guanine(748)-N(1))-methyltransferase (EC 2.1.1.188) |
| PGF_00009882 | 16.29 | 268 | 98 | 0.995 | 0 | TRUE | Uncharacterized glycosyltransferase YwdF |
| PGF_00053997 | 16.29 | 267 | 98 | 0.997 | 0.001 | TRUE | Stage 0 sporulation two-component response regulator (Spo0A) |
| PGF_00026200 | 16.23 | 273 | 98 | 0.982 | 0.007 | TRUE | Non-heme chloroperoxidase (EC 1.11.1.10) |
| PGF_01127158 | 16.23 | 264 | 98 | 0.999 | 0 | TRUE | Zn-dependent hydrolase YycJ/WalJ, required for cell wall metabolism and coordination of cell division with DNA replication |
| PGF_00005450 | 16.22 | 264 | 98 | 0.998 | 0 | TRUE | Flagellar hook protein FlgE |
| PGF_09027360 | 16.19 | 264 | 98 | 0.996 | 0 | TRUE | Regulatory protein RecX |
| PGF_05740384 | 16.16 | 292 | 98 | 0.946 | 0.048 | TRUE | Formamidopyrimidine-DNA glycosylase (EC 3.2.2.23) |
| PGF_03125026 | 16.15 | 271 | 98 | 0.981 | 0.005 | TRUE | Putative uroporphyrinogen-III synthase (EC 4.2.1.75), related to YjjA (in BS) |
| PGF_00083876 | 16.14 | 282 | 98 | 0.961 | 0.003 | TRUE | hypothetical protein |
| PGF_00015827 | 16.09 | 412 | 98 | 0.793 | 0.202 | TRUE | Kef-type K+ transport system, predicted NAD-binding component |
| PGF_00046553 | 16.04 | 260 | 98 | 0.995 | 0.002 | TRUE | RNA polymerase sporulation specific sigma factor SigG |
| PGF_00029831 | 16.03 | 280 | 98 | 0.958 | 0.04 | TRUE | PTS system, fructose-specific IIC component |
| PGF_00024307 | 16.01 | 275 | 98 | 0.965 | 0.03 | TRUE | NAD kinase (EC 2.7.1.23) homolog |
| PGF_05318435 | 16 | 257 | 98 | 0.998 | 0 | TRUE | Electron transfer flavoprotein, beta subunit |
| PGF_00401136 | 16 | 257 | 98 | 0.998 | 0 | TRUE | Uncharacterized membrane protein YceF |
| PGF_00286418 | 15.98 | 258 | 98 | 0.995 | 0 | TRUE | Uncharacterized membrane protein YhfC |
| PGF_00010468 | 15.97 | 257 | 98 | 0.996 | 0 | TRUE | Hydrolase (HAD superfamily) in cluster with DUF1447 |
| PGF_02177520 | 15.97 | 255 | 98 | 1 | 0 | TRUE | RNA polymerase sporulation specific sigma factor SigF |
| PGF_00943546 | 15.96 | 260 | 98 | 0.99 | 0 | TRUE | Methylglutaconyl-CoA hydratase (EC 4.2.1.18) |
| PGF_00876160 | 15.94 | 255 | 98 | 0.998 | 0 | TRUE | Alpha-acetolactate decarboxylase (EC 4.1.1.5) |
| PGF_00419622 | 15.9 | 254 | 98 | 0.998 | 0 | TRUE | Coproheme decarboxylase HemQ (no EC) |
| PGF_00071264 | 15.9 | 253 | 98 | 1 | 0 | TRUE | Bacitracin export ATP-binding protein BceA |
| PGF_08131899 | 15.89 | 256 | 98 | 0.993 | 0 | TRUE | hypothetical protein |
| PGF_00423593 | 15.87 | 259 | 98 | 0.986 | 0.007 | TRUE | Dipeptidyl aminopeptidases/acylaminoacyl-peptidase |
| PGF_03145879 | 15.86 | 279 | 98 | 0.95 | 0.037 | TRUE | L-cystine ABC transporter, substrate-binding protein TcyK |
| PGF_07099142 | 15.85 | 256 | 98 | 0.991 | 0 | TRUE | Uncharacterized transporter YwcJ, NirC family |
| PGF_00011536 | 15.84 | 254 | 98 | 0.994 | 0.002 | TRUE | Heptaprenyl diphosphate synthase component I (EC 2.5.1.30) |
| PGF_00046280 | 15.84 | 251 | 98 | 1 | 0 | TRUE | RNA polymerase heat shock sigma factor SigI |
| PGF_00071195 | 15.82 | 255 | 98 | 0.991 | 0 | TRUE | Dihydroanticapsin 7-dehydrogenase (EC 1.1.1.385) => BacC |
| PGF_12786716 | 15.81 | 254 | 98 | 0.992 | 0 | TRUE | Transcriptional regulator GlvR, RpiR family |
| PGF_04710902 | 15.81 | 260 | 98 | 0.98 | 0.012 | TRUE | Lactam utilization protein LamB |
| PGF_00391863 | 15.77 | 253 | 98 | 0.992 | 0 | TRUE | Uncharacterized protein YtbQ |
| PGF_00060533 | 15.75 | 254 | 98 | 0.989 | 0 | TRUE | Transmembrane component YkoC of energizing module of thiamin-regulated ECF transporter for HydroxyMethylPyrimidine |
| PGF_05689837 | 15.73 | 250 | 98 | 0.995 | 0 | TRUE | Probable iron export permease protein FetB |
| PGF_02144826 | 15.73 | 249 | 98 | 0.997 | 0 | TRUE | FMN reductase [NAD(P)H] (EC 1.5.1.39) |
| PGF_03888286 | 15.73 | 249 | 98 | 0.997 | 0 | TRUE | FMN reductase (NADPH) (EC 1.5.1.38) |
| PGF_00051581 | 15.73 | 252 | 98 | 0.991 | 0 | TRUE | Short-chain dehydrodenase (gene dltE) |
| PGF_03255161 | 15.73 | 248 | 98 | 0.999 | 0 | TRUE | Tyrosine-protein kinase transmembrane modulator EpsC |
| PGF_00209115 | 15.71 | 250 | 98 | 0.993 | 0 | TRUE | hypothetical protein |
| PGF_12663506 | 15.7 | 254 | 98 | 0.985 | 0 | TRUE | hypothetical protein |
| PGF_08789310 | 15.67 | 247 | 98 | 0.997 | 0 | TRUE | L-cystine ABC transporter, ATP-binding protein TcyC |
| PGF_03506091 | 15.61 | 245 | 98 | 0.997 | 0 | TRUE | UPF0721 transmembrane protein YdhB |
| PGF_00048586 | 15.59 | 245 | 98 | 0.996 | 0 | TRUE | Ribonuclease PH (EC 2.7.7.56) |
| PGF_02999829 | 15.58 | 253 | 98 | 0.979 | 0.012 | TRUE | Uncharacterized protein YvpB |
| PGF_00413208 | 15.53 | 243 | 98 | 0.996 | 0 | TRUE | tRNA (guanine(37)-N(1))-methyltransferase (EC 2.1.1.228) |
| PGF_00761454 | 15.52 | 243 | 98 | 0.996 | 0 | TRUE | hypothetical protein |
| PGF_00058741 | 15.52 | 241 | 98 | 1 | 0 | TRUE | Transcriptional regulator in cluster with Zn-dependent hydrolase |
| PGF_00062143 | 15.45 | 242 | 98 | 0.993 | 0.001 | TRUE | Twin-arginine translocation protein TatCd |
| PGF_00064474 | 15.44 | 240 | 98 | 0.997 | 0 | TRUE | Arginine transport ATP-binding protein ArtM |
| PGF_00020322 | 15.35 | 261 | 98 | 0.95 | 0.039 | TRUE | Metal-binding protein ZinT |
| PGF_07474537 | 15.3 | 234 | 98 | 1 | 0 | TRUE | L-cystine ABC transporter, permease protein TcyB |
| PGF_00015991 | 15.22 | 235 | 98 | 0.993 | 0 | TRUE | L-cystine ABC transporter, permease protein TcyM |
| PGF_00378256 | 15.22 | 245 | 98 | 0.972 | 0.013 | TRUE | Uncharacterized membrane anchored protein YpbE |
| PGF_00805747 | 15.18 | 231 | 98 | 0.998 | 0 | TRUE | ABC transporter ATP-binding protein YtrE |
| PGF_00060190 | 15.1 | 229 | 98 | 0.998 | 0 | TRUE | ABC transporter-like sensor linked response regulator YxdJ |
| PGF_00066735 | 15.09 | 245 | 98 | 0.964 | 0.017 | TRUE | Vng1746c |
| PGF_10525342 | 15.04 | 247 | 98 | 0.957 | 0.03 | TRUE | Transcriptional regulator, AraC family |
| PGF_03017026 | 15.02 | 227 | 98 | 0.997 | 0 | TRUE | hypothetical protein |
| PGF_07723617 | 15 | 225 | 98 | 1 | 0 | TRUE | Putative membrane protease YugP |
| PGF_02992152 | 14.93 | 229 | 98 | 0.987 | 0.004 | TRUE | Uncharacterized protein YcbO |
| PGF_03044001 | 14.92 | 226 | 98 | 0.992 | 0 | TRUE | Tricarboxylate transport transcriptional regulator TctD => Mg-citrate response regulator CitT |
| PGF_03066301 | 14.91 | 240 | 98 | 0.963 | 0.029 | TRUE | Uncharacterized protein YobT |
| PGF_00051120 | 14.91 | 229 | 98 | 0.985 | 0.013 | TRUE | Septum site-determining protein MinC |
| PGF_00065865 | 14.91 | 230 | 98 | 0.983 | 0.003 | TRUE | Uncharacterized transcriptional response regulator YcbL |
| PGF_00317190 | 14.88 | 226 | 98 | 0.99 | 0.001 | TRUE | Teichuronic acid biosynthesis protein TuaF |
| PGF_00047734 | 14.85 | 223 | 98 | 0.994 | 0 | TRUE | Respiratory nitrate reductase gamma chain (EC 1.7.99.4) |
| PGF_00062557 | 14.79 | 235 | 98 | 0.965 | 0.022 | TRUE | Two-component response regulator, malate (EC 2.7.3.-) |
| PGF_00063451 | 14.78 | 221 | 98 | 0.994 | 0 | TRUE | Type III effector HrpW, hairpin with pectate lyase domain |
| PGF_00424244 | 14.78 | 265 | 98 | 0.908 | 0.086 | TRUE | Endo-beta-1,3-1,4 glucanase (licheninase) (EC 3.2.1.73) |
| PGF_06326847 | 14.76 | 221 | 98 | 0.993 | 0.004 | TRUE | Flagellar biosynthesis protein FliP |
| PGF_00052541 | 14.75 | 227 | 98 | 0.979 | 0.003 | TRUE | Amino acid racemase RacX |
| PGF_00425679 | 14.73 | 217 | 98 | 1 | 0 | TRUE | FIG000605: protein co-occurring with transport systems (COG1739) |
| PGF_00038920 | 14.73 | 220 | 98 | 0.993 | 0 | TRUE | Pseudouridine-5' phosphatase (EC 3.1.3.-) |
| PGF_00046555 | 14.71 | 218 | 98 | 0.996 | 0.002 | TRUE | RNA polymerase sporulation specific sigma factor SigH |
| PGF_00067178 | 14.7 | 222 | 98 | 0.986 | 0 | TRUE | Uncharacterized membrane protein YteU |
| PGF_00054072 | 14.69 | 218 | 98 | 0.995 | 0.001 | TRUE | Stage III sporulation protein AH |
| PGF_07320841 | 14.69 | 218 | 98 | 0.995 | 0 | TRUE | ClpCP protease substrate adapter protein MecA |
| PGF_07459774 | 14.65 | 216 | 98 | 0.997 | 0 | TRUE | Succinyl-CoA:3-ketoacid-coenzyme A transferase subunit B (EC 2.8.3.5) |
| PGF_00118588 | 14.65 | 220 | 98 | 0.987 | 0.009 | TRUE | Protease PrsW |
| PGF_00011470 | 14.59 | 213 | 98 | 1 | 0 | TRUE | Hemolysin III |
| PGF_00056362 | 14.57 | 216 | 98 | 0.992 | 0 | TRUE | TPR repeat-containing protein YrrB |
| PGF_03063210 | 14.54 | 213 | 98 | 0.996 | 0 | TRUE | TVP38/TMEM64 family membrane protein YtxB |
| PGF_01776925 | 14.52 | 211 | 98 | 0.999 | 0 | TRUE | Uridine kinase (EC 2.7.1.48) |
| PGF_08089396 | 14.5 | 218 | 98 | 0.982 | 0 | TRUE | Tricarboxylate transport transcriptional regulator TctD |
| PGF_00277008 | 14.46 | 210 | 98 | 0.998 | 0 | TRUE | Uncharacterized membrane protein YrhP |
| PGF_01921960 | 14.46 | 214 | 98 | 0.988 | 0 | TRUE | Uncharacterized sugar epimerase YhfK |
| PGF_00016431 | 14.46 | 209 | 98 | 1 | 0 | TRUE | LSU ribosomal protein L3p (L3e) |
| PGF_00346814 | 14.44 | 212 | 98 | 0.992 | 0 | TRUE | DUF1054 superfamily protein |
| PGF_04861087 | 14.44 | 249 | 98 | 0.915 | 0.071 | TRUE | Two-component response regulator BceR |
| PGF_04867467 | 14.41 | 212 | 98 | 0.989 | 0 | TRUE | Imidazole glycerol phosphate synthase amidotransferase subunit HisH |
| PGF_02445053 | 14.38 | 208 | 98 | 0.997 | 0 | TRUE | Uncharacterized protein YkvT, NOT involved in spore germination |
| PGF_03810679 | 14.32 | 205 | 98 | 1 | 0 | TRUE | SOS-response repressor and protease LexA (EC 3.4.21.88) |
| PGF_01756668 | 14.32 | 205 | 98 | 1 | 0 | TRUE | UPF0111 protein YkaA, likely to be a phosphate transport regulator |
| PGF_00027684 | 14.32 | 211 | 98 | 0.986 | 0.006 | TRUE | Acetyltransferase AcuA, acetyl-CoA synthetase inhibitor |
| PGF_00819204 | 14.3 | 221 | 98 | 0.962 | 0.021 | TRUE | Uncharacterized DedA family protein YkoX |
| PGF_00015822 | 14.3 | 205 | 98 | 0.999 | 0 | TRUE | KapD, inhibitor of KinA pathway to sporulation |
| PGF_10458966 | 14.23 | 209 | 98 | 0.984 | 0.006 | TRUE | hypothetical protein |
| PGF_00057526 | 14.19 | 210 | 98 | 0.979 | 0.011 | TRUE | Anti-sigma-W factor RsiW |
| PGF_04192284 | 14.19 | 204 | 98 | 0.993 | 0 | TRUE | Conserved membrane protein in copper uptake, YcnI |
| PGF_03299849 | 14.19 | 204 | 98 | 0.993 | 0.001 | TRUE | Hydroxyaromatic non-oxidative decarboxylase protein B (EC 4.1.1.-) |
| PGF_00049893 | 14.14 | 200 | 98 | 1 | 0 | TRUE | SSU ribosomal protein S4p (S9e) @ SSU ribosomal protein S4p (S9e), zinc-independent |
| PGF_10537005 | 14.12 | 218 | 98 | 0.957 | 0.032 | TRUE | Probable metallo-hydrolase YqgX |
| PGF_00730659 | 14.08 | 200 | 98 | 0.995 | 0 | TRUE | Uncharacterized transcriptional response regulator YvfU |
| PGF_02944297 | 14.06 | 198 | 98 | 0.999 | 0 | TRUE | hypothetical protein |
| PGF_00060189 | 14.05 | 288 | 98 | 0.828 | 0.142 | TRUE | Transcriptional regulatory protein YvrH |
| PGF_00924363 | 14.04 | 199 | 98 | 0.995 | 0 | TRUE | Substrate-specific component YkoE of thiamin-regulated ECF transporter for HydroxyMethylPyrimidine |
| PGF_00285620 | 14.03 | 197 | 98 | 1 | 0 | TRUE | ATP-dependent Clp protease proteolytic subunit ClpP (EC 3.4.21.92) |
| PGF_06649360 | 14.02 | 197 | 98 | 0.999 | 0 | TRUE | Segregation and condensation protein B |
| PGF_06671388 | 14.01 | 200 | 98 | 0.991 | 0 | TRUE | Carboxylesterase (EC 3.1.1.1) |
| PGF_00053894 | 14 | 196 | 98 | 1 | 0 | TRUE | Spore maturation protein A |
| PGF_00039048 | 13.98 | 196 | 98 | 0.999 | 0 | TRUE | Putative 3-methyladenine DNA glycosylase |
| PGF_02230794 | 13.98 | 199 | 98 | 0.991 | 0.001 | TRUE | Molybdenum cofactor guanylyltransferase (EC 2.7.7.77) |
| PGF_00045787 | 13.95 | 196 | 98 | 0.996 | 0 | TRUE | Pyridoxal 5'-phosphate synthase (glutamine hydrolyzing), glutaminase subunit (EC 4.3.3.6) |
| PGF_10556767 | 13.95 | 205 | 98 | 0.974 | 0 | TRUE | HD domain protein |
| PGF_00004058 | 13.9 | 194 | 98 | 0.998 | 0 | TRUE | Fatty acid metabolism regulator protein FadR, TetR family |
| PGF_03084240 | 13.9 | 194 | 98 | 0.998 | 0 | TRUE | Uncharacterized transcriptional regulator YvdT, TetR family |
| PGF_01323228 | 13.89 | 193 | 98 | 1 | 0 | TRUE | Acyl-phosphate:glycerol-3-phosphate O-acyltransferase PlsY (EC 2.3.1.n3) |
| PGF_06195014 | 13.88 | 194 | 98 | 0.997 | 0 | TRUE | Putative nitroreductase family protein SACOL0874 |
| PGF_00046367 | 13.87 | 194 | 98 | 0.996 | 0.001 | TRUE | RNA polymerase sigma factor SigX |
| PGF_00420679 | 13.86 | 200 | 98 | 0.98 | 0 | TRUE | Cytoplasmic thiamin-binding component of thiamin ABC transporter, COG0011 family |
| PGF_00026362 | 13.85 | 198 | 98 | 0.984 | 0.008 | TRUE | Nucleoside 5-triphosphatase RdgB (dHAPTP, dITP, XTP-specific) (EC 3.6.1.66) |
| PGF_03887528 | 13.82 | 257 | 98 | 0.862 | 0.13 | TRUE | UPF0702 transmembrane protein YdfR |
| PGF_00012577 | 13.8 | 280 | 98 | 0.825 | 0.124 | TRUE | Uncharacterized protein YmaE |
| PGF_02820109 | 13.77 | 190 | 98 | 0.999 | 0 | TRUE | GTP cyclohydrolase I (EC 3.5.4.16) type 1 |
| PGF_03147263 | 13.72 | 192 | 98 | 0.991 | 0 | TRUE | Metal-sulfur cluster biosynthesis proteins YuaD |
| PGF_08843714 | 13.72 | 189 | 98 | 0.998 | 0 | TRUE | Septum formation protein Maf |
| PGF_00222761 | 13.72 | 238 | 98 | 0.889 | 0.081 | TRUE | Uncharacterized protein YhbD |
| PGF_01677463 | 13.63 | 187 | 98 | 0.997 | 0 | TRUE | RNA polymerase sigma factor SigW |
| PGF_03799365 | 13.6 | 185 | 98 | 1 | 0 | TRUE | Translation elongation factor P |
| PGF_07808527 | 13.56 | 185 | 98 | 0.997 | 0 | TRUE | ADP-ribose pyrophosphatase (EC 3.6.1.13) |
| PGF_00013296 | 13.54 | 186 | 98 | 0.993 | 0 | TRUE | Uncharacterized protein YjgD |
| PGF_00232324 | 13.48 | 184 | 98 | 0.993 | 0 | TRUE | Chromosome-anchoring protein RacA |
| PGF_02897110 | 13.45 | 185 | 98 | 0.989 | 0 | TRUE | 6-phospho-3-hexuloisomerase (EC 5.3.1.27) |
| PGF_00060261 | 13.38 | 180 | 98 | 0.998 | 0 | TRUE | Transcriptional repressor for NAD biosynthesis in gram-positives |
| PGF_00016443 | 13.38 | 179 | 98 | 1 | 0 | TRUE | LSU ribosomal protein L5p (L11e) |
| PGF_00016444 | 13.38 | 179 | 98 | 1 | 0 | TRUE | LSU ribosomal protein L6p (L9e) |
| PGF_00047732 | 13.37 | 184 | 98 | 0.986 | 0 | TRUE | Respiratory nitrate reductase delta chain (EC 1.7.99.4) |
| PGF_00777822 | 13.37 | 185 | 98 | 0.983 | 0.003 | TRUE | hypothetical protein |
| PGF_06274006 | 13.34 | 181 | 98 | 0.992 | 0 | TRUE | ATP synthase delta chain (EC 3.6.3.14) |
| PGF_04457297 | 13.33 | 187 | 98 | 0.975 | 0 | TRUE | 5-formyltetrahydrofolate cyclo-ligase (EC 6.3.3.2) |
| PGF_07133621 | 13.33 | 184 | 98 | 0.983 | 0.004 | TRUE | 16S rRNA (guanine(966)-N(2))-methyltransferase (EC 2.1.1.171) |
| PGF_00053897 | 13.31 | 178 | 98 | 0.998 | 0 | TRUE | Spore maturation protein B |
| PGF_04666784 | 13.31 | 183 | 98 | 0.984 | 0 | TRUE | hypothetical protein |
| PGF_02031958 | 13.29 | 177 | 98 | 0.999 | 0 | TRUE | Possible colicin V production protein |
| PGF_00930759 | 13.19 | 176 | 98 | 0.994 | 0 | TRUE | L,D-transpeptidase |
| PGF_00333474 | 13.14 | 181 | 98 | 0.976 | 0 | TRUE | Protein csk22 |
| PGF_06701488 | 13.14 | 249 | 98 | 0.832 | 0.163 | TRUE | RNA-binding protein Jag |
| PGF_00054042 | 13.03 | 171 | 98 | 0.997 | 0 | TRUE | Stage III sporulation protein AB |
| PGF_10499347 | 13.03 | 173 | 98 | 0.991 | 0 | TRUE | Molybdopterin-guanine dinucleotide biosynthesis protein MobB |
| PGF_00204364 | 13.03 | 175 | 98 | 0.985 | 0 | TRUE | Uncharacterized protein YkkA |
| PGF_00025215 | 13.01 | 174 | 98 | 0.986 | 0.011 | TRUE | Acetolactate synthase small subunit (EC 2.2.1.6) |
| PGF_00007481 | 13 | 172 | 98 | 0.991 | 0 | TRUE | ThiJ/PfpI family protein YhbO |
| PGF_00022467 | 12.97 | 170 | 98 | 0.994 | 0 | TRUE | Molybdenum cofactor biosynthesis protein MoaB |
| PGF_10546429 | 12.96 | 170 | 98 | 0.994 | 0.001 | TRUE | ATP synthase F0 sector subunit b (EC 3.6.3.14) |
| PGF_00413533 | 12.89 | 167 | 98 | 0.997 | 0 | TRUE | Thiol peroxidase, Tpx-type (EC 1.11.1.15) |
| PGF_03174068 | 12.88 | 189 | 98 | 0.937 | 0.062 | TRUE | Transcription antitermination protein NusG |
| PGF_01213071 | 12.87 | 166 | 98 | 0.999 | 0 | TRUE | LSU ribosomal protein L10p (P0) |
| PGF_04653134 | 12.87 | 748 | 98 | 0.47 | 0.271 | TRUE | CDP-glycerol:poly(glycerophosphate) glycerophosphotransferase (EC 2.7.8.12) |
| PGF_00024855 | 12.84 | 165 | 98 | 0.999 | 0 | TRUE | NADPH-dependent 7-cyano-7-deazaguanine reductase (EC 1.7.1.13) |
| PGF_00002432 | 12.81 | 167 | 98 | 0.991 | 0.002 | TRUE | Sporulation membrane protein YtrI |
| PGF_00185080 | 12.78 | 185 | 98 | 0.94 | 0.011 | TRUE | hypothetical protein |
| PGF_00065803 | 12.78 | 166 | 98 | 0.992 | 0 | TRUE | Uncharacterized protein, homolog of B.subtilis yhgC |
| PGF_00804722 | 12.67 | 165 | 98 | 0.987 | 0.002 | TRUE | Uncharacterized protein YocC |
| PGF_10386969 | 12.65 | 186 | 98 | 0.928 | 0.054 | TRUE | Acyl-CoA hydrolase (EC 3.1.2.20) |
| PGF_01949670 | 12.64 | 163 | 98 | 0.99 | 0.006 | TRUE | PTS system, fructose-specific IIB component (EC 2.7.1.202) |
| PGF_00033019 | 12.63 | 161 | 98 | 0.995 | 0 | TRUE | Phenolic acid decarboxylase (EC 4.1.1.-) |
| PGF_02927704 | 12.61 | 168 | 98 | 0.973 | 0 | TRUE | hypothetical protein |
| PGF_00412811 | 12.59 | 160 | 98 | 0.995 | 0 | TRUE | Spore coat protein CotF |
| PGF_01446424 | 12.53 | 158 | 98 | 0.997 | 0 | TRUE | Na(+) H(+) antiporter subunit E |
| PGF_00035451 | 12.51 | 162 | 98 | 0.983 | 0.004 | TRUE | Precorrin-2 oxidase (EC 1.3.1.76) |
| PGF_03119533 | 12.51 | 158 | 98 | 0.995 | 0 | TRUE | hypothetical protein |
| PGF_00049433 | 12.5 | 157 | 98 | 0.998 | 0 | TRUE | S-ribosylhomocysteine lyase (EC 4.4.1.21) @ Autoinducer-2 production protein LuxS |
| PGF_07311230 | 12.49 | 156 | 98 | 1 | 0 | TRUE | Positive regulator of CheA protein activity (CheW) |
| PGF_01682834 | 12.48 | 156 | 98 | 0.999 | 0 | TRUE | Bacterial ribosome SSU maturation protein RimP |
| PGF_02715281 | 12.46 | 160 | 98 | 0.985 | 0.001 | TRUE | Uncharacterized membrane protein YoaS |
| PGF_00401663 | 12.46 | 157 | 98 | 0.994 | 0 | TRUE | Transcriptional regulator of the azlBCD operon, AsnC family |
| PGF_00169629 | 12.44 | 191 | 98 | 0.9 | 0.082 | TRUE | Uncharacterized membrane protein YuaF |
| PGF_00027979 | 12.42 | 156 | 98 | 0.995 | 0 | TRUE | Uncharacterized N-acetyltransferase YqjY |
| PGF_03889881 | 12.41 | 154 | 98 | 1 | 0 | TRUE | Transcriptional regulator CtsR |
| PGF_00707140 | 12.36 | 159 | 98 | 0.98 | 0.004 | TRUE | Cys-tRNA(Pro) deacylase YbaK |
| PGF_00022550 | 12.35 | 157 | 98 | 0.986 | 0.004 | TRUE | Molybdopterin synthase catalytic subunit MoaE (EC 2.8.1.12) |
| PGF_00151857 | 12.33 | 178 | 98 | 0.924 | 0.04 | TRUE | hypothetical protein |
| PGF_03090398 | 12.31 | 152 | 98 | 0.999 | 0 | TRUE | Uncharacterized protein YtxH |
| PGF_03501056 | 12.26 | 151 | 98 | 0.997 | 0 | TRUE | hypothetical protein |
| PGF_03021263 | 12.22 | 151 | 98 | 0.994 | 0 | TRUE | SepF, FtsZ-interacting protein related to cell division |
| PGF_02156631 | 12.18 | 149 | 98 | 0.998 | 0 | TRUE | Arginine pathway regulatory protein ArgR, repressor of arg regulon |
| PGF_10470343 | 12.18 | 149 | 98 | 0.998 | 0 | TRUE | Ferric uptake regulation protein FUR |
| PGF_01724713 | 12.17 | 149 | 98 | 0.997 | 0 | TRUE | Ribose-5-phosphate isomerase B (EC 5.3.1.6) |
| PGF_10054809 | 12.16 | 148 | 98 | 0.999 | 0 | TRUE | Transamidase GatB domain protein |
| PGF_00797662 | 12.12 | 147 | 98 | 1 | 0 | TRUE | CBS domain-containing protein YkuL |
| PGF_00012657 | 12.12 | 147 | 98 | 1 | 0 | TRUE | ACT domain-containing protein |
| PGF_03802161 | 12.1 | 199 | 98 | 0.857 | 0.13 | TRUE | DNA-directed RNA polymerase delta subunit (EC 2.7.7.6) |
| PGF_03147273 | 12.07 | 146 | 98 | 0.999 | 0 | TRUE | Nitrite-sensitive transcriptional repressor NsrR |
| PGF_00362441 | 12.04 | 164 | 98 | 0.94 | 0.042 | TRUE | hypothetical protein |
| PGF_01222424 | 12.04 | 194 | 98 | 0.864 | 0.124 | TRUE | DinB protein |
| PGF_00002428 | 12.01 | 148 | 98 | 0.987 | 0 | TRUE | Uncharacterized protein YpiF |
| PGF_06594013 | 11.96 | 144 | 98 | 0.997 | 0.001 | TRUE | Peptide-methionine (R)-S-oxide reductase MsrB (EC 1.8.4.12) |
| PGF_00183711 | 11.96 | 145 | 98 | 0.993 | 0 | TRUE | hypothetical protein |
| PGF_10545696 | 11.94 | 148 | 98 | 0.982 | 0 | TRUE | hypothetical protein |
| PGF_09755850 | 11.93 | 143 | 98 | 0.997 | 0 | TRUE | Na(+) H(+) antiporter subunit B |
| PGF_03518570 | 11.92 | 148 | 98 | 0.98 | 0.01 | TRUE | Nucleoside diphosphate kinase (EC 2.7.4.6) |
| PGF_01465362 | 11.92 | 142 | 98 | 1 | 0 | TRUE | Mn-dependent transcriptional regulator MntR |
| PGF_07695531 | 11.87 | 141 | 98 | 1 | 0 | TRUE | LSU ribosomal protein L11p (L12e) |
| PGF_00799099 | 11.87 | 141 | 98 | 1 | 0 | TRUE | Uncharacterized transcriptional regulator YpoP, MarR family |
| PGF_01602421 | 11.8 | 140 | 98 | 0.997 | 0 | TRUE | Flagellar basal-body rod modification protein FlgD |
| PGF_03994339 | 11.75 | 161 | 98 | 0.926 | 0.053 | TRUE | hypothetical protein |
| PGF_10488938 | 11.75 | 138 | 98 | 1 | 0 | TRUE | Putative pre-16S rRNA nuclease YqgF |
| PGF_00053855 | 11.67 | 172 | 98 | 0.89 | 0.013 | TRUE | Spore coat protein CotX |
| PGF_00070331 | 11.63 | 174 | 98 | 0.882 | 0.067 | TRUE | Uncharacterized protein YrrD |
| PGF_00020996 | 11.63 | 137 | 98 | 0.994 | 0 | TRUE | Methylglyoxal synthase (EC 4.2.3.3) |
| PGF_00404745 | 11.63 | 139 | 98 | 0.986 | 0 | TRUE | prolyl endopeptidase |
| PGF_09345013 | 11.62 | 139 | 98 | 0.986 | 0.004 | TRUE | Iron-sulfur cluster regulator IscR |
| PGF_00026337 | 11.59 | 136 | 98 | 0.994 | 0.003 | TRUE | Sporulation-specific extracellular nuclease NucB |
| PGF_03074981 | 11.59 | 136 | 98 | 0.994 | 0 | TRUE | hypothetical protein |
| PGF_00007489 | 11.57 | 146 | 98 | 0.958 | 0.041 | TRUE | UPF0478 protein YtxG |
| PGF_01447607 | 11.55 | 135 | 98 | 0.994 | 0 | TRUE | Uncharacterized CoA-binding protein YneT |
| PGF_03147810 | 11.54 | 134 | 98 | 0.997 | 0 | TRUE | Putative oxidoreductase CatD |
| PGF_00175215 | 11.52 | 135 | 98 | 0.991 | 0 | TRUE | hypothetical protein |
| PGF_00195004 | 11.43 | 143 | 98 | 0.956 | 0.017 | TRUE | hypothetical protein |
| PGF_02019584 | 11.42 | 131 | 98 | 0.998 | 0 | TRUE | Regulatory protein Spx |
| PGF_06348445 | 11.42 | 135 | 98 | 0.983 | 0.015 | TRUE | Anti-sigma B factor RsbT |
| PGF_00373500 | 11.4 | 131 | 98 | 0.996 | 0 | TRUE | hypothetical protein |
| PGF_12835789 | 11.31 | 131 | 98 | 0.989 | 0.008 | TRUE | UPF0297 protein YrzL |
| PGF_00012257 | 11.31 | 128 | 98 | 1 | 0 | TRUE | Holin-like protein CidA |
| PGF_09008105 | 11.25 | 127 | 98 | 0.998 | 0 | TRUE | Chorismate mutase II (EC 5.4.99.5) |
| PGF_00013273 | 11.24 | 128 | 98 | 0.994 | 0 | TRUE | Uncharacterized protein YaeR with similarity to glyoxylase family |
| PGF_07192369 | 11.21 | 128 | 98 | 0.991 | 0 | TRUE | Uncharacterized protein YlqD |
| PGF_00309250 | 11.15 | 145 | 98 | 0.926 | 0.023 | TRUE | hypothetical protein |
| PGF_00149187 | 11.15 | 137 | 98 | 0.953 | 0.036 | TRUE | hypothetical protein |
| PGF_00424261 | 11.14 | 124 | 98 | 1 | 0 | TRUE | Uncharacterized protein YurQ |
| PGF_00064808 | 11.13 | 215 | 98 | 0.759 | 0.188 | TRUE | Uncharacterized membrane protein YwmF |
| PGF_12850351 | 11.11 | 179 | 98 | 0.83 | 0.149 | TRUE | hypothetical protein |
| PGF_00016445 | 11.09 | 123 | 98 | 1 | 0 | TRUE | LSU ribosomal protein L7p/L12p (P1/P2) |
| PGF_00015820 | 11.01 | 128 | 98 | 0.974 | 0 | TRUE | KapB, lipoprotein required for KinB pathway to sporulation |
| PGF_00016604 | 10.98 | 128 | 98 | 0.971 | 0.011 | TRUE | Lactoylglutathione lyase and related lyases |
| PGF_00035192 | 10.95 | 126 | 98 | 0.976 | 0 | TRUE | Possible glyoxylase family protein (Lactoylglutathione lyase) (EC 4.4.1.5) |
| PGF_00419571 | 10.9 | 187 | 98 | 0.797 | 0.152 | FALSE | Copper resistance transcriptional regulator CueR (MerR family) |
| PGF_00365237 | 10.86 | 123 | 98 | 0.979 | 0.011 | FALSE | hypothetical protein |
| PGF_00483477 | 10.83 | 123 | 98 | 0.977 | 0.009 | FALSE | General stress protein |
| PGF_00057198 | 10.79 | 117 | 98 | 0.997 | 0 | FALSE | Anti-sigma F factor antagonist |
| PGF_00341463 | 10.78 | 118 | 98 | 0.992 | 0 | FALSE | hypothetical protein |
| PGF_02612776 | 10.77 | 117 | 98 | 0.996 | 0 | FALSE | Uncharacterized membrane anchored protein YqzD |
| PGF_00435430 | 10.75 | 118 | 98 | 0.989 | 0 | FALSE | Ribosomal silencing factor RsfA |
| PGF_00023165 | 10.73 | 117 | 98 | 0.992 | 0 | FALSE | Multidrug resistance protein EbrB |
| PGF_04845029 | 10.72 | 115 | 98 | 1 | 0 | FALSE | LSU ribosomal protein L19p |
| PGF_00007480 | 10.72 | 115 | 98 | 0.999 | 0 | FALSE | General stress protein 17M |
| PGF_03754184 | 10.63 | 113 | 98 | 1 | 0 | FALSE | Na(+) H(+) antiporter subunit C |
| PGF_00393175 | 10.61 | 134 | 98 | 0.916 | 0.075 | FALSE | hypothetical protein |
| PGF_00798491 | 10.57 | 128 | 98 | 0.934 | 0 | FALSE | Spore coat protein CotV |
| PGF_02038611 | 10.56 | 112 | 98 | 0.998 | 0 | FALSE | Broad-specificity multidrug efflux pump YkkC |
| PGF_00345138 | 10.53 | 114 | 98 | 0.986 | 0.003 | FALSE | Uncharacterized protein YpuD |
| PGF_00314509 | 10.52 | 115 | 98 | 0.981 | 0 | FALSE | Uncharacterized protein YunG |
| PGF_00071327 | 10.52 | 113 | 98 | 0.99 | 0 | FALSE | Flagellar biosynthesis protein FliT |
| PGF_00092921 | 10.51 | 112 | 98 | 0.994 | 0.003 | FALSE | Uncharacterized membrane protein YtvB |
| PGF_00323454 | 10.37 | 108 | 98 | 0.998 | 0 | FALSE | Uncharacterized membrane protein YvlA |
| PGF_00827960 | 10.36 | 114 | 98 | 0.971 | 0.001 | FALSE | Uncharacterized protein YdgD |
| PGF_10306761 | 10.33 | 125 | 98 | 0.924 | 0.044 | FALSE | hypothetical protein |
| PGF_00748659 | 10.32 | 130 | 98 | 0.905 | 0.05 | FALSE | hypothetical protein |
| PGF_06876517 | 10.25 | 107 | 98 | 0.991 | 0 | FALSE | hypothetical protein |
| PGF_02611684 | 10.24 | 105 | 98 | 0.999 | 0 | FALSE | Uncharacterized membrane protein YlaH |
| PGF_00067418 | 10.19 | 108 | 98 | 0.981 | 0.002 | FALSE | YuzD-like protein |
| PGF_00056802 | 10.15 | 108 | 98 | 0.977 | 0.018 | FALSE | Thioredoxin-like protein YdbP |
| PGF_00188261 | 10.15 | 153 | 98 | 0.821 | 0.091 | FALSE | Uncharacterized protein YncE |
| PGF_00016377 | 10.15 | 103 | 98 | 1 | 0 | FALSE | LSU ribosomal protein L24p (L26e) |
| PGF_00689521 | 10.09 | 103 | 98 | 0.994 | 0 | FALSE | Rhodanese-like domain protein |
| PGF_00426531 | 10.01 | 121 | 98 | 0.91 | 0.057 | FALSE | 5-hydroxyisourate hydrolase (EC 3.5.2.17) |
| PGF_08331244 | 10 | 149 | 98 | 0.819 | 0.173 | FALSE | Diacylglycerol kinase (EC 2.7.1.107) |
| PGF_00002320 | 9.99 | 100 | 98 | 0.999 | 0 | FALSE | Uncharacterized membrane protein YtpI |
| PGF_00009992 | 9.97 | 126 | 98 | 0.888 | 0.051 | FALSE | Uncharacterized protein YsfE |
| PGF_00417093 | 9.9 | 98 | 98 | 1 | 0 | FALSE | Cell division protein GpsB, coordinates the switch between cylindrical and septal cell wall synthesis by re-localization of PBP1 |
| PGF_00844267 | 9.86 | 140 | 98 | 0.834 | 0.149 | FALSE | hypothetical protein |
| PGF_00411944 | 9.8 | 100 | 98 | 0.98 | 0.01 | FALSE | Ribosomal protein L7Ae family protein YlxQ |
| PGF_00355707 | 9.8 | 96 | 98 | 1 | 0 | FALSE | hypothetical protein |
| PGF_00381050 | 9.77 | 99 | 98 | 0.982 | 0 | FALSE | Uncharacterized protein YneQ |
| PGF_00400553 | 9.74 | 95 | 98 | 0.999 | 0 | FALSE | lin0150 |
| PGF_00016385 | 9.7 | 94 | 98 | 1 | 0 | FALSE | LSU ribosomal protein L27p |
| PGF_01534844 | 9.68 | 96 | 98 | 0.988 | 0 | FALSE | Uncharacterized membrane protein YdzA |
| PGF_00037481 | 9.64 | 93 | 98 | 1 | 0 | FALSE | Programmed cell death antitoxin YdcD |
| PGF_00142062 | 9.64 | 93 | 98 | 1 | 0 | FALSE | Uncharacterized protein YqfC |
| PGF_00027563 | 9.64 | 93 | 98 | 0.999 | 0 | FALSE | UPF0473 protein YrzB |
| PGF_00178044 | 9.49 | 90 | 98 | 1 | 0 | FALSE | SSU ribosomal protein S16p |
| PGF_10367439 | 9.47 | 91 | 98 | 0.992 | 0 | FALSE | Acylphosphate phosphohydrolase (EC 3.6.1.7) |
| PGF_02876969 | 9.43 | 89 | 98 | 0.999 | 0 | FALSE | hypothetical protein |
| PGF_02177759 | 9.41 | 93 | 98 | 0.976 | 0.001 | FALSE | Putative glutaredoxin YtnI |
| PGF_00013347 | 9.38 | 88 | 98 | 1 | 0 | FALSE | UPF0297 protein YrzL |
| PGF_00752241 | 9.35 | 122 | 98 | 0.847 | 0.122 | FALSE | hypothetical protein |
| PGF_00049854 | 9.33 | 87 | 98 | 1 | 0 | FALSE | SSU ribosomal protein S17p (S11e) |
| PGF_00940841 | 9.33 | 87 | 98 | 1 | 0 | FALSE | Polypeptide composition of the spore coat protein CotJB |
| PGF_00803437 | 9.33 | 87 | 98 | 1 | 0 | FALSE | Spore morphogenesis and germination protein YwcE |
| PGF_00150056 | 9.33 | 87 | 98 | 1 | 0 | FALSE | Uncharacterized membrane protein YubF |
| PGF_02011114 | 9.33 | 88 | 98 | 0.994 | 0 | FALSE | UPF0223 protein YktA |
| PGF_06573156 | 9.31 | 88 | 98 | 0.993 | 0 | FALSE | Negative regulator of flagellin synthesis FlgM (anti-sigma28) |
| PGF_00098326 | 9.26 | 90 | 98 | 0.976 | 0.011 | FALSE | Uncharacterized protein YpzA |
| PGF_00000336 | 9.22 | 85 | 98 | 1 | 0 | FALSE | Uncharacterized protein YmxH |
| PGF_00837914 | 9.11 | 84 | 98 | 0.994 | 0 | FALSE | hypothetical protein |
| PGF_02708531 | 9.09 | 83 | 98 | 0.998 | 0 | FALSE | Small, acid-soluble spore protein Tlp |
| PGF_08975963 | 9.05 | 82 | 98 | 1 | 0 | FALSE | Firmicutes ribosomal L7Ae family protein |
| PGF_00797917 | 9.03 | 83 | 98 | 0.991 | 0 | FALSE | Uncharacterized protein YodI |
| PGF_03516369 | 8.99 | 82 | 98 | 0.992 | 0 | FALSE | hypothetical protein |
| PGF_05144993 | 8.96 | 82 | 98 | 0.989 | 0.005 | FALSE | Protein CotJA |
| PGF_00651558 | 8.95 | 123 | 98 | 0.807 | 0.185 | FALSE | Uncharacterized protein YisB |
| PGF_03521020 | 8.89 | 79 | 98 | 1 | 0 | FALSE | Uncharacterized protein YkuJ |
| PGF_00003447 | 8.83 | 78 | 98 | 0.999 | 0 | FALSE | Uncharacterized protein YusG |
| PGF_02981302 | 8.75 | 77 | 98 | 0.997 | 0.001 | FALSE | Uncharacterized protein YhzC |
| PGF_07487229 | 8.71 | 77 | 98 | 0.993 | 0.001 | FALSE | Acyl carrier protein |
| PGF_00057429 | 8.66 | 75 | 98 | 1 | 0 | FALSE | Transcription attenuation protein MtrB |
| PGF_02162834 | 8.66 | 77 | 98 | 0.987 | 0.013 | FALSE | Protein CsbA |
| PGF_00197443 | 8.49 | 72 | 98 | 1 | 0 | FALSE | hypothetical protein |
| PGF_01170345 | 8.42 | 71 | 98 | 0.999 | 0 | FALSE | Flagellar protein FlbD |
| PGF_10444145 | 8.37 | 70 | 98 | 1 | 0 | FALSE | ATP synthase F0 sector subunit c (EC 3.6.3.14) |
| PGF_00045655 | 8.35 | 70 | 98 | 0.998 | 0 | FALSE | Sporulation protein YhaL |
| PGF_00038255 | 8.31 | 69 | 98 | 1 | 0 | FALSE | Protein of unknown function DUF1447 |
| PGF_02611683 | 8.27 | 69 | 98 | 0.995 | 0.002 | FALSE | Uncharacterized protein YlaI |
| PGF_06429692 | 8.22 | 68 | 98 | 0.997 | 0.001 | FALSE | Uncharacterized protein CAC3725 |
| PGF_00034746 | 8.17 | 69 | 98 | 0.983 | 0 | FALSE | MbtH-like NRPS chaperone => Polymyxin synthetase PmxB |
| PGF_02612004 | 8.12 | 66 | 98 | 1 | 0 | FALSE | Uncharacterized protein YycD |
| PGF_00053045 | 8 | 64 | 98 | 1 | 0 | FALSE | Small, acid-soluble spore protein D |
| PGF_00268855 | 7.93 | 63 | 98 | 0.999 | 0 | FALSE | Uncharacterized protein YbxH |
| PGF_03278866 | 7.87 | 62 | 98 | 1 | 0 | FALSE | LSU ribosomal protein L28p @ LSU ribosomal protein L28p, zinc-independent |
| PGF_00322934 | 7.87 | 63 | 98 | 0.991 | 0 | FALSE | ComG operon repressor ComZ |
| PGF_00301971 | 7.87 | 62 | 98 | 0.999 | 0 | FALSE | hypothetical protein |
| PGF_00147658 | 7.81 | 61 | 98 | 1 | 0 | FALSE | Uncharacterized protein YfjT |
| PGF_02708606 | 7.8 | 81 | 98 | 0.867 | 0.101 | FALSE | Uncharacterized protein YdjO |
| PGF_00710965 | 7.79 | 74 | 98 | 0.906 | 0.08 | FALSE | hypothetical protein |
| PGF_00341484 | 7.77 | 61 | 98 | 0.994 | 0 | FALSE | Uncharacterized membrane protein YjzD |
| PGF_09669766 | 7.68 | 59 | 98 | 1 | 0 | FALSE | Protein translocase subunit SecE |
| PGF_00016395 | 7.68 | 59 | 98 | 1 | 0 | FALSE | LSU ribosomal protein L30p (L7e) |
| PGF_00827523 | 7.6 | 58 | 98 | 0.998 | 0 | FALSE | Uncharacterized protein YpfB |
| PGF_10323639 | 7.52 | 111 | 98 | 0.714 | 0.24 | FALSE | NifU-like domain protein |
| PGF_03719593 | 7.35 | 54 | 98 | 1 | 0 | FALSE | hypothetical protein |
| PGF_10444495 | 7.23 | 59 | 98 | 0.942 | 0.05 | FALSE | UPF0057 membrane protein YqaE |
| PGF_03996481 | 7.22 | 57 | 98 | 0.957 | 0.036 | FALSE | hypothetical protein |
| PGF_00724879 | 7.14 | 51 | 98 | 0.999 | 0 | FALSE | hypothetical protein |
| PGF_00142906 | 7.09 | 51 | 98 | 0.993 | 0 | FALSE | Uncharacterized protein YkzB |
| PGF_00357665 | 7.05 | 63 | 98 | 0.888 | 0.06 | FALSE | hypothetical protein |
| PGF_00116891 | 6.78 | 46 | 98 | 1 | 0 | FALSE | hypothetical protein |
| PGF_00054280 | 6.69 | 84 | 98 | 0.729 | 0.259 | FALSE | Stress response protein CsbD |
| PGF_00764121 | 6.55 | 63 | 98 | 0.826 | 0.13 | FALSE | hypothetical protein |
| PGF_00304830 | 6.4 | 41 | 98 | 1 | 0 | FALSE | hypothetical protein |
| PGF_10397186 | 6.24 | 40 | 98 | 0.987 | 0 | FALSE | hypothetical protein |
| PGF_00725534 | 6.14 | 38 | 98 | 0.996 | 0 | FALSE | PhrG, regulator of the activity of phosphatase RapG |
| PGF_00731821 | 6.08 | 37 | 98 | 1 | 0 | FALSE | hypothetical protein |
| PGF_00370194 | 6.02 | 60 | 98 | 0.777 | 0.214 | FALSE | hypothetical protein |
| PGF_00666322 | 5.87 | 90 | 98 | 0.619 | 0.359 | FALSE | Uncharacterized protein YpmA |
| PGF_01931072 | 4.74 | 96 | 98 | 0.483 | 0.427 | FALSE | Uncharacterized protein YhfH |
